# Tp53 determines the spatial dynamics of M1/M2 tumor-associated macrophages and M1-driven tumoricidal effects

**DOI:** 10.1038/s41419-025-07346-0

**Published:** 2025-01-22

**Authors:** Yi-Jing Hsiao, Min-Shu Hsieh, Gee-Chen Chang, Yin-Chen Hsu, Chia-Yu Wang, Yan-Ming Chen, Yi-Ling Chen, Pan-Chyr Yang, Sung-Liang Yu

**Affiliations:** 1 Department of Clinical and Laboratory Sciences and Medical Biotechnology, National Taiwan University College of Medicine, Taipei, Taiwan; 2 Institute of Chemistry, Academia Sinica, Taipei, Taiwan; 3 Department of Pathology, National Taiwan University Hospital, Taipei, Taiwan; 4 Division of Pulmonary Medicine, Department of Internal Medicine, Chung Shan Medical University Hospital Taichung, Taichung, Taiwan; 5 Institute of Medicine, Chung Shan Medical University, Taichung, Taiwan; 6 Faculty of Medicine, School of Medicine, National Yang-Ming University, Taipei, Taiwan; 7 Institute of Biomedical Sciences, National Chung Hsing University, Taichung, Taiwan; 8 Division of Chest Medicine, Department of Internal Medicine, Taichung Veterans General Hospital, Taichung, Taiwan; 9 Department of Internal Medicine, National Taiwan University Hospital, Taipei, Taiwan; 10 Institute of Biomedical Sciences, Academia Sinica, Taipei, Taiwan; 11 Graduate Institute of Pathology, National Taiwan University College of Medicine, Taipei, Taiwan; 12 Department of Laboratory Medicine, National Taiwan University Hospital, Taipei, Taiwan; 13 Graduate Institute of Medical Device and Imaging, National Taiwan University College of Medicine, Taipei, Taiwan; 14 Graduate School of Advanced Technology, National Taiwan University, Taipei, Taiwan

**Keywords:** Cancer microenvironment, Monocytes and macrophages, Apoptosis, Non-small-cell lung cancer

## Abstract

The spatial role of M1 and M2 tumor-associated macrophages (M1/M2 TAMs) in precision medicine remains unclear. EGFR and TP53 are among the most frequently mutated genes in lung adenocarcinoma. We characterized the mutation status and density of M1/M2 TAMs within tumor islets and stroma in 117 lung adenocarcinomas using next-generation sequencing and immunohistochemistry, respectively. Stromal M1 TAMs were positively correlated with disease progression and smoking history. In contrast, islet M1/M2 TAMs were predominantly found in tumors with wild-type TP53 (wtp53) but not associated with EGFR status. The presence of wtp53 was associated with the spatial distribution of M1/M2 TAMs in tumor islets and stroma. Additionally, dominance of islet M1 TAMs and M1-signature were significantly associated with improved survival in patients with wtp53 lung adenocarcinoma, unlike in those with mutant TP53. Conditioned medium from M1 macrophages (M1 CM) induced apoptosis in wtp53 cells through increased p53 accumulation. We found that interferons in M1 CM activate JAK1/TYK2 via IFNARs, leading to enhanced STAT1 expression and Y701 phosphorylation. This activation facilitates p53-STAT1 interactions, reduces the interaction between p53 and MDM2, and subsequently decreases p53 ubiquitination. M1 CM inhibited tumorigenesis, and silencing p53 reduced the anti-tumor efficacy of polyinosinic:polycytidylic acid (poly I:C) in vivo. Furthermore, higher M1-signature was significantly associated with better responses and survival following anti-PD1 treatment in wtp53 melanomas. IFNs/STAT1/p53 signaling was critical for the anti-tumor activity of M1 macrophages. These findings suggest that p53 modulates the spatial balance of M1/M2 TAMs, and the tumoricidal effects of M1 TAMs depend on p53 status. Thus, p53 companion diagnostics could facilitate the development of M1-oriented therapies, which may be particularly beneficial for wtp53 patients when combined with immunotherapy.

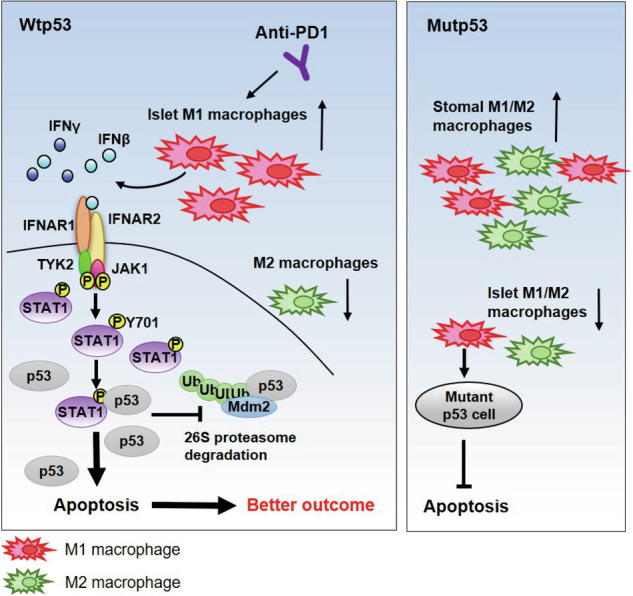

## Introduction

Growing evidence suggests that subtypes of tumor-associated macrophages (TAMs) and genetic mutations are closely related to cancer progression and treatment responses. However, the reciprocal relationships and molecular regulations among TAM phenotypes, genetic alterations, and their impact on lung cancer are not well understood. TAMs play a significant role in tumor progression due to their high prevalence among tumor-infiltrating immune cells and their remarkable plasticity [1, 2]. Proinflammatory M1 macrophages possess anti-tumor abilities and hold therapeutic promise, whereas pro-tumorigenic M2 macrophages suppress the immune response and promote malignancy [2, 3]. Researches indicated that an elevated presence of M2 and TAMs in the tumor stroma is associated with poor outcomes, whereas increased M1 and TAMs in the tumor nest are linked to better prognosis in non-small cell lung cancer (NSCLC) [4–6]. Additionally, the ratio of islet to stromal CD68 TAMs has been identified as an independent factor for overall survival (OS) [7, 8]. Obviously, both the intratumoral distribution and specific subtypes of TAMs are critical factors influencing lung cancer prognosis, but few studies have simultaneously investigated the balance and compared the ratio of islet to stromal M1/M2 TAMs. While macrophage-reprogramming clinical trials have increased in recent years, with nearly 200 agents tested in over 700 trials, a key barrier to their clinical success is the low response rate [9]. Issues like eligible patient selection and TAM localization may contribute to this. Therefore, investigating the distinct roles of M1 and M2 TAMs within the complex tumor microenvironment, particularly in relation to different gene mutations, is crucial for addressing these clinical setbacks.

EGFR mutation is a prominent driver in lung adenocarcinoma and serves as a primary target for targeted therapies. These mutations contribute to the development of an immunosuppressive microenvironment, thereby diminishing the effectiveness of immunotherapy [10, 11]. An analysis of The Cancer Genome Atlas Lung Adenocarcinoma (TCGA-LUAD) dataset revealed a lower proportion of M1 TAMs and a higher proportion of M2 TAMs in EGFR mutant tumors compared to those with wild-type EGFR [12]. The tumor suppressor p53 is by far the most frequently mutated gene among solid tumors. The p53 ablation promotes malignancy by skewing M1 macrophages polarization into the M2 subtype [13, 14]. The p53 restoration in lung adenocarcinoma induces cellular senescence and results in an accumulation of foam macrophages, but the precise functions and subtypes of these macrophages remain unclear [15]. The relationship between gene mutations in EGFR or p53 and the orchestration of M1/M2 TAMs in lung adenocarcinoma is not fully understood.

In this study, we investigate the spatial distribution of M1/M2 TAMs in lung adenocarcinomas with p53 or EGFR mutations. We aim to elucidate the relationships among TAMs, gene mutations, and clinical outcomes. Our research delves into the impact of M1 macrophages on tumor cells and explores the underlying signaling mechanisms in vitro. Additionally, we assess the tumoricidal effects of M1 macrophages and the effect of current immunomodulatory drug in vivo. The study demonstrates the critical role of M1/M2 TAMs dynamics in precision medicine, emphasizing their interplay in influencing patients’ outcome.

## Results

### Tp53 mediates spatial distribution of M1/M2 tumor-associated macrophages

**Fig. 1:**
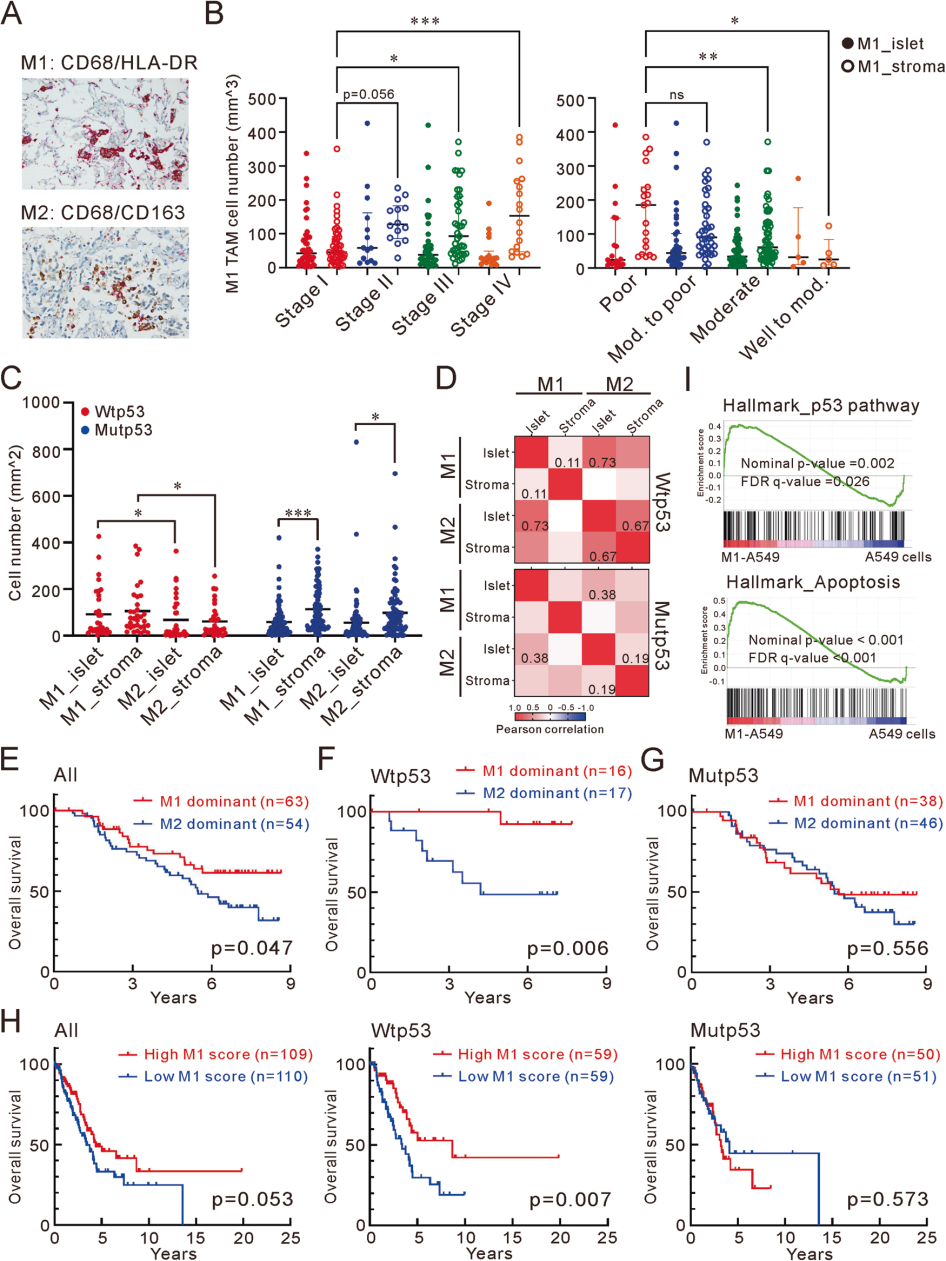
M1 TAM dominance associated with improved outcomes in wtp53 lung adenocarcinomas. **A** Representative immunohistochemistry images showcasing double staining for M1 and M2 TAMs. CD68 (brown) and HLA-DR (red) were the markers for M1 TAMs quantification, while CD68 (brown) and CD163 (red) were used for M2 TAMs. Original magnification ×400. **B** The density of M1 TAMs within tumor islets and stroma across stage I–IV (left) and tumor differentiation (right). *P*-value determined by one-way ANOVA with Tukey’s post hoc test. **C** Comparative distribution of islet and stromal M1/M2 TAMs in wtp53 and mutp53 tumors. *P*-value determined by paired Student’s *t*-test. **D** The correlations between the islet and stromal of M1/M2 TAMs. The correlation was measured by Pearson correlation coefficient. **E** Kaplan-Meier survival analysis for 117 lung adenocarcinomas. The patients are categorized based on the ratio of islet to stromal M1/M2 TAMs. M1 dominance is indicated by a ratio of M1 > M2; M2 dominance is indicated by a ratio of M1 < M2. **F** Kaplan-Meier survival analysis for 33 lung adenocarcinomas with wtp53 genotype. **G** Kaplan-Meier survival analysis for 84 lung adenocarcinomas with mutp53 genotype. **H** Kaplan-Meier survival curves derived from the TCGA-LUAD database. Analyses were separately performed for subgroups with wtp53 (middle) and mutp53 (right) cases. **I** Genes regulated by M1 macrophages in A549 cells enriched in p53 and apoptosis pathways. *P*-value for overall survival was determined by log-rank test. ns not significant, *P*-value > 0.05; *P-value < 0.05; **P-value < 0.01; ***P-value < 0.001.

With M1 TAMs estimably enriched in EGFR wild-type tumors [12], we estimated the immune cell proportions in wild-type and mutant p53 tumors using the TCGA-LUAD database. M1 TAMs were significantly enriched in wild-type p53 (wtp53) tumors (*P* < 0.0001) (Supplementary Fig. 1A). Further analyzing the cytokines known to induce M1/M2 TAM polarization [16], TGF-β and CSF-1, which are reported to promote M2 TAM polarization and infiltration [17, 18], were significantly upregulated in mutp53 tumors (Supplementary Fig. 1B). However, the spatial distribution could not be estimated in silico. To explore the relationship between gene mutations and TAM distribution, we analyzed both EGFR and TP53 mutation statuses in 117 adenocarcinomas and quantified M1 and M2 TAMs through immunohistochemistry (IHC) staining (Supplementary Fig. 1C). Cells doubly positive for CD68 and HLA-DR were classified as M1 TAMs, while those positive for CD68 and CD163 were identified as M2 TAMs (Fig. 1A). Stromal M1 TAMs were associated with smoking, cancer staging, and differentiation, showing significant elevation in higher cancer stages and poorer differentiation; however, stromal M2 showed decreased in stage IV (Fig. 1B and Supplementary Table 1). Notably, TP53 mutation status significantly influenced the presence of M1/M2 TAMs within tumor islets but not EGFR mutations (Table 1 and Supplementary Table 2). A comparison between wtp53 and mutp53 revealed distinct distributions of M1 and M2 TAMs. In mutp53 tumors, TAMs primarily accumulated within the stroma, while in wtp53, M1 TAMs were significantly more prevalent than M2 TAMs, irrespective of their location (*P* < 0.05, Student’s *t*-test) (Fig. 1C). The correlations between M1/M2 TAMs within islets and stroma differed between wtp53 and mutp53 tumors. Compared to mutp53, wtp53 tumor islets showed a higher correlation of M1 and M2 TAMs (*r* = 0.73 vs. 0.38, *P* < 0.001 and *P* < 0.001) (Fig. 1D, Supplementary Fig. 1D, E), and stromal M2 TAMs were also highly correlated with islet M2 TAMs in wtp53 tumor (*r* = 0.67 vs. 0.19, *P* < 0.0001 and *P* > 0.05) (Supplementary Fig. 1F, G). These findings reveal the spatial significance of M1 TAMs in cancer progression and the differential correlations of spatial M1/M2 TAMs between wtp53 and mutp53 lung adenocarcinomas.

**Table 1:** The distribution of M1/M2 tumor-associated macrophage in wild-type or mutant EGFR and TP53 lung adenocarcinoma.

		EGFR			TP53		
		wt	mutant	*p*-value	wt	mutant	*p*-value
	*N* (%)	24	93		33	84	
M1							
Islets				0.07			0.02*
	Low	19 (79)	55 (59)		22 (67)	72 (86)	
	High	5 (21)	38 (41)		11 (33)	12 (14)	
Stroma				0.42			0.38
	Low	12 (50)	55 (59)		21 (64)	46 (55)	
	High	12 (50)	38 (41)		12 (36)	38 (45)	
M2							
Islets				0.11			0.03*
	Low	20 (83)	80 (86)		24 (73)	76 (90)	
	High	4 (17)	13 (14)		9 (27)	8 (10)	
Stroma				0.31			0.24
	Low	15 (63)	68 (73)		26 (79)	57 (68)	
	High	9 (37)	25 (27)		7 (21)	27 (32)	

The cell count is calculated by the positive cell number divided the indicated tissue area (mm^2^). Low: 0–100; High: >100 cells per mm^2^.

*Chi-square test, *p* < 0.05.

### TP53 and M1/M2 tumor-associated macrophages determine the overall survival

Previous studies emphasize the significance of both the spatial distribution and specific subtypes of TAMs as key factors influencing lung cancer progression [4–6]. Based on this, the ratio of islet to stromal TAMs reflects the balance of spatial distribution, while the comparison between M1/M2 ratios indicates the balance of specific TAM subtypes. Therefore, we first estimated the ratio of islet to stromal TAMs in 117 lung adenocarcinomas [7, 8]. A high M1 ratio correlated with improved survival in lung adenocarcinoma, while the M2 ratio showed no such association (M1, *P* < 0.001; M2, *P* = 0.459, log-rank test) (Supplementary Fig. 1H, I). Considering the significance of TAM localization, the spatial balance of TAMs was further compared. Tumors in which the ratio of M1 TAMs is higher than that of M2 TAMs are defined as M1-dominant tumors, and vice versa for M2-dominant tumors. The results revealed that patients with M1-dominant tumors had better overall survival (Fig. 1E). Specifically, in the wtp53 group, patients exhibited significantly prolonged survival when their tumors had dominant M1 TAMs (Fig. 1F). However, the survival benefit of M1 TAMs disappeared in the mutp53 group (Fig. 1G). Analysis of the TCGA-LUAD database showed that M1-signature, which was developed in our previous research, was significantly associated with survival in wtp53 patients, but not in mutp53 (Fig. 1H) [19]. Overall, M1 TAMs were positively associated with the survival of lung adenocarcinoma with wtp53, especially when M1 TAMs were predominantly expressed within the tumor islets.

### The M1 macrophages induce cell apoptosis through wtp53

**Fig. 2:**
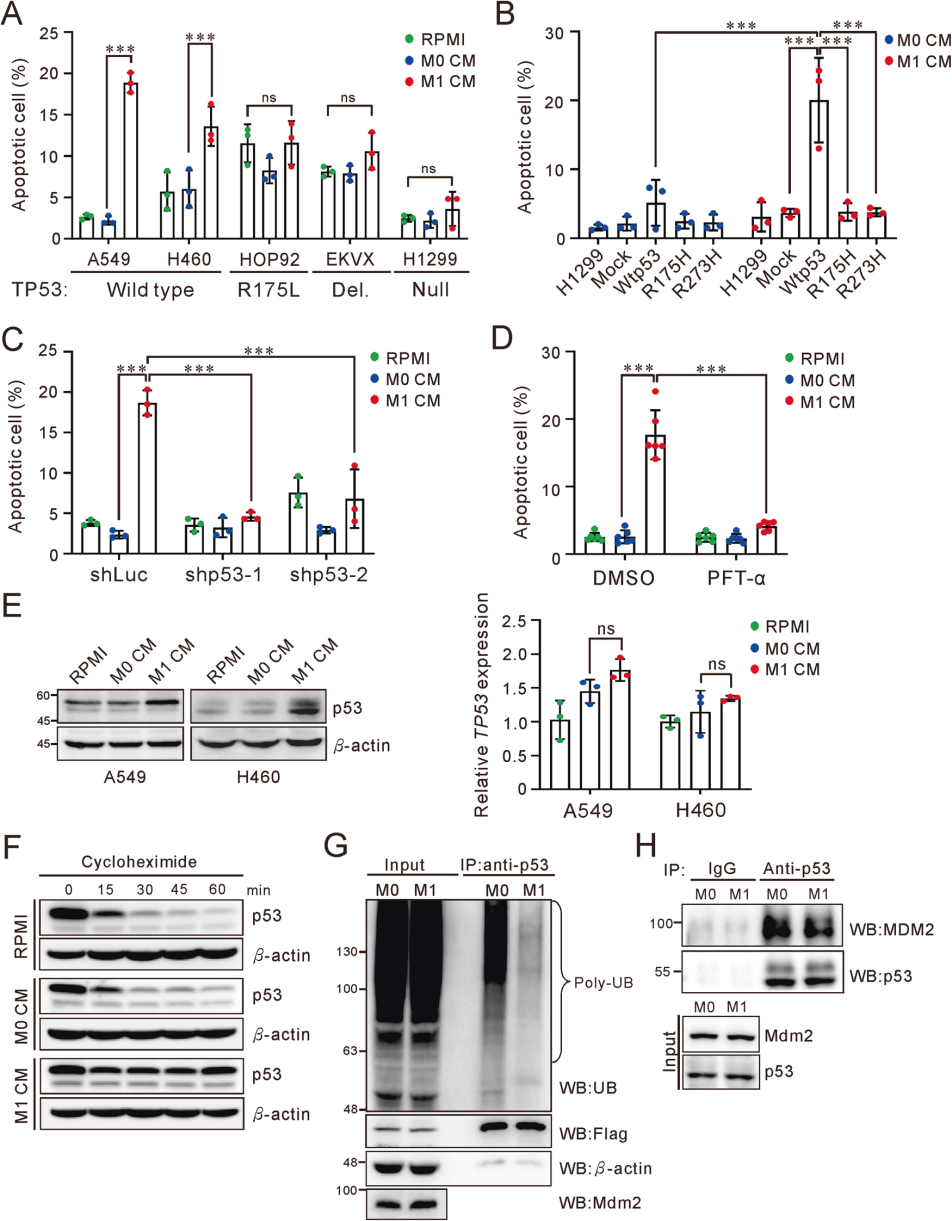
M1 macrophages promote cell apoptosis and enhance p53 accumulation. **A** Cell apoptosis in lung cancer cell lines cultured in RPMI, M0 and M1 CM. The apoptotic cell was quantified using Annexin V staining and assessed by flow cytometry. *P*-value determined by one-way ANOVA with Tukey’s post hoc test. **B** Cell apoptosis in H1299 cells cultured in M0 and M1 CM. Cells were transfected with mock, wtp53, p53-R175H and p53-R273H constructs and incubated in CMs for 3 days. *P*-value determined by two-way ANOVA with Tukey’s post hoc test. **C** Silencing p53 reduced M1-induced apoptosis in A549 cells. shLuc served as a negative control. *P*-value determined by two-way ANOVA with Tukey’s post hoc test. **D** Cell apoptosis in A549 cells treated with or without p53 inhibitor. Cells were treated with 30 μM PFT-α in RPMI, M0 and M1 CM for 3 days. PFT-α: pifithrin-α. *P*-value determined by two-way ANOVA with Tukey’s post hoc test. **E** The p53 expression at protein and mRNA levels in A549 and H460 cells. Cells were incubated in CMs for 3 days, assessed by immunoblot (left) and qRT-PCR (right). *P*-value determined by one-way ANOVA with Tukey’s post hoc test. **F** The p53 protein stability in A549 cells after treatment with RPMI, M0, and M1 CM. CM cultured-cells were treated with 100 μg/ml cycloheximide and harvested as indicated times. **G** Ubiquitination of p53 in H1299 cells after culture in M0 and M1 CM. Cells overexpressing Flag-wtp53, MDM2 and Ub were cultured in CM for 30 h and treated with 30 μM MG132 for 4 h prior to harvest. Lysates were immunoprecipitated with anti-p53 (FL393) antibody and analyzed by immunoblot. **H** The interaction of p53 and MDM2 in A549 cells after M0 and M1 CM treatment. Cells were cultured with M0 and M1 CM for 72 h, treated with 30 μM MG132 for 4 h prior to harvest. Lysates were immunoprecipitated with anti-p53 (DO-1) antibody and analyzed by immunoblot. Data are representative of at least two independent experiments and represented as mean ± SD. ns not significant, *P*-value > 0.05; *P-value < 0.05; **P-value < 0.01; ***P-value < 0.001. See Supplementary Fig. 2A for gating strategy of apoptosis assay.

Consistent with the clinical finding and our previous research, the enriched pathways of M1-treated A549 cells were related to p53 and cell apoptosis (Fig. 1I and Supplementary Table 3) [19]. Next, we examined the apoptotic response of various cancer cell lines in the conditioned medium from M1 macrophages (M1 CM), which polarized by the monocytic cell line, THP-1. Remarkably, M1 CM induced apoptosis in over 20% of the cells in A549 and H460 lines, which harbor wild-type p53. In contrast, cell lines with mutant p53 or lacking p53 exhibited no or mild apoptotic response to M1 CM, as well as colon cancer cells (Fig. 2A, Supplementary Fig. 2A and 2B). H1299 cells, which originally lack p53, underwent apoptosis only when wtp53 was ectopically introduced. Once cells overexpressing mutant p53 variants (p53-R175H and R273H), they failed to respond to M1 CM (Fig. 2B and Supplementary Fig. 2C). Knocking down wtp53 or pharmacologically inhibiting it with pifithrin-α (PFT-α) in A549 cells inhibited M1 CM-induced apoptosis, confirming the necessity of wtp53 activity (Fig. 2C, D and Supplementary Fig. 2D). Interestingly, PFT-α did not decrease p53 protein levels upon M1 CM treatment, suggesting that M1 CM might promotes p53 stability and activity rather than its transcript expression (Supplementary Fig. 2E). Indeed, M1 CM up-regulated the protein level of p53 in A549 and H460 cells without elevating *TP53* expression (Fig. 2E). Treatment with cycloheximide, which blocks new protein synthesis, and MG132, a proteasome inhibitor, showed that M1 CM extended the half-life of p53 protein and reduced its polyubiquitination (Fig. 2F, G). M1 CM also reduced the interaction between p53 and MDM2, the primary E3 ubiquitin ligase for p53 (Fig. 2H). These results indicate that M1 macrophages regulate p53 stability, emphasizing the critical role of p53 in tumor suppressive function of M1 macrophages.

### Synergetic effect of IFN-γ and IFN-β on cell apoptosis

**Fig. 3:**
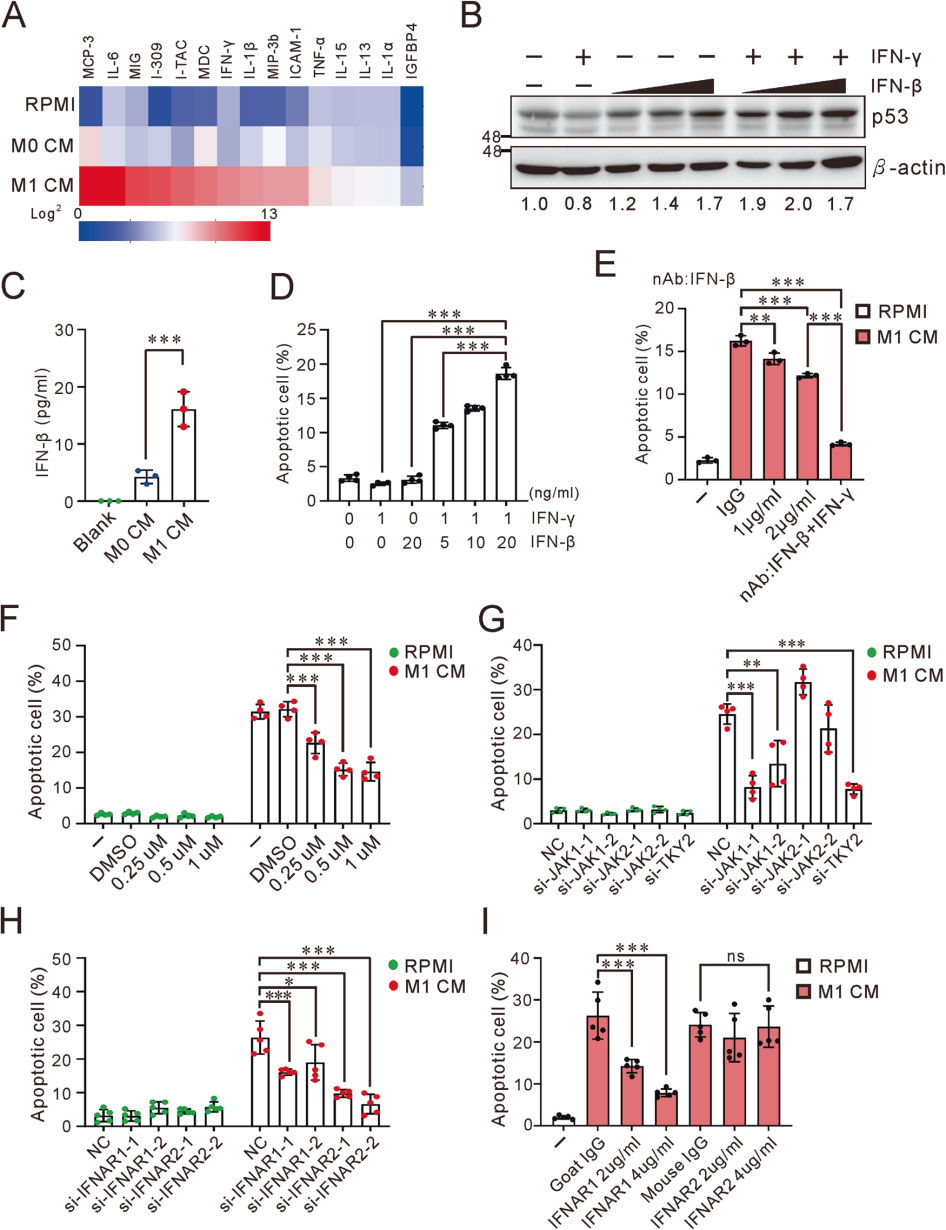
M1-induced apoptosis via the IFN-β signaling pathway. **A** Cytokine profiling of M0 and M1 CM. High-glucose RPMI supplied with 10% FBS is served as the medium control. **B** Effect of IFN-β, IFN-γ or combination treatments on p53 expression in A549 cells. Cells were treated with 1 ng/ml IFN-γ and/or 5, 10, and 20 ng/ml IFN-β for 3 days. The lower panel shows p53 protein levels normalized to β-actin, quantified by ImageJ. **C** IFN-β levels in M0 and M1 CM measured by ELISA. High-glucose RPMI supplied with 10% FBS is served as the medium control. **D** Synergistic effect of IFN-β and IFN-γ on apoptosis in A549 cells. Cells were treated with IFNs at indicated concentrations for 3 days. **E** Inhibition of M1-induced apoptosis through IFN-β neutralization. The combination treatment included 2 μg/ml each of IFN-β and IFN-γ neutralizing antibodies (nAb) and 4 μg/ml IgG as the negative control. **F** Effect of JAK inhibitor I on M1-induced apoptosis in A549 cells. DMSO served as the negative control. **G** Effect of JAK1, JAK2, and TYK2 silencing on M1-induced apoptosis in A549 cells. NC: siRNA negative control. **H** Effect of IFNAR1 and IFNAR2 silencing on M1-induced apoptosis in A549 cells. NC: siRNA negative control. **I** Inhibition of M1-induced apoptosis through IFNAR1 and IFNAR2 neutralization. M1 CM was supplied with neutralizing antibodies or 2 μg/ml IgG. Apoptotic cells were quantified using Annexin V staining and assessed by flow cytometry. Data are representative of at least two independent experiments and represented as mean ± SD. *P*-value for multiple group comparisons determined by two-way ANOVA, otherwise using one-way ANOVA and followed by Tukey’s post hoc test. ns not significant, *P*-value > 0.05; *P-value < 0.05; **P-value < 0.01; ***P-value < 0.001. See Supplementary Fig. 2A for gating strategy of apoptosis assay.

To identify the factor(s) in M1 CM that triggers apoptosis, we conducted a cytokine array encompassing 274 cytokines and performed transcriptome analysis of M1 macrophages. The cytokine array result revealed a rich presence of chemokines and pro-inflammatory cytokines, such as, IL-6, CXCL9 (MIG), IFN-γ, IL-1β and TNF-α in M1 CM (Fig. 3A). IFN-γ is known for its antitumor functions and is predominantly produced by T cells. IFN-γ production from M1 macrophages was also verified (Supplementary Fig. 3A, B). Unexpectedly, neutralizing IFN-γ in M1 CM slightly reduce M1-induced apoptosis and p53 (Supplementary Fig. 3C, D). Treating A549 cells with IFN-γ alone slightly reduced p53 at protein level (Fig. 3B and Supplementary Fig. 3E). Investigation through whole transcriptome profiling of M1 macrophages highlighted IFN-β expression, one of the type I interferon members, which was subsequently verified via real-time PCR and ELISA (Fig. 3C, and Supplementary Fig. 3F). Our findings demonstrate that a non-lethal dose of IFN-γ combined with IFN-β effectively induced p53 accumulation and cell apoptosis (Fig. 3B, D and Supplementary Fig. 3E). Additionally, neutralizing both IFN-γ and IFN-β was able to decrease cell apoptosis to below 5% following M1 CM treatment (Fig. 3E). This evidence uncovered the nuanced role of IFN-γ, in concert with IFN-β, demonstrating the multiple factors M1 macrophages deploy for the sophisticated immunoregulatory mechanisms.

Further, the inhibition of JAK, alongside the silencing of JAK1 and TYK2, significantly reduced M1-induced apoptosis, whereas silencing JAK2 had no influence (Fig. 3F, G and Supplementary Fig. 3G). These observations highlight the predominant influence of IFN-β signaling in this context. Silencing the IFN-β receptors, IFNAR1 and IFNAR2, attenuated the apoptosis elicited by M1 CM (Fig. 3H and Supplementary Fig. 3H). Interestingly, neutralizing IFNAR2 failed to mitigate the apoptosis, suggesting its specific role in intrinsic signal transduction (Fig. 3I). Together, these results underscore the crucial signaling of the IFNs/IFNAR/JAK1 axis in promoting the accumulation of p53, thereby leading to apoptosis.

### STAT1 interacted with p53 to enhance its accumulation through Y701 phosphorylation

**Fig. 4:**
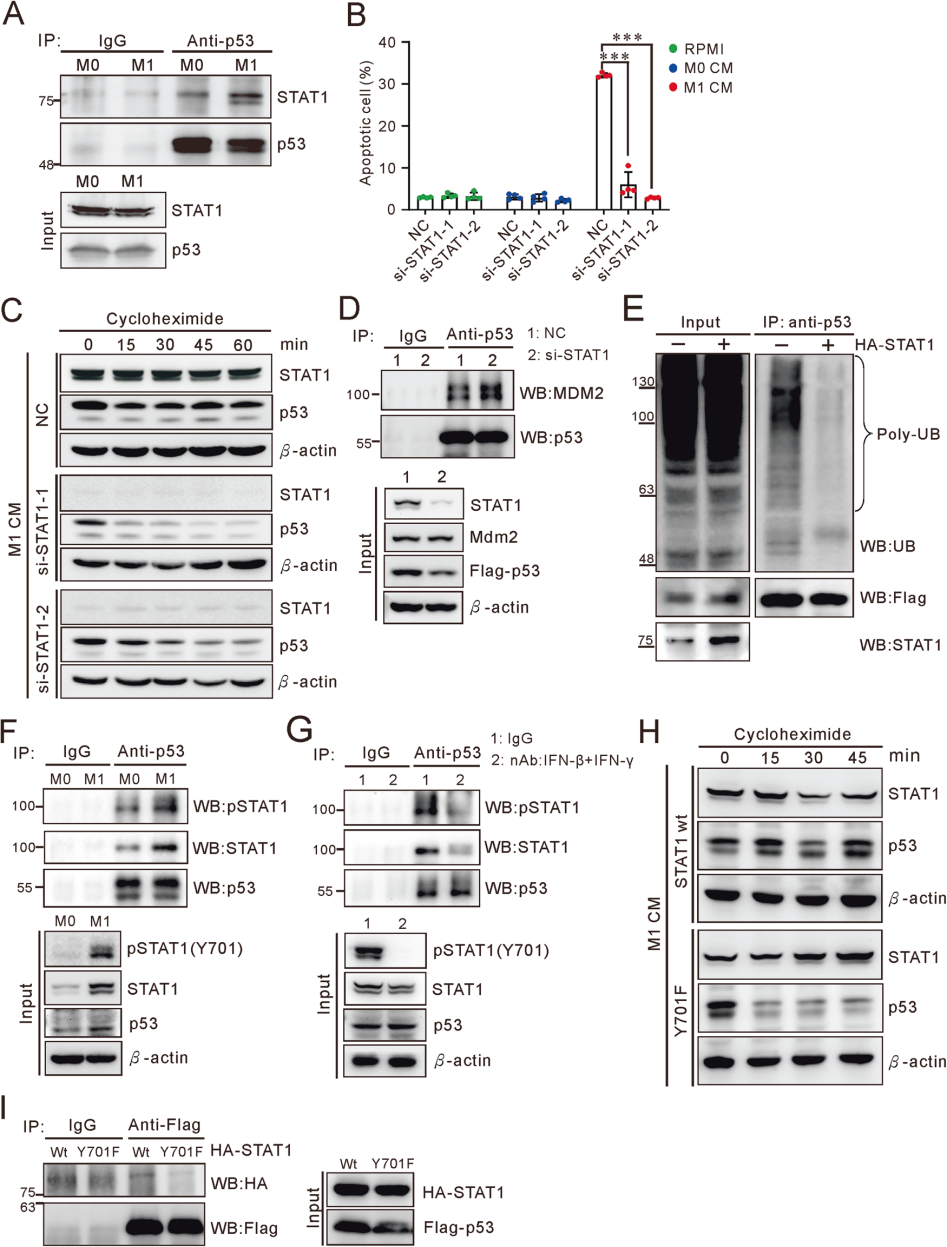
STAT1 enhances p53 stability via phosphorylation at Y701. **A** Interaction of p53 and STAT1 in M1 CM. Immunoprecipitation of p53 using anti-p53 antibody (FL-393) and immunoblot using anti-STAT1 and anti-p53 (DO-1) antibodies. Cell lysate was prepared from A549 cells treated with M0 or M1 CM for 3 days. **B** Effect of STAT1 silencing on M1-induced apoptosis in A549 cells. NC: siRNA negative control. Data are represented as mean ± SD; *n* = 4. *P*-value determined by two-way ANOVA with Tukey’s post hoc test. See Supplementary Fig. 2A for gating strategy of apoptosis assay. **C** Effect of STAT1 silencing on p53 protein stability in A549 cells. The M1-culture cells were treated with 100 μg/ml cycloheximide and harvested as indicated duration. NC siRNA negative control. **D** The interaction of p53 and MDM2 in STAT1 knockdown cells. si-STAT1 was delivered into H1299 cells, followed by transfection with p53 and MDM2. After culture in M1 CM for 30 h, cells were treated with 30 μM MG132 for 4 h prior to harvest. Lysates were immunoprecipitated with anti-p53 (DO-1) antibody and analyzed by immunoblot. **E** Ubiquitination of p53 in H1299 cells overexpressing STAT1. Cells transfected with Flag-wtp53, STAT1, and Ub were cultured in M0 or M1 CM for 30 h, followed by treatment with 30 μM MG132 for 4 h before harvesting. Immunoprecipitation was performed with an anti-p53 antibody (GTX102965) and analyzed by immunoblot. **F** The interaction of p53 and phosphorylated STAT1 in M0 and M1 CM treated A549 cells. Cells were culture in M0 and M1 CM for 72 h and then harvested. Lysates were immunoprecipitated with anti-p53 (DO-1) antibody and analyzed by immunoblot. The phosphorylation of STAT1 was assayed by anti-pY701-STAT1 (58D6). **G** Interaction between p53 and STAT1 in A549 cells treated with M1 CM and neutralizing antibodies against IFN-γ and IFN-β. Cells were culture M1 CM with IgG control or the combination of IFN-γ and IFN-β neutralizing antibodies (nAb) for 48 h. Prior to harvest, cells were treated with 30 μM MG132 for 4 h. Lysates were immunoprecipitated with anti-p53 (DO-1) antibody and analyzed by immunoblot. The combination treatment included 1 μg/ml each of IFN-β and IFN-γ nAbs and 2 μg/ml IgG as the negative control. **H** Interaction of p53 with either STAT1 or Y701F mutant. HEK293 cells co-transfected with Flag-p53 and either HA-STAT1 or HA-STAT1-Y701F vectors were immunoprecipitated with anti-Flag antibody and assayed by immunoblot with anti-Flag and anti-HA antibodies. I Effect of a dominant negative STAT1-Y701F mutant on the p53 protein stability. H1299 cells, expressing either STAT1 or STAT1-Y701F, were treated with M1 CM and then with 100 μg/ml cycloheximide, and samples were collected at indicated durations. Data are representative of at least two independent experiments.

As a key transcription factor in type I and type II interferon signaling, STAT1 also engages in interactions with p53 through its C-terminal domain [20]. Immunoprecipitation assays showed that M1 CM strengthens the interaction between p53 and STAT1 (Fig. 4A and Supplementary Fig. 4A). We found that M1 CM transcriptionally increased STAT1 expression, and also enhanced the expression of ectopic p53 in H1299 cells (Supplementary Fig. 4B–D). To elucidate the relationship of STAT1 and p53, we manipulated STAT1. Silencing STAT1 not only interrupted M1-induced apoptosis but also reduced p53 expression (Fig. 4B and Supplementary Fig. 4E). Moreover, depletion of STAT1 reverted the extended half-life of p53 induced by M1 CM and increase the interaction of p53 and MDM2 in H1299 and A549 cells (Fig. 2F, 4C, D and Supplementary Fig. 4F). Overexpression of STAT1 led to a decrease in the poly-ubiquitination of p53 and enhanced the apoptosis of A549 cells (Fig. 4E and Supplementary Fig. 4G). The results demonstrate that STAT1 is critical in the regulation of p53 protein accumulation and M1-induced apoptosis. This hypothesis posits that STAT1 activation, prompted by IFNs/IFNAR/JAK1 signaling, facilitates its interaction with p53. Indeed, M1 CM induced STAT1-Y701 phosphorylation and enhanced p53 interacting with STAT1 (Fig. 4F and Supplementary Fig. 4H). Furthermore, neutralizing IFN-γ and IFN-β in M1 CM decreased STAT1-Y701 phosphorylation and the interaction between STAT1 and p53 (Fig. 4G). Introducing the phosphorylation-deficient mutant, STAT1-Y701F, revealed a diminished interaction with p53 and a consequent reduction in p53 half-life, emphasizing the significance of STAT1 phosphorylation in regulating p53 accumulation (Fig. 4H, I and Supplementary Fig. 4I). Collectively, STAT1 mediates p53 accumulation through direct interaction which is assisted by IFN activation.

### M1 CM reduced mutant p53 mRNA via IFN-β suppressing WIG1

Interestingly, M1 CM reduced both protein and mRNA levels of p53 in CL1-0 and H1975 lung cancer cell lines, which harbor R248W and R273H mutations, respectively (Supplementary Fig. 5A, B). We found that p53 mRNA stability decreased in M1 CM compared to M0 and RPMI controls (Supplementary Fig. 5C). WIG1, an RNA-binding protein, stabilizes p53 mRNA by binding to its 3’UTR and is also a p53 target gene [21]. WIG1 expression was also decreased in M1 CM and IFN-β treatment, corresponding with reduced mutp53 (Supplementary Fig. 5D–G) [22]. Notably, M0 CM has lower concentration of IFN-β and the data show the minor effect on WIG1 and mutp53 expression (Fig. 3C and Supplementary Fig. 5A–D). Neutralizing IFN-β in M1 CM reversed the reduction of mutp53 and WIG1 (Supplementary Fig. 5H). Additionally, WIG1 knockdown in H1975 cells led to a decrease in p53 mRNA expression (Supplementary Fig. 5I). The reduction in WIG1 is not only driven by IFN-β inhibition but also by the fact that, unlike wild-type p53, mutp53 cannot regulate the expression of its downstream target, WIG1. Therefore, upon the IFNs stimulation, the expressions of wtp53 and mutp53 were different.

### Influence of p53 on M1 macrophage antitumor effects and TLR agonist response

**Fig. 5:**
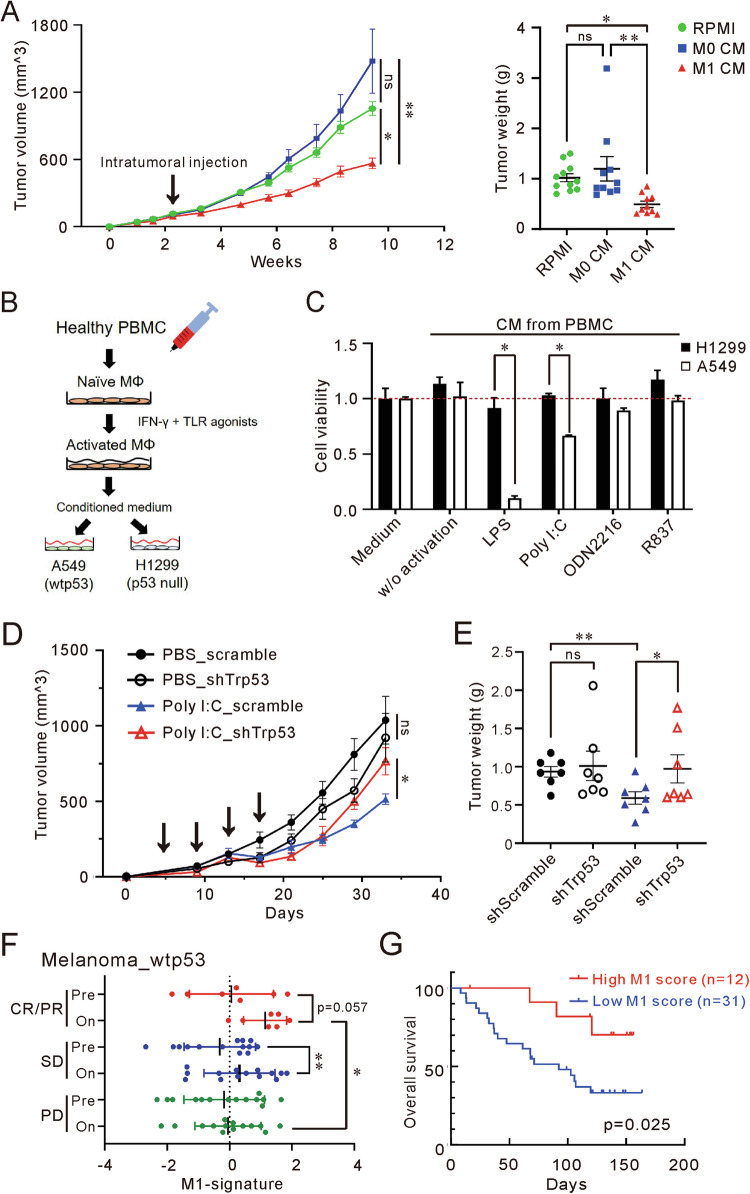
The therapeutic efficacy of M1 macrophages is p53-dependent. **A** Tumorigenesis of A549 cells in RPMI, M0 or M1 CM treatments. SCID mice were subcutaneously inoculated with A549 cells and intratumorally injected with RPMI, M0, or M1 CM twice weekly. The terminal tumor weights are measured and displayed on the right panel. Data are represented as mean ± SEM; RPMI group, *n* = 11; M0 and M1 groups, *n* = 10. *P*-value determined by one-way ANOVA with Tukey’s post hoc test. **B** The process of stimulating PBMCs into M1 macrophages using TLR agonists. **C** Inhibition of A549 and H1299 cell viability by TLR agonists polarized PBMCs. PBMCs were primed with IFN-γ and treated with LPS, poly I:C, ODN2216, or R837 for 24 h. The M1 macrophages derived from polarized PBMCs were cultured for an additional 24 h to prepare various CM. A549 and H1299 cells were cultured in fresh complete medium or CM for 5 days, and the live cells were counted excluding those stained with Trypan Blue. Data are represented as mean ± SD; *n* = 3. *P*-value determined by Student’s *t*-test. **D**, **E** Antitumor effects of Poly I:C on shScramble or shTP53-expressing CMT64 cells in vivo. Cells were injected subcutaneously into C57BL/6 mice and intravenous administration of 100 μL PBS or 50 μg poly I:C per mouse occurred on days 5, 9, 13, and 17. Data are represented as mean ± SEM; *n* = 7. *P*-value determined by paired and unpaired Student’s *t*-test. **F** An increased M1-signature associated with a better response to nivolumab in melanoma. The scores calculated for both pre-treatment and on-therapy samples. Data are mean ± SD. *P*-value determined by paired Student’s *t*-test and Wilcoxon two-sample test. **G** Kaplan–Meier analysis of overall survival in advanced melanoma patients based on the on-therapy M1-signature scores. *P*-value for overall survival was determined by log-rank test. ns not significant, *P*-value > 0.05; *P-value < 0.05; **P-value < 0.01; ***P-value < 0.001.

To assess the therapeutic effect of M1 CM in vivo, we intratumorally injected either RPMI, M0 CM, or M1 CM into SCID mice bearing A549 tumors. M1 CM significantly reduced tumor growth compared to both RPMI and M0 CM treatments (Fig. 5A, and Supplementary Fig. 6A, B).

To evaluate the selectively tumoricidal effect of M1 macrophages, we choose TLR agonists to differentiate human peripheral blood mononuclear cells (PBMCs), including poly I:C (TLR3 agonist), LPS (TLR4 agonist), CpG ODN2216 (TLR9 agonist), and R837 (TLR7 agonist), which are known to promote M1 macrophage polarization (Fig. 5B) [23, 24]. Consistent with expectations, LPS-derived M1 macrophage selectively reduced the viability of A549 cells but not H1299 cells. Additionally, Poly I:C exhibited a similar selective inhibition (Fig. 5C). To determine if p53 status influences the antitumor effect of poly I:C in an immunocompetent setting, we inoculated C57BL/6 mice with CMT64 cells expressing shRNA targeting murine trp53 (Supplementary Fig. 6C). No significant difference between scrambled and shTrp53 tumors in growth when treated with PBS. However, poly I:C reduced tumor growth in scramble but failed to suppress tumor growth in shTrp53 (Fig. 5D, E and Supplementary Fig. 6D, E). Overall, these results emphasize p53 modulates the therapeutic effect of M1 macrophages, specifically those differentiated with poly I:C.

### M1-signature score augmented by immune checkpoint inhibitor and associated with outcome

To assess the involvement of M1 macrophages in the efficacy of immune checkpoint inhibitor (ICI) treatments, we used public databases to estimate M1-signature score. Given the lack of specific ICI datasets for lung cancer, we turned to pre- and on-therapy melanoma transcriptomic and WES data (GSE91061 and SRP095809). We filtered out four cases with p53 missense mutations and those without WES data (Supplementary Table 4) [25, 26]. Following nivolumab administration, patients exhibiting complete or partial responses (CR/PR), as well as those with stable disease (SD), showed higher M1-signature scores, in contrast to those with progressive disease (PD). Notably, tumors responding better to ICIs demonstrated significantly higher M1-signature scores in on-therapy samples (CR/PR vs. PD, *P* = 0.013) (Fig. 5F). A higher M1-signature score was significantly associated with improved survival (Fig. 5G and Supplementary Tables 5, 6). Collectively, the evidence showed a positive correlation between higher M1-signature scores and favorable outcomes in ICI treatment, suggesting that high infiltration or polarization of M1 macrophages may improve the ICI response.

## Discussion

EGFR and TP53 are two of the most commonly mutated genes in lung adenocarcinoma. Our investigation reveals a discernible role for TP53 in the spatial orchestration of M1/M2 TAMs distribution. Contrarily, EGFR mutations exhibit a comparatively minimal effect on the presence of M1/M2 TAMs. However, inhibiting EGFR markedly diminish the prevalence of M2 TAMs and regulatory T cells, intimating that a strategic reprogramming of TAMs from an M2 to an M1 phenotype might serve as a viable approach to overcome the resistance to EGFR tyrosine kinase inhibitors (TKIs) [11, 27]. The complex relationship between EGFR TKIs, TAMs warrants further investigation. Stromal M1 TAMs are not only enriched in lymph node metastases [5], but also positively associated with advanced cancer stages, poor cellular differentiation, and smoking history in lung adenocarcinoma (Fig. 1B and Supplementary Table 1), suggesting that stromal M1 TAMs profoundly influences the cancer progression. The pronounced correlation between the densities of M1 and M2 TAMs within the tumor islets of wtp53 tumors suggests the capacity of functional p53 to augment inflammatory cytokine production, thereby enhancing macrophage recruitment (Supplementary Fig. 1D, correlation coefficient = 0.73). Nonetheless, the correlation of M2 TAMs within tumor islets and stroma in wtp53 tumors is notably stronger than that of M1 TAMs (Fig. 1D, correlation coefficient, M1 vs M2 = 0.11 vs 0.67), driving the hypothesis that wtp53 tumors may also preferentially promote the polarization of antitumor M1 macrophages at the tumor core [13]. Loss of p53 increases macrophage infiltration and promotes M2 macrophage polarization [28, 29]. However, stimulating p53 expression reprograms macrophage polarization from the M2 phenotype to the M1 phenotype [30]. CSF1 expression is significantly upregulated in mutp53 lung adenocarcinomas (Supplementary Fig. 1B) and in a p53-R172H–dependent manner in metastatic lung lesions of esophageal squamous cell carcinoma [31]. This evidence suggests that p53 status in tumor cells plays a critical role in mediating macrophage polarization and infiltration.

The restoration of p53 can trigger cellular senescence, apoptosis, and tumor regression in vivo [32, 33], mirroring the effects observed when M1 macrophages, polarized in vitro, induce cell senescence and apoptosis (Fig. 2A, B) [19]. Indeed, genes regulated by M1 macrophages show significant enrichment in apoptosis and p53 signaling pathways. No prognostic benefit of an M1-signature or M1 dominance in lung adenocarcinomas with mutant p53 were revealed. Considering the protumorigenic properties of M2 macrophages, we noted an up-expressed islet M2 TAM in wtp53 tumors, and an enrichment of stromal M2 TAMs in stage II and III tumors (Table 1 and Supplementary Table 1). However, median survival for M2-dominant tumors did not differ between wtp53 and mutp53 patients (median survival for wtp53: 3.5 years, 95% CI, 2.0–6.6; for mutp53: 5.2 years, 95% CI, 3.2–6.3; log-rank test, *P* > 0.05) (Supplementary Fig. 7). While the prognostic impact of M1/M2 TAM distribution is complex and linked to genetic alterations, within the context of p53, it is predominantly influenced by M1 TAMs.

IFN-γ, the sole member of type II interferon, is a cytotoxic cytokine and signals via the heterodimer receptor, IFNGR1 and IFNGR2. The downstream kinases JAK1 and JAK2 sequentially phosphorylate STAT1 [34]. However, JAK2 is not required in M1-induced apoptosis and neutralizing IFN-γ slightly reduces cell apoptosis. Apparently, the apoptotic signaling of IFN-γ is not mediated by the IFNGR1/2 heterodimer in the M1 context. Synergistic function of IFN-γ and IFN-β in enhancing p53 expression and cell apoptosis implies that IFN-γ or the IFN-γ-IFNGR complex may be involved in the binding of IFN-β and IFNAR1/2, thereby improving the signaling. IFNAR1 is able to physically interact with IFNGR2 in a non-ligand-binding manner or from a specific IFN-β-IFNAR1 complex independent of IFNAR2 [35–37]. Neutralizing IFNAR2 failed to reduce M1-induced apoptosis; on the contrary, silencing IFNAR2 and blocking IFNAR1 completely inhibits cell apoptosis, but silencing IFNAR1 marginally decreases apoptosis. Consistent with a previous study, IFNAR1 initially receives the extrinsic signals engaging with TYK2, whereas IFNAR2 is crucial for intrinsic signal transduction that is required for STAT1 binding and activation, which is crucial for subsequent gene induction [38].

*STAT1* expression in breast cancer was associated with poor prognosis and CD68-positive macrophage infiltration. However, NSCLC patients with higher *STAT1* have a lower hazard of death [39, 40]. STAT1-Y701 phosphorylation is significantly associated with favorable overall survival and relapse-free survival [39]. The dominant-negative mutation, STAT1-Y701F, decreases the interaction with p53 and reduces M1-prolonged p53 stability. Moreover, STAT1-Y701 phosphorylation induces the formation of canonical pSTAT1 homodimers or pSTAT1/pSTAT2 heterodimers, which translocate to the nucleus where they bind to the interferon-stimulated response elements (ISREs) or a promoter of IFNγ-activated genes (GAGs) to promote the interferon-stimulated genes (ISGs) expression [41]. The synergistic interaction between Type I and Type II IFNs through their receptors and intracellular signaling, alongside the unclear process by which STAT1 selects its binding partner, either other phosphorylated STAT proteins or p53, is yet to be fully understood. The exposure duration and quantity of IFNs are presumably issues. Treatment with a high dose of IFN-γ triggers cell apoptosis via the STAT1/JAK/caspase signaling pathway, while a low dose of IFN-γ promotes tumor cell stemness [42]. Our study shows that M1-induced apoptosis is via a non-lethal dose of IFN-γ augmenting the effects of IFN-β. This likely offers a closer representation of the real immune response in tumor microenvironment, which is shaped by various and plenty of inflammatory factors released by M1 TAMs.

Polyinosinic:polycytidylic acid (poly I:C) treatment activates dendritic cells and macrophages, and induces IFN-β production in the tumor microenvironment [43]. Poly I:C is used to stimulate immune response in cancer vaccination as an adjuvant and has shown promising anti-tumor effects in murine models [23, 44]. Combining poly I:C with R838 treatments has led to tumor regression, marked by increased macrophage infiltration with a high M1 to M2 ratio, primarily through the activation of the STAT1 pathway [23]. Ongoing clinical trials are assessing the safety and efficacy of poly I:C-based treatments, including nanoplexed poly I:C (BO-112) and poly ICLC (Hiltonol), as monotherapies and in combination with radiotherapy, chemotherapy, or ICIs [45]. Notably, clinical trial NCT02828098 revealed that combining BO-112 with nivolumab or pembrolizumab could counteract primary resistance to anti-PD-1 therapies [44]. Post-treatment biopsies showed high expression levels of CCL19 (MIP-3b) and CXCL9 (MIG) in the responding patients [44]. Furthermore, we observed overexpression of these chemokines, along with CXCL10, in polarized M1 macrophages, underscoring their importance in ICI response [46]. The indispensable roles of TAMs and p53 in ICI efficacy have been established [3, 47, 48], with TP53 diminishing PD-L1 expression in tumor cells and targeting the promoter of DD1α to enhance macrophage-mediated clearance of dead cells [49, 50]. NSCLC patients with wtp53 tumors exhibit lower response rates and survival with ICIs compared to those with mutp53 [51, 52]. However, reactivating p53 increases tumor sensitivity to anti-PD-1 therapy [53]. We found responding melanomas with elevated M1-signature score, suggesting that combining M1 macrophage-polarizing agents like poly I:C with PD1/PD-L1 inhibitors may particularly benefit the patients with wtp53 tumors.

The study has certain limitations. First, in vitro polarization of M1/M2 macrophages may not fully capture TAM characteristics within the tumor microenvironment. Demonstrating the spatial relationship between M1/M2 macrophages and tumor cells in vitro is challenging. Second, the specific traits of M1/M2 TAMs in islet and stroma are still unclear. The influence of their location within the tumor microenvironment on cancer progression is particularly intriguing, as it dictates whether M1 TAMs promote or inhibit cancer. Advanced spatial methodologies are needed to better understand these location-specific interactions and the complex regulatory mechanisms of M1/M2 TAMs related to tumor cells.

In summary, the spatial distribution of M1/M2 TAMs serves as a marker for cancer progression and prognosis. Whether p53 is mutated or not emerges as a critical factor determining the equilibrium between M1 and M2 TAMs within the tumor islets and stroma. We revealed that the antitumor activity of M1 macrophages depends on the IFNs/STAT1/p53 signaling pathway in cancer cells. The STAT1 Y701 phosphorylation enhance the interaction of p53 and STAT1 and then decreased the interaction between p53 and MDM2. Thus, a higher presence of M1 TAMs correlates with improved survival rate in lung adenocarcinoma patients with wtp53, but this benefit does not extend to those with mutp53 tumors. These insights underline the importance of developing p53-specific companion diagnostics in conjunction with M1-oriented therapies.

## Material and methods

### Human lung tumor specimens and healthy individual’s PBMC

A total of 137 lung adenocarcinoma was enrolled who underwent complete surgical resection or biopsy at the Taichung Veterans General Hospital (Taichung, Taiwan) between February 2011 and October 2016. The study was reviewed and approved by Institutional Review Boards of Taichung Veterans General Hospital (No. C08197). The study of peripheral blood mononuclear cells (PBMCs) from healthy individuals was reviewed and approved by the Institutional Review Boards of National Taiwan University Hospital (No. 201812059RINA). All participants provided written informed consent and all procedures were performed in accordance with the relevant guidelines and regulations.

### MALDI-TOF MS

Mutations T790M, L858R, and Del19 in the EGFR gene were detected using Matrix-Assisted Laser Desorption Ionization–Time of Flight Mass Spectrometry (MALDI-TOF MS), following the manual of the MassARRAY system (Agena Bioscience, San Diego, CA). The customized primers, probes and the procedure for analyzing these mutations were previously described [54].

### Deep sequencing of multiplex polymerase chain reaction (PCR) amplicons

Multiplex PCR was conducted to amplify 40 ng of genomic DNA using the custom QIAseq Targeted DNA Panel (QIAGEN, Hilden, Germany), targeting specific regions within the EGFR and TP53 genes, according to the manufacturer’s instructions. This kit targets 82 amplicons located in the exon regions of the EGFR and TP53 genes. For target sequencing, up to 48 samples were pooled and sequenced on an Illumina NextSeq500 instrument using the 300 cycle Kit (Illumina San Diego, CA). Sequence mapping and variant calling were executed using the pipeline provided by QIAGEN. Single nucleotide variants (SNV), stop gain mutations, frameshift insertions and deletions were annotated by ANNOVAR. Any alteration in p53 identified by Cosmic 70 and ClinVar (RRID:SCR_006169) was considered a mutation unless it was annotated as benign. Mutations identified through DNA mass spectrometry-based gene testing were regarded as additional EGFR mutations, serving as supplementary information. Supplementary annotations include nonframeshift insertions and the L591R mutation among four cases. The data are provided in Supplementary Table 7.

### IHC staining and evaluation

For immunohistochemical (IHC) analysis, 5 μm sections of formalin-fixed, paraffin-embedded tissue were subjected to pretreatment with ULTRA Cell Conditioning Solution 1, pH 8.5 (Ventana Medical Systems Inc., Tucson, AZ, USA) to facilitate heat-induced epitope retrieval. Staining was performed by a Ventana BenchMark stainer (Ventana Medical Systems Inc.) with the following primary antibodies: CD68 (clone KP-1, Roche Diagnostics), CD163 (clone 10D6, ThermoFisher), and HLA-DR (clone TAL 1B5, dilution 1:50, Santa Cruz Biotechnology), following the manufacturers’ protocol. To quantify M1/M2 macrophage, sections underwent double staining with M1 markers (CD68 and HLA-DR) and, in separate series, with M2 markers (CD68 and CD163). A pathologist blindly scored the double-positive cells in the tumor islet and stroma for each case, quantifying the cell density in mm^2^.

### M1-signature and GSEA analysis

The TCGA-LUAD database was accessed and downloaded using the TCGAbiolinks R package. Somatic mutation of TP53 for each case (tumor vs. adjacent normal lung tissue) was obtained from Aggregate GDC MAFs aligned with the hg38 reference genome. STAR-Counts files were downloaded for the quantification of gene expression. The data from Nivolumab-treated melanoma were acquired from the National Center for Biotechnology Information (NCBI) databases, under accession number GSE91061. The TP53 missense mutations annotated in a previous publication were included in the analysis [25]. FPKM values was converted to *z*-score to normalize gene expression data. These *z*-scores were then utilized to calculate the M1-signature score using the following formula: (0.29 × *z*-score of PALM2-AKAP2) + (−0.06 × *z*-score of GPX2) + (0.15 × *z*-score of LTBP4) + (−0.055 × *z*-score of FGB) + (−0.20 × *z*-score of HSPE1) + (0.27 × *z*-score of TNIP1) + (0.50 × *z*-score of HLA-C) + (0.39 × *z*-score of HLA-B) + (−0.11 × *z*-score of SCD) + (0.25 × *z*-score of C1QTNF1) [19]. The median of the scores was used as a cutoff for classifying patients. The transcriptomic profiles of A549 cells cocultured with M1 macrophages (M1-A549) and A549 cells alone (A549) were obtained from GSE50658 [19]. The gene profiles were applied to gene set enrichment analysis (GSEA) according to the default setting.

### Cell culture and macrophage polarization

Human lung cancer cell lines A549, EKVX, H1299, H460, HOP92 were obtained from the National Cancer Institute, Center for Cancer Research cell repository (Bethesda, MD). The human monocyte cell line THP-1 and its STR-PCR profiling were provided from Bioresource Collection and Research Center (BCRC, Taiwan). All human lung cancer cell lines, including H1299 cells and the embryonic kidney cell line HEK293T, were cultured in RPMI 1640 medium. The murine lung adenocarcinoma cell line CMT64 (ECACC 10032301), provided by MilliporeSigma, was grown in DMEM medium. Both medium were supplemented with 10% FBS (GIBCO-BRL/Thermo Fisher Scientific, Waltham, MA). The cell lines were regularly tested for mycoplasma contamination. The polarization of THP-1 cells into M0 and M1 macrophages and collection of conditioned medium (CM) have been previously described [19]. Briefly, THP-1 cells were differentiated into adherent M0 macrophages by culture in the supernatant from the phorbol-12-myristate-13-acetate (Sigma, St Louis, MO) inducing macrophages. The M0 macrophages were sequentially polarized into M1 macrophages via treatment with IFN-γ (20 ng/ml) and LPS (1 μg/ml) (Sigma). Fresh PBMCs were isolated by Ficoll-Paque PLUS (GE healthcare Life Science) following a standard protocol and then cultured in serum-free high-glucose RPMI medium supplemented with NEAA for 3 days. The adherent macrophages from 2 × 10^6^ monocytes were treated with 20 ng/ml IFN-γ for 16 h, followed by M1 polarization agents, 1 μg/ml LPS, 10 μg/ml ODN2216, 100 μg/ml poly I:C, and 10 μg/ml R837 (InvivoGen, San Diego, CA), for an additional 24 h. After a fresh medium replacement for 24 h, the supernatant was collected to treat A549 and H1299 cells for 3 days. Cell lines were routinely applied for mycoplasma testing using MycoAlert Detection Kit (Lonza).

### Cell apoptosis

Cell apoptosis was assessed by FC500 flow cytometry (Beckman Coulter, Indianapolis, IN) with the apoptosis detection kit (BD Pharmingen, San Diego, CA), following the manufacturer’s instructions. The percentage of apoptotic cells was quantified based on the proportion of Annexin V-positive cells. Every assay was performed in at least two independent experiments for reliability.

### Agents

Cells were exposed to M1 CM in presence of Pifithrin-α and JAK inhibitor I (SantaCruz Biotechnology, Dallas, TX) for 3 days or cultured in M1 CM for 30 h followed by the treatment of MG132 (Cell signaling, Danvers, MA) for 4 h. To assess protein stability, cells treated with M1 CM were subsequently incubated in serum-free medium containing 100 μg/ml Cycloheximide (Sigma-Aldrich). Recombinant human proteins, IFN-β (PeproTech EC, London, UK) and IFN-γ (R&D Systems, Minneapolis, MN), were administered to A549 cells in specified concentrations. Details of agents are listed in Supplementary Table 8.

### Vector construction, RNA silencing and quantitative real-time PCR

Wild-type p53, p53 mutants (R175H and R273H) and MDM2 were cloned into pcDNA3.1 vector (RRID:Addgene_79663) with a Flag tag. Wild-type STAT1, Y701F-STAT1 and Ubiquitin (Ub) were cloned into pcDNA3.1-HA vector (RRID:Addgene_128034). The siRNA sets of si-IFNAR1, si-IFNAR2, si-JAK1, si-STAT1 and si-TYK2 were purchased from Silencer® Select siRNA (Thermo Fisher Scientific). Cells were transfected with the vectors and siRNA using Lipofectamine^TM^ 2000 or RNAiMAX transfection reagent for 48 h and incubated with CM for 3 days. Lentiviruses expressing human Tp53, mouse Trp53, control shRNA and standard protocol were provided from the National RNAi Core Facility, Academia Sinica, Taiwan. The shRNA identifiers are as follows: shLuc: TRCN0000072243; human Tp53-1: TRCN0000003753; human Tp53-2: TRCN0000003756; shScramble: ASN0000000004; mouse Trp53: TRCN0000331289. For quantitative real-time PCR, the details are described in the Supplementary Methods.

### IFNAR1/2 neutralization, IFNs depletion and measurement

For the receptor neutralization, 5 × 10^4^ A549 cells were seeded in 24-well plates and pre-treated with goat polyclonal IFNAR1 (Abcam, Cambridge, UK) and mouse monoclonal IFNAR2 neutralizing antibodies (PBL Assay Science, Piscataway, NJ) for 4 h, followed by treatment with M1 CM for 3 days. For the cytokine depletion experiment, A549 cells were incubated in M1 CM pre-adsorbed by anti-human IFN-β (R&D Systems, Minneapolis, MN), anti-human IFN-γ (BD Pharmingen, San Diego, CA), and IgG control antibodies as indicated for 3 days, and then applied on apoptosis assay. For the enzyme-linked immunosorbent assay (ELISA), the concentrations of IFN-γ and IFN-β in M0, M1 CM and blank control (high-glucose RPMI containing 10% FBS) were measured using ELISA kits. Details of materials are listed in Supplementary Table 8.

### Immunoblot

Immunoblot and immunoprecipitation analyses were performed as methods described in our previous publication [55]. Cells were harvested and lysed in the M-PER™ Mammalian Protein Extraction Reagent (Thermo Fisher Scientific), supplemented with 1X Complete Protease Inhibitor Cocktail (Roche Diagnostics, Basel, Switzerland). Proteins were separated on SDS-PAGE gels and transferred onto PVDF membranes (Merck Millipore, Burlington, MA), which were probed with antibodies. Specified protein detection was performed by the Enhanced Chemiluminescence (ECL) kit (Millipore) and imaged with the FUJIFILM LAS-4000 ECL system (Fujifilm, Tokyo, Japan). The primary antibodies used for immunoblot included anti-p53, anti-STAT1, anti-phospho-Y701 STAT1, anti-MDM2, anti-HA, and anti-Ubiquitin. Monoclonal mouse anti-β-Actin antibody was used as a loading control. For co-immunoprecipitation, anti-p53 (FL-393 and GTX102965), ant-HA, anti-STAT1, and anti-Flag (CSB-MA000021M0m) were used. Details of primary antibodies are listed in Supplementary Table 8. Original and uncropped immunoblots were shown in Supplementary Material.

### Cytokine array

The Human Cytokine Array G4000, consisting of 274 antibodies, was obtained from RayBiotech (Norcross, GA). To minimize the interference of fetal bovine serum (FBS), M0 and M1 CM were collected using high-glucose RPMI medium supplemented with a low FBS concentration (2.5%). Fresh medium served as the blank control. All procedures and data normalization followed the manufacturer’s guidelines. Values below 10 were adjusted to 10. For the generation of the heatmap, values of M1 upregulated cytokines/proteins exhibiting a fold change greater than 2 (comparing M1 CM with RPMI and M1 with M0 CM), were subjected to log2 transformation.

### In vivo tumorigenesis assay

All mouse experiments were performed in accordance with the guidelines and regulations. Approvals were reviewed and obtained from Institutional Animal Care and Use Committee (IACUC) at National Taiwan University College of Medicine, and cared for at the Association for Assessment and Accreditation of Laboratory Animal Care International (AAALAC)-approved vivarium facilities. The approval number is 20180385 and 20210076. A549 cells were subcutaneously injected into the dorsal region of 6-week-old NOD-SCID female mice at 1 × 10^6^ cells per mouse. When the tumor reached an approximate size of 150 mm^3^, the mice were randomized into three group. Subsequently, 50 μl of RPMI, M0 or M1 CM were intratumorally administered twice weekly. To eliminate the FBS interference in CM, FBS was substituted with HL-1™ Supplement in the medium (Lonza, Basel, Switzerland). In the syngeneic mouse model, CMT64 cells expressing shScramble and shTrp53 were subcutaneously inoculated at 5 × 10^5^ cells per mouse into the left and right dorsal regions, respectively, of 6-week-old C57BL/6 female mice. The mice were intravenously administered with 100 μl of PBS or 50 μg of poly I:C on days 5, 9 13 and 17. Tumor size and body weight were measured twice a week. Any mouse with a body weight exceeding three standard deviations above or below the group mean will be excluded from the analysis. Tumor volume (V) was calculated by the formula: V = length (a) × width (b)^2^ / 2. Tumor weight was determined on the day the mice were sacrificed.

### Statistical analysis

The power analysis was performed to determine the appropriate sample size for detecting a statistically significant difference between the treatment and control groups. Setting the significance level (α) at 0.05 and the power (1 - β) at 0.8, a sample size per group was estimated for each mouse experiment. Results are presented as mean ± SD, except as specified. To compare differences between the two groups, an *F*-test was first performed to assess the homogeneity of variances. Differences between two groups were analyzed using a two-tailed Student’s *t*-test. Comparisons among multiple groups were conducted using one-way ANOVA followed by Tukey’s post hoc test for pairwise comparisons. Survival outcomes were evaluated using Kaplan–Meier survival analysis and compared with the log-rank test. The cutoff was defined by the median value, except as specified. *P-*value < 0.05 was considered statistically significant. Statistical analyses were performed with SPSS version 23 (IBM, Chicago, IL, RRID:SCR_002865) and GraphPad Prism version 9 (RRID:SCR_002798).

## Supplementary information

Supplementary Tables

Supplementary Figures

WB_Raw data

## Data Availability

The datasets generated during the current study are available from the corresponding author on reasonable request.

## References

[1] Gentles AJ, Newman AM, Liu CL, Bratman SV, Feng W, Kim D, et al. The prognostic landscape of genes and infiltrating immune cells across human cancers. Nat Med. 2015;21:938–45. 26193342 10.1038/nm.3909 PMC4852857

[2] Vanmeerbeek I, Govaerts J, Laureano RS, Sprooten J, Naulaerts S, Borras DM, et al. The interface of tumour-associated macrophages with dying cancer cells in immuno-oncology. Cells-Basel. 2022;11:3890. 10.3390/cells11233890 PMC9741298 36497148

[3] Mantovani A, Marchesi F, Malesci A, Laghi L, Allavena P. Tumour-associated macrophages as treatment targets in oncology. Nat Rev Clin Oncol. 2017;14:399–416. 28117416 10.1038/nrclinonc.2016.217 PMC5480600

[4] Ohri CM, Shikotra A, Green RH, Waller DA, Bradding P. Macrophages within NSCLC tumour islets are predominantly of a cytotoxic M1 phenotype associated with extended survival. Eur Respir J. 2009;33:118–26. 19118225 10.1183/09031936.00065708

[5] Jackute J, Zemaitis M, Pranys D, Sitkauskiene B, Miliauskas S, Vaitkiene S, et al. Distribution of M1 and M2 macrophages in tumor islets and stroma in relation to prognosis of non-small cell lung cancer. BMC Immunol. 2018;19:3. 29361917 10.1186/s12865-018-0241-4 PMC5781310

[6] Mei J, Xiao Z, Guo C, Pu Q, Ma L, Liu C, et al. Prognostic impact of tumor-associated macrophage infiltration in non-small cell lung cancer: a systemic review and meta-analysis. Oncotarget. 2016;7:34217–28. 27144518 10.18632/oncotarget.9079 PMC5085150

[7] Welsh TJ, Green RH, Richardson D, Waller DA, O’Byrne KJ, Bradding P. Macrophage and mast-cell invasion of tumor cell islets confers a marked survival advantage in non-small-cell lung cancer. J Clin Oncol. 2005;23:8959–67. 16219934 10.1200/JCO.2005.01.4910

[8] Feng PH, Yu CT, Wu CY, Lee MJ, Lee WH, Wang LS, et al. Tumor-associated macrophages in stage IIIA pN2 non-small cell lung cancer after neoadjuvant chemotherapy and surgery. Am J Transl Res. 2014;6:593–603. 25360223 PMC4212933

[9] Rannikko JH, Hollmen M. Clinical landscape of macrophage-reprogramming cancer immunotherapies. Br J Cancer. 2024;131:627–40. 38831013 10.1038/s41416-024-02715-6 PMC11333586

[10] Guaitoli G, Tiseo M, Di Maio M, Friboulet L, Facchinetti F. Immune checkpoint inhibitors in oncogene-addicted non-small cell lung cancer: a systematic review and meta-analysis. Transl Lung Cancer R. 2021;10:2890–916. 10.21037/tlcr-20-941 PMC8264334 34295687

[11] Wang XP, Semba T, Manyam GC, Wang J, Shao S, Bertucci F, et al. EGFR is a master switch between immunosuppressive and immunoactive tumor microenvironment in inflammatory breast cancer. Sci Adv. 2022;8:eabn7983 36525493 10.1126/sciadv.abn7983 PMC9757751

[12] Zheng Y, Hao S, Xiang C, Han Y, Shang Y, Zhen Q, et al. The correlation between SPP1 and immune escape of EGFR mutant lung adenocarcinoma was explored by bioinformatics analysis. Front Oncol. 2021;11:592854. 34178613 10.3389/fonc.2021.592854 PMC8222997

[13] Lujambio A, Akkari L, Simon J, Grace D, Tschaharganeh DF, Bolden JE, et al. Non-cell-autonomous tumor suppression by p53. Cell. 2013;153:449–60. 23562644 10.1016/j.cell.2013.03.020 PMC3702034

[14] Cooks T, Pateras IS, Jenkins LM, Patel KM, Robles AI, Morris J, et al. Mutant p53 cancers reprogram macrophages to tumor supporting macrophages via exosomal miR-1246. Nat Commun. 2018;9:771. 29472616 10.1038/s41467-018-03224-w PMC5823939

[15] Feldser DM, Kostova KK, Winslow MM, Taylor SE, Cashman C, Whittaker CA, et al. Stage-specific sensitivity to p53 restoration during lung cancer progression. Nature. 2010;468:572–5. 21107428 10.1038/nature09535 PMC3003305

[16] Murray PJ, Allen JE, Biswas SK, Fisher EA, Gilroy DW, Goerdt S, et al. Macrophage activation and polarization: nomenclature and experimental guidelines. Immunity. 2014;41:14–20. 25035950 10.1016/j.immuni.2014.06.008 PMC4123412

[17] Pyonteck SM, Akkari L, Schuhmacher AJ, Bowman RL, Sevenich L, Quail DF, et al. CSF-1R inhibition alters macrophage polarization and blocks glioma progression. Nat Med. 2013;19:1264–72. 24056773 10.1038/nm.3337 PMC3840724

[18] Geng K, Ma X, Jiang Z, Gu J, Huang W, Wang W, et al. WDR74 facilitates TGF-beta/Smad pathway activation to promote M2 macrophage polarization and diabetic foot ulcer wound healing in mice. Cell Biol Toxicol. 2023;39:1577–91. 35982296 10.1007/s10565-022-09748-8

[19] Yuan A, Hsiao YJ, Chen HY, Chen HW, Ho CC, Chen YY, et al. Opposite effects of M1 and M2 macrophage subtypes on lung cancer progression. Sci Rep. 2015;5:14273. 26399191 10.1038/srep14273 PMC4585843

[20] Townsend PA, Scarabelli TM, Davidson SM, Knight RA, Latchman DS, Stephanou A. STAT-1 interacts with p53 to enhance DNA damage-induced apoptosis. J Biol Chem. 2004;279:5811–20. 14602726 10.1074/jbc.M302637200

[21] Vilborg A, Glahder JA, Wilhelm MT, Bersani C, Corcoran M, Mahmoudi S, et al. The p53 target Wig-1 regulates p53 mRNA stability through an AU-rich element. Proc Natl Acad Sci USA. 2009;106:15756–61. 19805223 10.1073/pnas.0900862106 PMC2773521

[22] Madar S, Harel E, Goldstein I, Stein Y, Kogan-Sakin I, Kamer I, et al. Mutant p53 attenuates the anti-tumorigenic activity of fibroblasts-secreted interferon beta. PLoS One. 2013;8:e61353. 23630584 10.1371/journal.pone.0061353 PMC3632588

[23] Anfray C, Mainini F, Digifico E, Maeda A, Sironi M, Erreni M, et al. Intratumoral combination therapy with poly(I:C) and resiquimod synergistically triggers tumor-associated macrophages for effective systemic antitumoral immunity. J Immunother Cancer. 2021;9:e002408. 34531246 10.1136/jitc-2021-002408 PMC8449972

[24] Karapetyan L, Luke JJ, Davar D. Toll-like receptor 9 agonists in cancer. Oncotargets Ther. 2020;13:10039–60. 10.2147/OTT.S247050 PMC7553670 33116588

[25] Riaz N, Havel JJ, Makarov V, Desrichard A, Urba WJ, Sims JS, et al. Tumor and microenvironment evolution during immunotherapy with nivolumab. Cell. 2017;171:934–949 e916. 29033130 10.1016/j.cell.2017.09.028 PMC5685550

[26] Sun H, Liu SY, Zhou JY, Xu JT, Zhang HK, Yan HH, et al. Specific TP53 subtype as biomarker for immune checkpoint inhibitors in lung adenocarcinoma. Ebiomedicine. 2020;60:102990. 32927274 10.1016/j.ebiom.2020.102990 PMC7494676

[27] Peng H, Chen B, Huang W, Tang Y, Jiang Y, Zhang W, et al. Reprogramming tumor-associated macrophages to reverse EGFR(T790M) resistance by dual-targeting codelivery of gefitinib/vorinostat. Nano Lett. 2017;17:7684–90. 29160717 10.1021/acs.nanolett.7b03756

[28] Wellenstein MD, Coffelt SB, Duits DEM, van Miltenburg MH, Slagter M, de Rink I, et al. Loss of p53 triggers WNT-dependent systemic inflammation to drive breast cancer metastasis. Nature. 2019;572:538–42. 31367040 10.1038/s41586-019-1450-6 PMC6707815

[29] Blagih J, Zani F, Chakravarty P, Hennequart M, Pilley S, Hobor S, et al. Cancer-specific loss of p53 leads to a modulation of myeloid and T cell responses. Cell Rep. 2020;30:481–96.e486. 31940491 10.1016/j.celrep.2019.12.028 PMC6963783

[30] Cheng H, Fan X, Ye E, Chen H, Yang J, Ke L, et al. Dual tumor microenvironment remodeling by glucose-contained radical copolymer for MRI-guided photoimmunotherapy. Adv Mater. 2022;34:e2107674. 34755922 10.1002/adma.202107674

[31] Efe G, Dunbar KJ, Sugiura K, Cunningham K, Carcamo S, Karaiskos S, et al. p53 gain-of-function mutation induces metastasis via BRD4-dependent CSF-1 expression. Cancer Discov. 2023;13:2632–51. 37676642 10.1158/2159-8290.CD-23-0601 PMC10841313

[32] Xue W, Zender L, Miething C, Dickins RA, Hernando E, Krizhanovsky V, et al. Senescence and tumour clearance is triggered by p53 restoration in murine liver carcinomas. Nature. 2007;445:656–60. 17251933 10.1038/nature05529 PMC4601097

[33] Ventura A, Kirsch DG, McLaughlin ME, Tuveson DA, Grimm J, Lintault L, et al. Restoration of p53 function leads to tumour regression in vivo. Nature. 2007;445:661–5. 17251932 10.1038/nature05541

[34] Hauptstein N, Meinel L, Luhmann T. Bioconjugation strategies and clinical implications of Interferon-bioconjugates. Eur J Pharm Biopharm. 2022;172:157–67. 35149191 10.1016/j.ejpb.2022.02.006

[35] de Weerd NA, Vivian JP, Nguyen TK, Mangan NE, Gould JA, Braniff SJ, et al. Structural basis of a unique interferon-beta signaling axis mediated via the receptor IFNAR1. Nat Immunol. 2013;14:901–7. 23872679 10.1038/ni.2667

[36] Takaoka A, Mitani Y, Suemori H, Sato M, Yokochi T, Noguchi S, et al. Cross talk between interferon-gamma and -alpha/beta signaling components in caveolar membrane domains. Science. 2000;288:2357–60. 10875919 10.1126/science.288.5475.2357

[37] Lasfar A, Cook JR, Cohen Solal KA, Reuhl K, Kotenko SV, Langer JA, et al. Critical role of the endogenous interferon ligand-receptors in type I and type II interferons response. Immunology. 2014;142:442–52. 24597649 10.1111/imm.12273 PMC4080960

[38] Shemesh M, Lochte S, Piehler J, Schreiber G. IFNAR1 and IFNAR2 play distinct roles in initiating type I interferon-induced JAK-STAT signaling and activating STATs. Sci Signal. 2021;14:eabe4627. 34813358 10.1126/scisignal.abe4627

[39] Tymoszuk P, Charoentong P, Hackl H, Spilka R, Muller-Holzner E, Trajanoski Z, et al. High STAT1 mRNA levels but not its tyrosine phosphorylation are associated with macrophage infiltration and bad prognosis in breast cancer. BMC Cancer. 2014;14:257. 24725474 10.1186/1471-2407-14-257 PMC4021106

[40] Chen HY, Yu SL, Chen CH, Chang GC, Chen CY, Yuan A, et al. A five-gene signature and clinical outcome in non-small-cell lung cancer. New Engl J Med. 2007;356:11–20. 17202451 10.1056/NEJMoa060096

[41] Philips RL, Wang Y, Cheon H, Kanno Y, Gadina M, Sartorelli V, et al. The JAK-STAT pathway at 30: much learned, much more to do. Cell. 2022;185:3857–76. 36240739 10.1016/j.cell.2022.09.023 PMC9815833

[42] Song M, Ping Y, Zhang K, Yang L, Li F, Zhang C, et al. Low-dose IFNgamma induces tumor cell stemness in tumor microenvironment of non-small cell lung cancer. Cancer Res. 2019;79:3737–48. 31085700 10.1158/0008-5472.CAN-19-0596

[43] Muller E, Speth M, Christopoulos PF, Lunde A, Avdagic A, Oynebraten I, et al. Both Type I and Type II interferons can activate antitumor M1 macrophages when combined with TLR stimulation. Front Immunol. 2018;9:2520. 30450098 10.3389/fimmu.2018.02520 PMC6224375

[44] Marquez-Rodas I, Longo F, Rodriguez-Ruiz ME, Calles A, Ponce S, Jove M, et al. Intratumoral nanoplexed poly I:C BO-112 in combination with systemic anti-PD-1 for patients with anti-PD-1-refractory tumors. Sci Transl Med. 2020;12. 10.1126/scitranslmed.abb0391 33055241

[45] De Waele J, Verhezen T, van der Heijden S, Berneman ZN, Peeters M, Lardon F, et al. A systematic review on poly(I:C) and poly-ICLC in glioblastoma: adjuvants coordinating the unlocking of immunotherapy. J Exp Clin Canc Res. 2021;40:213. 10.1186/s13046-021-02017-2 PMC8229304 34172082

[46] House IG, Savas P, Lai JY, Chen AXY, Oliver AJ, Teo ZL, et al. Macrophage-derived CXCL9 and CXCL10 are required for antitumor immune responses following immune checkpoint blockade. Clin Cancer Res. 2020;26:487–504. 31636098 10.1158/1078-0432.CCR-19-1868

[47] Munoz-Fontela C, Mandinova A, Aaronson SA, Lee SW. Emerging roles of p53 and other tumour-suppressor genes in immune regulation. Nat Rev Immunol. 2016;16:741–50. 27667712 10.1038/nri.2016.99 PMC5325695

[48] Gordon SR, Aute RLM, Dulken BW, Hutter G, George BM, Ccracken MNM, et al. PD-1 expression by tumour-associated macrophages inhibits phagocytosis and tumour immunity. Nature. 2017;545:495. 28514441 10.1038/nature22396 PMC5931375

[49] Cortez MA, Ivan C, Valdecanas D, Wang XH, Peltier HJ, Ye YP, et al. PDL1 regulation by p53 via miR-34. J Natl Cancer Inst. 2015;108:djv303. 26577528 10.1093/jnci/djv303 PMC4862407

[50] Yoon KW, Byun S, Kwon E, Hwang SY, Chu KK, Hiraki M, et al. Control of signaling-mediated clearance of apoptotic cells by the tumor suppressor p53. Science. 2015;349:1261669. 10.1126/science.1261669 PMC5215039 26228159

[51] Hellmann MD, Nathanson T, Rizvi H, Creelan BC, Sanchez-Vega F, Ahuja A, et al. Genomic features of response to combination immunotherapy in patients with advanced non-small-cell lung cancer. Cancer Cell. 2018;33:843. 29657128 10.1016/j.ccell.2018.03.018 PMC5953836

[52] Dong ZY, Zhong WZ, Zhang XC, Su J, Xie Z, Liu SY, et al. Potential predictive value of TP53 and KRAS mutation status for response to PD-1 blockade immunotherapy in lung adenocarcinoma. Clin Cancer Res. 2017;23:3012–24. 28039262 10.1158/1078-0432.CCR-16-2554

[53] Yang ZF, Ka-Li Sun J, Lee MM, Chan MK. Restoration of p53 activity via intracellular protein delivery sensitizes triple negative breast cancer to anti-PD-1 immunotherapy. J Immunother Cancer. 2022;10:e005068. 36104100 10.1136/jitc-2022-005068 PMC9476161

[54] Su KY, Chen HY, Li KC, Kuo ML, Yang JC, Chan WK, et al. Pretreatment epidermal growth factor receptor (EGFR) T790M mutation predicts shorter EGFR tyrosine kinase inhibitor response duration in patients with non-small-cell lung cancer. J Clin Oncol. 2012;30:433–40. 22215752 10.1200/JCO.2011.38.3224

[55] Hsu CY, Chang GC, Chen YJ, Hsu YC, Hsiao YJ, Su KY, et al. FAM198B is associated with prolonged survival and inhibits metastasis in lung adenocarcinoma via blockage of ERK-mediated MMP-1 expression. Clin Cancer Res. 2018;24:916–26. 29217529 10.1158/1078-0432.CCR-17-1347

