# Supplementary material for: Tp53 determines the spatial dynamics of M1/M2 tumor-associated macrophages and M1-driven tumoricidal effects
Source: Cell Death Dis. 2025 Jan 22;16(1):38. doi: 10.1038/s41419-025-07346-0 (PMC11754596; doi:10.1038/s41419-025-07346-0)
Supplement: Supplementary file 1 — Supplementary Tables [file 41419_2025_7346_MOESM1_ESM.docx]

**Supplementary Table 1. The density of spatial M1/M2 TAMs in clinicopathologic characteristics.**

|  |  | Islet M1 | | Stroma M1 | | Islet M2 | | Stroma M2 | |
| --- | --- | --- | --- | --- | --- | --- | --- | --- | --- |
|  | n | Cell/mm^2^ | *P* | Cell/mm^2^ | *P* | Cell/mm^2^ | *P* | Cell/mm^2^ | *P* |
| **Age** |  |  |  |  |  |  |  |  |  |
| ≥60 | 59 | 77.3 | 0.211 | 110.2 | 0.898 | 69.8 | 0.265 | 72.3 | 0.081 |
| <60 | 58 | 58.4 |  | 112.4 |  | 49.0 |  | 104.8 |  |
| **Gender** |  |  |  |  |  |  |  |  |  |
| Female | 64 | 57.4 | 0.129 | 114.1 | 0.716 | 60.5 | 0.923 | 77.9 | 0.240 |
| Male | 53 | 81.5 |  | 107.8 |  | 58.7 |  | 101.0 |  |
| **Smoking** |  |  |  |  |  |  |  |  |  |
| Never | 84 | 61.8 | 0.250 | 97.6 | 0.025^✽^ | 59.5 | 0.978 | 88.8 | 0.916 |
| Smoker | 33 | 84.3 |  | 146.2 |  | 60.1 |  | 86.6 |  |
| **Stage** |  |  |  |  |  |  |  |  |  |
| I | 46 | 66.2 | 0.205 | 67.8 | <0.001^✽^ | 62.8 | 0.825 | 66.1 | 0.001^✽^ |
| II | 14 | 105.9 |  | 137.1 |  | 82.1 |  | 114.2 |  |
| III | 39 | 67.3 |  | 127.1 |  | 61.1 |  | 129.7 |  |
| IV | 18 | 44.1 |  | 165.5 |  | 28.7 |  | 33.1 |  |
| **Differentiation** |  |  |  |  |  |  |  |  |  |
| Well to mod. | 5 | 81.4 | 0.512 | 42.2 | 0.004^✽^ | 38.6 | 0.971 | 61.6 | 0.611 |
| Moderate | 55 | 56.0 |  | 91.0 |  | 61.6 |  | 95.6 |  |
| Mod. to poor | 38 | 79.4 |  | 120.8 |  | 58.8 |  | 73.7 |  |
| Poor | 19 | 77.3 |  | 169.1 |  | 61.4 |  | 102.4 |  |
| **EGFR** |  |  |  |  |  |  |  |  |  |
| Wt | 24 | 73.9 | 0.893 | 101.3 | 0.182 | 64.8 | 0.965 | 98.3 | 0.200 |
| Mut | 93 | 67.6 |  | 105.4 |  | 59.9 |  | 78.0 |  |
| **TP53** |  |  |  |  |  |  |  |  |  |
| Wt | 33 | 92.0 | 0.102 | 105.6 | 0.680 | 67.8 | 0.588 | 61.5 | 0.070 |
| Mut | 84 | 58.8 |  | 113.5 |  | 56.5 |  | 98.6 |  |

Comparison of the means between two groups was conducted using Student's t-test; otherwise, one-way ANOVA was employed.

*: p value < 0.05

**Supplementary Table 2.** **Clinical characteristics and p53 mutation status of 117 lung adenocarcinomas.**

| Characteristic |  | Patient No. |  |  |  |
| --- | --- | --- | --- | --- | --- |
|  |  | p53 wt | p53 mutant | Total | p-value |
| N |  | 33 | 84 | 117 |  |
| Age |  | 61.1 | 60.8 | 60.9 |  |
|  | ≥60 | 18 | 41 | 59 | 0.68 |
|  | <60 | 15 | 43 | 58 |  |
| Gender |  |  |  |  |  |
|  | Female | 20 | 44 | 64 | 0.54 |
|  | Male | 13 | 40 | 53 |  |
| Smoking |  |  |  |  |  |
|  | Never | 26 | 58 | 84 | 0.36 |
|  | Smoker | 7 | 26 | 33 |  |
| Stage |  |  |  |  |  |
|  | I-II | 18 | 42 | 60 | 0.69 |
|  | III-IV | 15 | 42 | 57 |  |
| EGFR |  |  |  |  |  |
|  | wt | 8 | 16 | 24 | 0.61 |
|  | mutation | 25 | 68 | 93 |  |
| Differentiated^✽^ |  |  |  |  |  |
|  | well to moderately | 2 | 3 | 5 | 0.64 |
|  | moderately | 13 | 42 | 55 |  |
|  | mod to poorly | 11 | 27 | 38 |  |
|  | poorly | 7 | 12 | 19 |  |

✽:Chi-square test, otherwise using Fisher’s test.

**Supplementary Table 3. Gene set enrichment analysis of M1-altered genes in cell apoptosis and p53 signatures.**

| **Signatures** | **ES** | **NES** | **Nominal**  **p-value** | **FDR**  **q-value**^＊^ |
| --- | --- | --- | --- | --- |
| **Apoptosis-related signature** |  |  |  |  |
| Dutta_apoptosis_via_NFKB [1] | 0.62 | 1.61 | 0.019 | 0.045 |
| Biocarta_apoptosis_ pathway | 0.73 | 1.49 | 0.059 | 0.089 |
| KEGG_apoptosis | 0.45 | 1.44 | 0.024 | 0.092 |
| **TP53-related signature** |  |  |  |  |
| Kanna_TP53_targets_up [2] | 0.57 | 1.68 | 0.004 | 0.036 |
| Pid_p53_downstream_pathway [3] | 0.49 | 1.65 | 0.000 | 0.034 |
| KEGG_p53_signaling_pathway | 0.47 | 1.46 | 0.024 | 0.103 |

^＊^For generate hypotheses to drive further research, Gene Set Enrichment Analysis (GSEA) highlights the enrichment gene sets with an FDR of less than 25% which can be considered. ES, Enrichment Score; NES, Normalized Enrichment Score; FDR, False Discovery Rate.

**References**

1. Dutta J, Fan Y, Gupta N, Fan G, Gelinas C. Current insights into the regulation of programmed cell death by NF-kappaB. Oncogene. 2006;25(51):6800-16. doi: 10.1038/sj.onc.1209938. PubMed PMID: 17072329.

2. Kannan K, Amariglio N, Rechavi G, Jakob-Hirsch J, Kela I, Kaminski N, et al. DNA microarrays identification of primary and secondary target genes regulated by p53. Oncogene. 2001;20(18):2225-34. doi: 10.1038/sj.onc.1204319. PubMed PMID: 11402317.

3. Schaefer CF, Anthony K, Krupa S, Buchoff J, Day M, Hannay T, et al. PID: the Pathway Interaction Database. Nucleic Acids Res. 2009;37(Database issue):D674-9. doi: 10.1093/nar/gkn653. PubMed PMID: 18832364; PubMed Central PMCID: PMCPMC2686461.

**Supplementary Table 4. Four patients with TP53 missense mutations.**

| **Patient** | **Hugo Symbol** | **Variant Classification** | **HGVS_c** | **HGVS_p** |
| --- | --- | --- | --- | --- |
| Pt5 | TP53 | Missense_variant&splice_region_variant | c.374C>A | p.Thr125Lys |
| Pt23 | TP53 | Missense_variant | c.308A>G | p.Tyr103Cys |
| Pt25 | TP53 | Protein_protein_contact | c.832C>T | p.Pro278Ser |
| Pt49 | TP53 | Missense_variant | c.79C>A | p.Pro27Thr |

**Supplementary Table 5. Clinical Characteristics for high and low M1 score groups.**

|  |  | *N* | High M1 score (n=12) | Low M1 score (n=31) | *p-value |
| --- | --- | --- | --- | --- | --- |
| Response |  |  |  |  |  |
|  | PD | 17 | 2 | 15 | 0.058 |
|  | SD | 17 | 5 | 12 |  |
|  | PR | 6 | 4 | 2 |  |
|  | CR | 3 | 1 | 2 |  |
| Mutational subtype | |  |  |  |  |
|  | BRAF | 13 | 5 | 8 | 0.741 |
|  | NF1 | 3 | 0 | 3 |  |
|  | RAS | 7 | 2 | 5 |  |
|  | Triple wt | 20 | 5 | 15 |  |
| Subtype |  |  |  |  | 0.867 |
|  | Acral | 1 | 0 | 1 |  |
|  | Cutaneous | 29 | 9 | 20 |  |
|  | Mucosal | 5 | 2 | 3 |  |
|  | Ocular/uveal | 3 | 0 | 3 |  |
|  | Other | 5 | 1 | 4 |  |

✽: Fisher’s test.

**Supplementary Table 6. Clinical Characteristics for 43 patients.**

| Patient ID | Response | Subtype | Mutational  Subtype | M Stage | Time (Days) | Dead/Alive (Dead = True) |
| --- | --- | --- | --- | --- | --- | --- |
| Pt10 | SD | CUTANEOUS | NF1 | M1A | 36.5714 | TRUE |
| Pt101 | PR | CUTANEOUS | TripleWt | M1A | 119.143 | FALSE |
| Pt103 | PD | CUTANEOUS | TripleWt | M1B | 69.1429 | FALSE |
| Pt106 | PD | MUCOSAL | BRAF | M1C | 13 | TRUE |
| Pt108 | SD | OTHER | TripleWt | M1B | 130.429 | FALSE |
| Pt11 | PD | CUTANEOUS | BRAF | NA | 119.571 | TRUE |
| Pt13 | PD | CUTANEOUS | BRAF | NA | 40 | TRUE |
| Pt17 | PD | CUTANEOUS | TripleWt | M1C | 8.14286 | TRUE |
| Pt18 | PR | CUTANEOUS | BRAF | M1A | 153.286 | FALSE |
| Pt26 | SD | MUCOSAL | RAS | M1B | 136 | FALSE |
| Pt27 | PD | CUTANEOUS | BRAF | M1C | 67.8571 | TRUE |
| Pt28 | PD | CUTANEOUS | TripleWt | NA | 105.714 | TRUE |
| Pt3 | PR | CUTANEOUS | RAS | UNKNOWN | 163.429 | FALSE |
| Pt30 | CR | MUCOSAL | TripleWt | M1A | 150.429 | FALSE |
| Pt31 | PD | OTHER | TripleWt | M1A | 137.286 | FALSE |
| Pt32 | PD | CUTANEOUS | BRAF | M1C | 16.2857 | FALSE |
| Pt34 | PR | CUTANEOUS | BRAF | M1B | 119.143 | FALSE |
| Pt36 | SD | CUTANEOUS | TripleWt | M1C | 154.429 | FALSE |
| Pt37 | SD | CUTANEOUS | RAS | M1A | 92.2857 | TRUE |
| Pt38 | SD | CUTANEOUS | TripleWt | M1C | 23.8571 | TRUE |
| Pt4 | SD | CUTANEOUS | RAS | M1B | 90.4286 | TRUE |
| Pt44 | PR | MUCOSAL | BRAF | NA | 156.143 | FALSE |
| Pt46 | PD | OTHER | BRAF | M1A | 32.4286 | TRUE |
| Pt47 | PD | CUTANEOUS | BRAF | M1B | 102.571 | TRUE |
| Pt48 | CR | CUTANEOUS | TripleWt | M1C | 149.429 | FALSE |
| Pt52 | PD | CUTANEOUS | TripleWt | M1C | 68 | TRUE |
| Pt58 | SD | CUTANEOUS | NF1 | M1C | 71.2857 | TRUE |
| Pt65 | SD | ACRAL | TripleWt | M1B | 98.8571 | FALSE |
| Pt67 | SD | OCULAR/UVEAL | TripleWt | M1C | 147.429 | FALSE |
| Pt68 | PR | CUTANEOUS | TripleWt | M1B | 120.286 | FALSE |
| Pt77 | SD | CUTANEOUS | BRAF | M1A | 67.2857 | TRUE |
| Pt79 | SD | OTHER | NF1 | UNKNOWN | 31.1429 | TRUE |
| Pt8 | PD | MUCOSAL | TripleWt | M0 | 37 | TRUE |
| Pt82 | SD | OCULAR/UVEAL | TripleWt | M1C | 61.2857 | TRUE |
| Pt84 | PD | OTHER | TripleWt | M1C | 21.5714 | TRUE |
| Pt85 | PD | CUTANEOUS | RAS | M1A | 129.143 | FALSE |
| Pt87 | SD | CUTANEOUS | RAS | UNKNOWN | 139.143 | FALSE |
| Pt89 | SD | CUTANEOUS | TripleWt | M1A | 120.571 | TRUE |
| Pt9 | PD | CUTANEOUS | BRAF | M1C | 13.1429 | TRUE |
| Pt92 | SD | CUTANEOUS | RAS | UNKNOWN | 47.5714 | TRUE |
| Pt93 | PD | CUTANEOUS | TripleWt | M1C | 121.286 | FALSE |
| Pt94 | CR | CUTANEOUS | BRAF | M1C | 140.143 | FALSE |
| Pt98 | SD | OCULAR/UVEAL | TripleWt | M1C | 106.714 | TRUE |

**Supplementary Table 7. List of EGFR statuses derived from DNA sequencing and mass spectrometry results.**

| Pateint No. | EGFR status |
| --- | --- |
| P1 | del19 |
| P2 | L858R |
| P3 | L858R |
| P4 | del19 |
| P5 | del19 |
| P6 | L858R |
| P7 | L858R |
| P8 | **nonframeshift insertion** |
| P9 | L858R |
| P10 | del19 |
| P11 | del19 |
| P12 | L858R |
| P13 | del19 |
| P14 | L858R |
| P15 | **nonframeshift insertion** |
| P16 | L858R |
| P17 | del19 |
| P18 | L858R |
| P19 | unfound |
| P20 | del19 |
| P21 | unfound |
| P22 | G719S |
| P23 | del19 |
| P24 | unfound |
| P25 | del19 |
| P26 | L858R |
| P27 | G719A |
| P28 | unfound |
| P29 | unfound |
| P30 | del19 |
| P31 | unfound |
| P32 | del19 |
| P33 | G719C |
| P34 | L858R |
| P35 | del19 |

(Continued on following page)

| P36 | unfound |
| --- | --- |
| P37 | unfound |
| P38 | unfound |
| P39 | L858R |
| P40 | L858R |
| P41 | L858R |
| P42 | del19 |
| P43 | del19 |
| P44 | L858R |
| P45 | L858R |
| P46 | del19 |
| P47 | unfound |
| P48 | unfound |
| P49 | L858R |
| P50 | unfound |
| P51 | unfound |
| P52 | **L591R** |
| P53 | T790M/del19 |
| P54 | L861Q |
| P55 | L858R |
| P56 | del19 |
| P57 | L858R |
| P58 | G719S |
| P59 | L858R |
| P60 | unfound |
| P61 | L861Q |
| P62 | L858R/del19 |
| P63 | unfound |
| P64 | del19 |
| P65 | L858R |
| P66 | **L591R** |
| P67 | L858R |
| P68 | L858R |
| P69 | del19 |
| P70 | unfound |
| P71 | L858R |
| P72 | L858R |
| P73 | unfound |
| P74 | unfound |

(Continued on following page)

| P75 | L858R |
| --- | --- |
| P76 | del19 |
| P77 | L858R |
| P78 | unfound |
| P79 | unfound |
| P80 | L858R |
| P81 | del19 |
| P82 | L858R |
| P83 | E719C/del19 |
| P84 | unfound |
| P85 | del19 |
| P86 | unfound |
| P87 | del19 |
| P88 | L858R |
| P89 | del19 |
| P90 | G719S/E709A |
| P91 | L858R/E709A |
| P92 | unfound |
| P93 | L858R |
| P94 | del19 |
| P95 | L858R/T790M |
| P96 | unfound |
| P97 | L858R |
| P98 | del19 |
| P99 | del19 |
| P100 | del19 |
| P101 | del19 |
| P102 | L858R |
| P103 | del19 |
| P104 | G719S/S768I |
| P105 | L861Q |
| P106 | del19 |
| P107 | L858R |
| P108 | del19 |
| P109 | del19 |
| P110 | del19 |
| P111 | L858R |
| P112 | del19 |
| P113 | L858R |

(Continued on following page)

| P114 | L861Q |
| --- | --- |
| P115 | L858R |
| P116 | del19 |
| P117 | L858R |

The bold letter indicates that the mutation was identified by DNA mass spectrometry.

**Supplementary Table 8. Details of antibodies, commercial kits, other reagents and primer sequences.**

|  | **Reagent table** | **Source** | **Identifier** |
| --- | --- | --- | --- |
| **Cell lines** |  |  |  |
| Human monocyte | THP-1 | Bioresource Collection and Research Center | ATCC TIB-202 |
| Human lung cancer | A549 | National Cancer Institute, Center for Cancer Research cell repository | NCI-60 |
|  | EKVX | National Cancer Institute, Center for Cancer Research cell repository | NCI-60 |
|  | H1299 | National Cancer Institute, Center for Cancer Research cell repository | NCI-60 |
|  | H460 | National Cancer Institute, Center for Cancer Research cell repository | NCI-60 |
|  | HOP92 | National Cancer Institute, Center for Cancer Research cell repository | NCI-60 |
| Human conlon cancer | HCT116 | National Cancer Institute, Center for Cancer Research cell repository | NCI-60 |
|  | HT29 | National Cancer Institute, Center for Cancer Research cell repository | NCI-60 |
| Murin lung cancer | CMT64 | MilliporeSigma | ECACC 10032301 |
| **Antibodies** |  |  |  |
| IHC | CONFIRM anti-CD68 (KP-1) | Roche Diagnostics | 5278252001 |
|  | Anti-CD163 (10D6) | ThermoFisher | MA5-11458 |
|  | Anti-HLA-DR (TAL 1B5) | Santa Cruz Biotechnology | sc-53319 |
| Immunoblot/IP | Anti-Beta actin | Abcam | ab6276 |
|  | Anti-GAPDH | Cusabio | CSB-MA000071M0m |
|  | Anti-p53 (DO-1) | Santa Cruz Biotechnology | sc-126 |
|  | Anti-p53 (FL-393) | Santa Cruz Biotechnology | sc-6243 |
|  | Anti-p53 | GeneTex | GTX102965 |
|  | Anti-STAT1 | Santa Cruz Biotechnology | sc-464 |
|  | Anti-Phospho-Stat1 (Tyr701) (58D6) | Cell Signaling Technology | 9167 |
|  | Anti-Mdm2 (SMP14) | Santa Cruz Biotechnology | sc-965 |
|  | Anti-Flag tag | Cusabio | CSB-MA000021M0m |
|  | Anti-Flag tag | Bioss | bs-0965R |
|  | Anti-HA tag | GeneTex | GTX115044 |
|  | Anti-Ubiqitin | Sigma-Aldrich | U0508 |

(Continued on following page)

| Neutralizing | Human IFN-beta Antibody | R & D System | AF814 |
| --- | --- | --- | --- |
|  | Human IFN-gama Antibody | BD Biosciences | 554698 |
|  | Human IFNAR1 Antibody | Abcam | ab10739 |
|  | Human IFNAR2 Antibody | PBL Assay Science | 21385 |
| **Kits** |  |  |  |
|  | Human IFN-beta DuoSet ELISA | R & D System | DY814-05 |
|  | IFN gamma Human Uncoated ELISA Kit | Invitrogen | 88-7316-22 |
|  | RayBio® Cytokine Antibody Arrays G4000 | RayBiotech | AAH-CYT-G4000 |
|  | BD Pharmingen™ FITC Annexin V Apoptosis Detection Kit I | BD Biosciences | 556547 |
| **Reagents** |  |  |  |
|  | Lipopolysaccharide | Sigma-Aldrich | L6529 |
|  | Phorbol-12-myristate-13-acetate | Sigma-Aldrich | p1585 |
|  | Recombinant Human IFN-r | R & D System | 285-IF |
|  | Recombinant Human IFN-β | Peprotech | 300-02BC |
|  | MG132 | Cell Signaling Technology | 2194 |
|  | Cycloheximide | Sigma-Aldrich | 239763-1GMCN |
|  | Protein A/G agrose beads | Invitrogen | 20422 |
|  | Pifithrin-α | Santa Cruz Biotechnology | 63208-82-2 |
|  | JAK inhibitor I | Santa Cruz Biotechnology | 457081-03-7 |
|  | ODN2216 | InvivoGen | tlrl-2216 |
|  | poly I:C HMW | InvivoGen | tlrl-pic |
|  | Imiquimod (R837) | InvivoGen | tlrl-imqs-1 |
| **Primers for real-time PCR** | **Gene** | **Sequences** |  |
|  | IFNAR1 | Forward: CCATTTCGCAAAGCTCAGATT | |
|  |  | Reverse: TAAACCATCCAAAGCCCACATAAC | |
|  | IFNAR2 | Forward: GAAGCATAAACCCGAAATAAAAGG | |
|  |  | Reverse: GCTTGCTCATCACTGTGCTCTAA | |
|  | IFNB1 | Forward: CCTCCAAATTGCTCTCCTGTTG | |
|  |  | Reverse: TTCAATTGCCACAGGAGCTTCT | |
|  | IFNG | Forward: AGATCCCATGGGTTGTGTGTTT | |
|  |  | Reverse: TTAAAGCACTGGCTCAGATTGC | |
|  | JAK1 | Forward: CCTCTGACGTCTGGTCTTTTGG | |
|  |  | Reverse: TGGGCCTATCATTTTCAGGAA | |

(Continued on following page)

|  | JAK2 | Forward: TGTCTTGGGATGGCAGTGTTAG |
| --- | --- | --- |
|  |  | Reverse: TCTGTACCTTATTCGCTTCCTTGTC |
|  | STAT1 | Forward: CCATCCTTTGGTACAACATGC |
|  |  | Reverse: TGCACATGGTGGAGTCAGG |
|  | TBP | Forward: CACGAACCACGGCACTGATT |
|  |  | Reverse: TTTTCTTGCTGCCAGTCTGGAC |
|  | TP53 | Forward: TAACAGTTCCTGCATGGGCGGC |
|  |  | Reverse: AGGACAGGCACAAACACGCACC |
|  | TYK2 | Forward: GCCATCATTCCGCACCAT |
|  |  | Reverse: AGCTGACCTTGCCGAAGTGA |
|  | WIG1 | Forward: CGGCAGAGAATTCCACGTGAT |
|  |  | Reverse: ATCTCTTCGCCAGCTCCAACA |

IHC: immunohistochemistry; IP: immunoprecipitation.
