# Supplementary material for: Tp53 determines the spatial dynamics of M1/M2 tumor-associated macrophages and M1-driven tumoricidal effects
Source: Cell Death Dis. 2025 Jan 22;16(1):38. doi: 10.1038/s41419-025-07346-0 (PMC11754596; doi:10.1038/s41419-025-07346-0)

**Tp53 determines the spatial dynamics of M1/M2 tumor-associated macrophages and M1-driven tumoricidal effects**

**Supplementary data**

**Supplementary Materials and Methods**

**Supplementary Figures and Legends**

**Raw Immunoblot Images for Supplementary Figures**

**Supplementary** **Materials and Methods**

**Cell culture**

Human lung cancer cell lines CL1-0 and H1975 obtained from Dr. Pan-Chyr Yang were cultured in RPMI 1640 medium supplemented with 10% FBS. M0, M1, M2a, and M2c macrophages were polarized from THP-1 monocytic cells using the protocol previously described [1].

**CIBERSORT and cytokine expression analysis**

Data retrieval from the TCGA-LUAD database was conducted using the TCGAbiolinks R package. TP53 somatic mutations for each case were identified from Aggregate GDC MAFs aligned with the hg38 reference genome. STAR-Counts files were downloaded to quantify gene expression. A total of 254 cases were identified with wild-type p53 (wtp53) and 253 cases with mutated p53 (mutp53). For CIBERSORT analysis, FPKM values were utilized as recommended in the guideline [2].

For cytokine expression analysis, the TCGA database was download from cBioportal, which includes 219 cases (118 wtp53 tumors and 101 mutp53 tumors) [3]. Cytokines known to induce M1/M2 TAM polarization were specifically analyzed, including IFN-γ, IL-10, IL-4, TGF-β, GM-CSF and M-CSF.

**Transcriptome analysis of macrophage subtypes**

The whole transcriptomes of M0, M1, M2a, and M2c macrophages were analyzed using the Human HT12-v4 Illumina BeadChip gene expression array, following the manufacturer's instructions (Illumina, San Diego, CA). The arrays were read and fluorescence signals captured using the Illumina Bead Array Reader. Data analysis was performed with GenomeStudio software. The array data is available in the GEO database (GSE116783). Differentially expressed genes were identified using the Mann-Whitney differential expression algorithm (p < 0.05) and were defined by a fold change of more than 2 between groups.

**Quantitative real-time PCR**

The mRNA level was quantified by SYBR Green real-time RT-PCR on an ABI Prism 7900HT sequence detection system (Applied Biosystems/Thermo Fisher Scientific, Foster City, CA). The TATA box-binding protein (TBP) was used as an internal control. The relative expression level of the target gene, normalized to TBP, was calculated using the formula: ΔCT_Target = CT_TBP – CT_Target. The fold change in expression between different treatments was determined by the 2^-ΔΔCT^ method. Primer sequences are provided in Supplementary Table 5.

**Supplementary Figures and Legends**


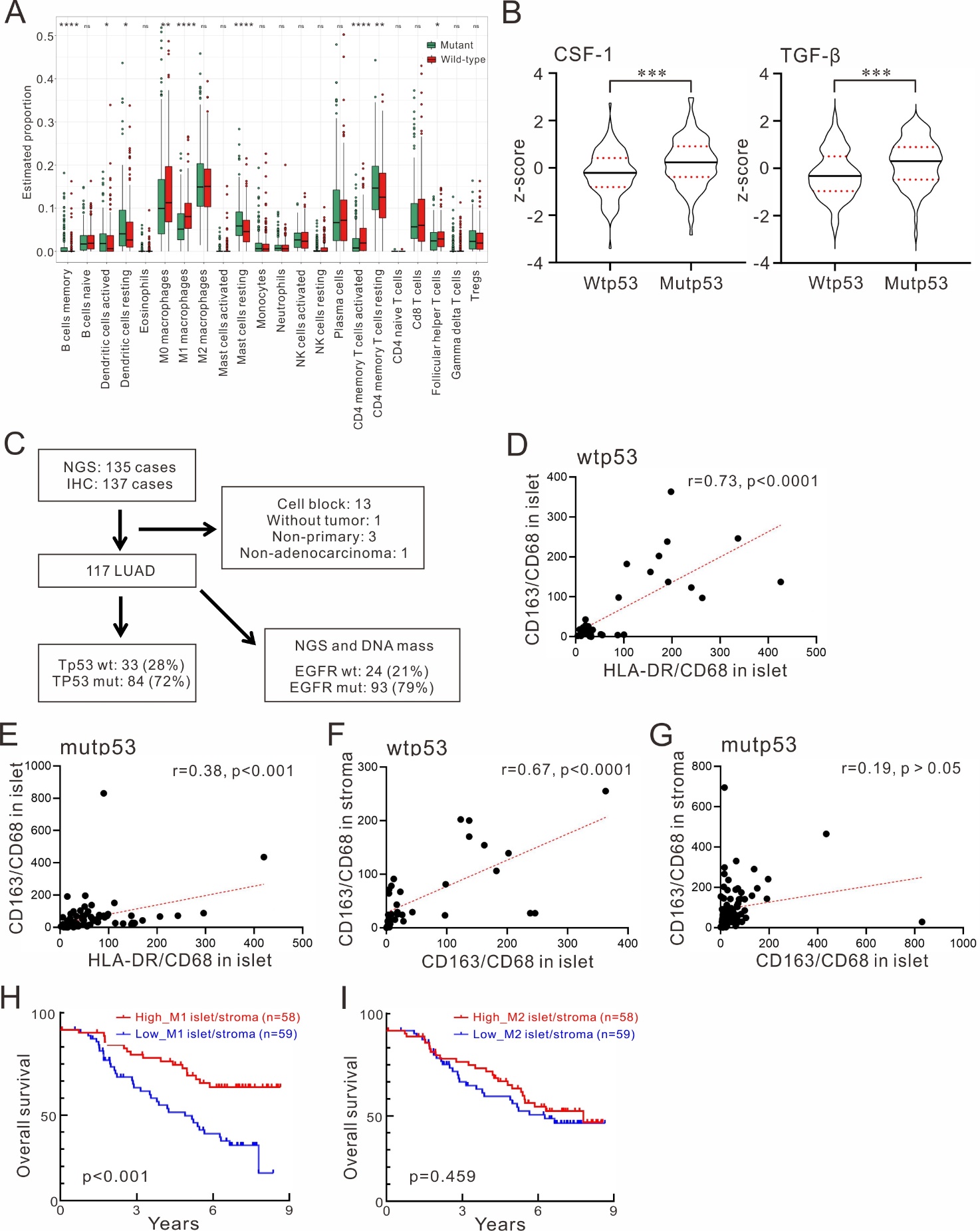


**Supplementary Figure 1. Correlation of M1/M2 TAMs in wtp53 and mutp53 tumors and their impact on overall survival.**

(A) Comparison of immune cell proportions in 254 lung adenocarcinomas with wtp53 and 253 lung adenocarcinomas with mutp53. **P*-value < 0.05; ** *P*-value < 0.01; **** *P*-value < 0.0001; ns, not significant. (B) The *CSF1* and *TGFB2* expressions in lung adenocarcinomas with wtp53 and mutants. The violent plots are presented as median ± quartiles. (C) Flowchart of clinical specimen selection and analysis. A total of 137 cases were analyzed for M1/M2 TAMs quantification using immunohistochemistry (IHC), while 135 cases underwent deep sequencing using next-generation sequencing (NGS) to detect EGFR and TP53 mutations. After excluding unqualified specimens, 117 lung adenocarcinomas (LUAD) were selected for further analysis. EGFR and TP53 mutations were annotated according to the Cosmic and ClinVar databases. (D) Correlation of M1 and M2 TAMs densities in tumor islets of wtp53 tumors. (E) Correlation of M1 and M2 TAM densities in tumor islets of mutp53 tumors. (F) Correlation between M2 TAM densities in tumor islets and stroma of wtp53 tumors. (G) Correlation between M2 TAM densities in tumor islets and stroma of mutp53 tumors. M1 TAMs were identified using HLA-DR/CD68 double staining, and M2 TAMs using CD163/CD68 double staining. Cell density was measured in cells/mm². Pearson correlation coefficient (r) was used for correlation analysis. (H) (I) Kaplan–Meier survival analysis of 117 lung adenocarcinomas. Classification of patients based on the ratio of M1 and M2 TAM density in islets to stroma. *P*-value estimated by log-rank test. *P*-value < 0.05 considered statistically significant.


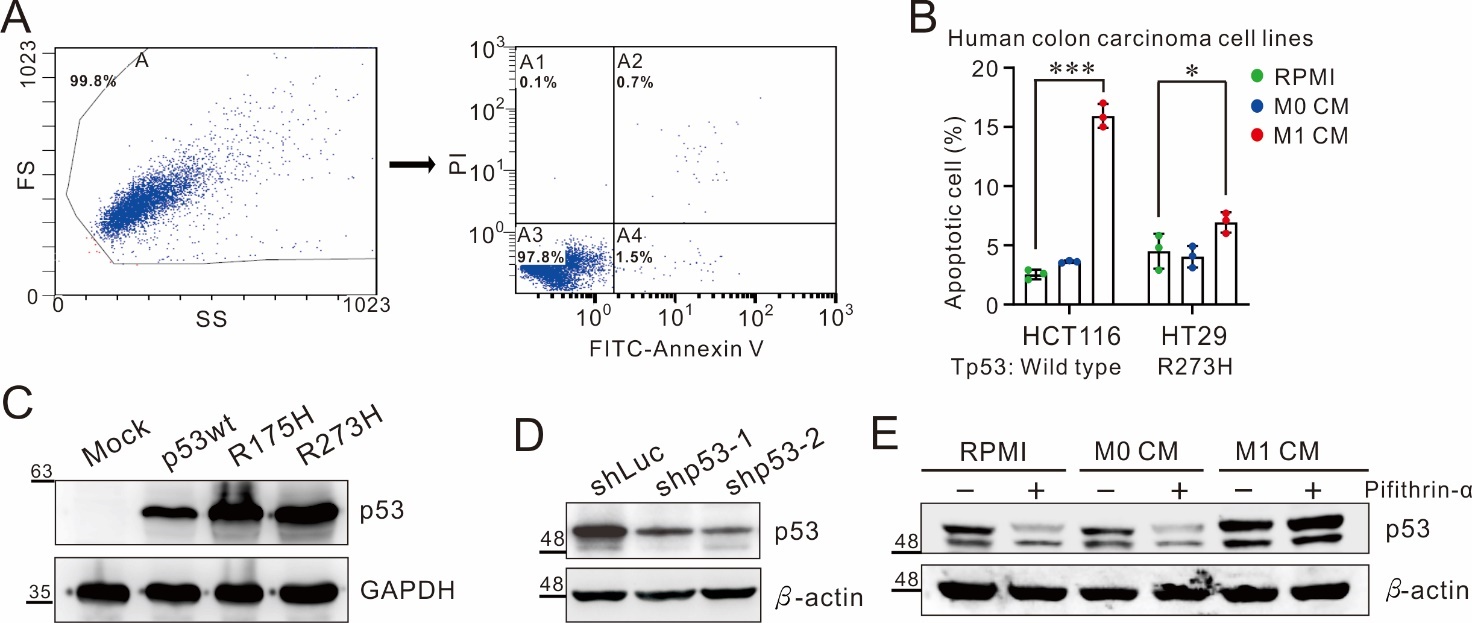


**Supplementary Figure 2. Upregulation of p53 expression in response to M1 CM.**

(A) Gating was used to determine the percentage of apoptotic cells via flow cytometry. Throughout this study, the percentages of early apoptotic cells (Annexin V^+^/PI^-^, A4) and late apoptotic cells (Annexin V^+^/PI^+^, A2) were calculated. (B) Apoptotic response of the colon cancer cell lines to M1 CM stimulation. Data are represented as mean ± SD; n = 3. (C) Ectopic expression of p53 in H1299 cells. The cells were transfected with 2 μg of either pcDNA3.1-Flag, wild-type p53, p53-R175H, or p53-R273H plasmids. (D) Silencing p53 expression in A549 cells via lentivirally expressed shRNAs targeting TP53. (E) M1 CM enhanced p53 expression. A549 cells were pre-treated with 30 μM pifithrin-α for 24 hours, followed by incubation in CM with a continuous presence of 30 μM pifithrin-α for 5 days. Data are representative of at least two independent experiments.


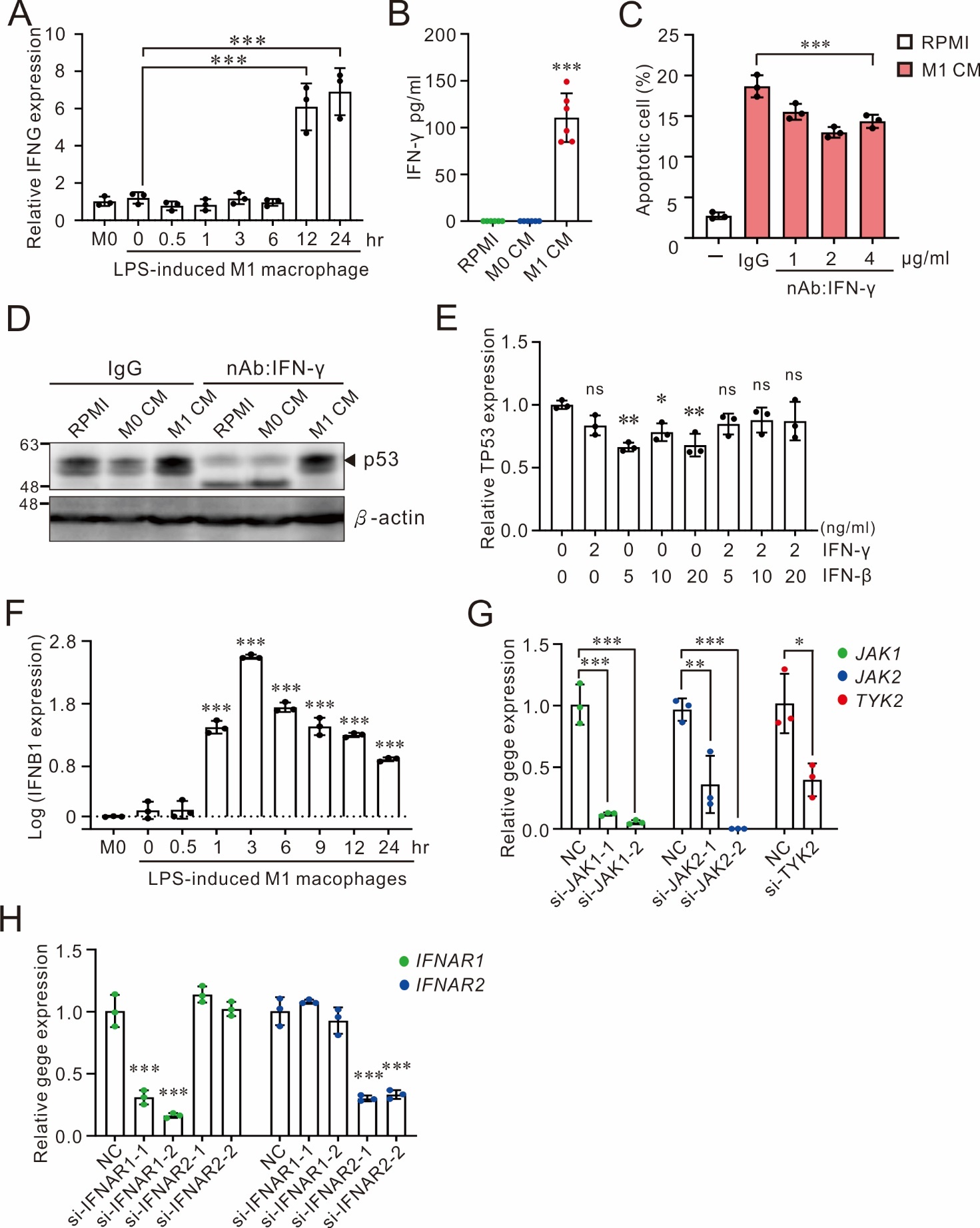


**Supplementary Figure 3. IFNs expression by M1 macrophages and induction of apoptosis via the IFNAR/JAK signaling pathway**.

(A) *IFNG* expression in M1 macrophages following LPS induction. M0 macrophages were primed with 20 ng/ml IFN-γ for 16 hours before being induced with LPS for indicated durations. (B) IFN-γ concentration in three batches of M1 CM measured via ELISA. High-glucose RPMI supplied with 10% FBS served as the medium control. (C) Effect of IFN-γ neutralization on M1-induced apoptosis. M1 CM, supplemented with the indicated concentrations of IFN-γ neutralizing antibody (nAb) or 4 μg/ml IgG control, was used to treat A549 cells for 3 days, followed by apoptosis assessment using flow cytometry. (D) Effect of IFN-γ neutralization on p53 expression in A549 cells. RPMI, M0 and M1 CM, supplemented with the indicated concentrations of IFN-γ neutralizing antibody (nAb) or 4 μg/ml IgG control, was used to treat A549 cells for 3 days, followed by immunoblot assessment. (E) *TP53* expression in A549 cells following treatment with recombinant human IFNs. (F) *IFNB1* expression in M1 macrophages following LPS induction. M0 macrophages were primed with 20 ng/ml IFN-γ for 16 hours before being induced with LPS for the indicated durations. (G) Assessment of the specificity and efficiency of si-JAK1, si-JAK2, and si-TYK2 in A549 cells. Student’s t-test was applied for si-TYK2 comparisons. (H) Assessment of the specificity and efficiency of si-IFNAR1 and si-IFNAR2 in A549 cells. One-way ANOVA followed by Tukey’s post hoc test was used for compared to 0hr, blank or NC (negative control). ns, not significant; *P*-value > 0.05; * *P*-value < 0.05; ** *P*-value < 0.01; *** *P*-value < 0.001. Data are represented as mean ± SD (n = 3) and representative of at least two independent experiments.


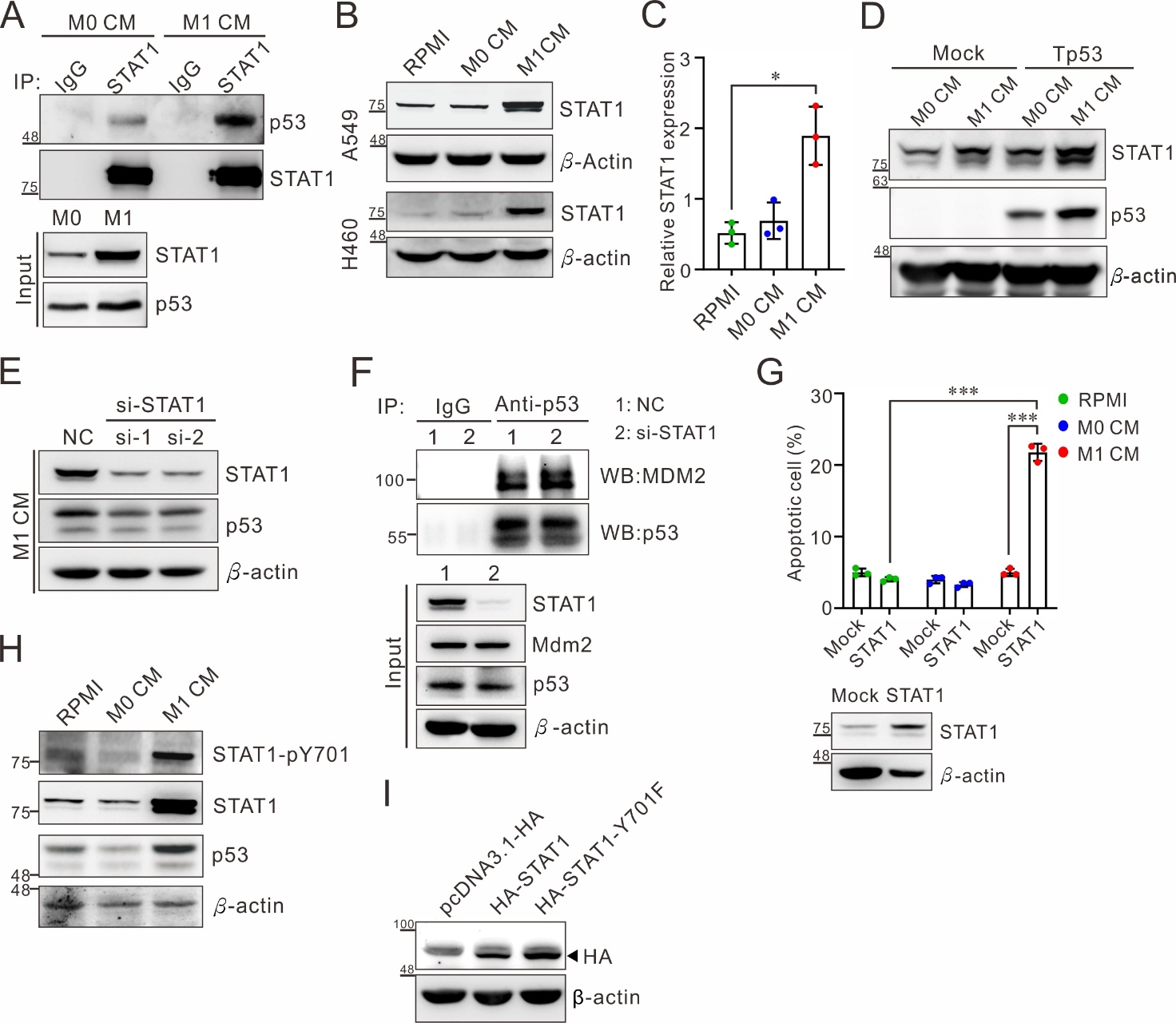


**Supplementary Figure 4. STAT1 interacted with p53 and mediated M1-indueced apoptosis.**

(A) Interaction between p53 and STAT1 enhanced by M1 CM. Immunoprecipitation is used an anti-STAT1 antibody and immunoblot with anti-STAT1 and anti-p53 antibodies. Cell lysates were prepared from A549 cells cultured in M0 or M1 CM for 3 days. (B) STAT1 expression in A549 and H460 cells cultured in RPMI, M0, and M1 CM. (C) *STAT1* expression in A549 cells cultured in RPMI, M0, and M1 CM. (D) Ectopic expression of p53 and endogenous STAT1 in H1299 cells enhanced by M1 CM. (E) Silencing STAT1 reduced p53 expression in A549 cells cultured in M1 CM. (F) The interaction of p53 and MDM2 in STAT1 knockdown A549 cells. si-STAT1 was delivered into A549 cells, followed by culture in M1 CM for 30 hours. Prior to harvest, cells were treated with 30 μM MG132 for 4 hours. Cell lysates were immunoprecipitated using an anti-p53 (DO-1) antibody and analyzed by immunoblot. (G) Ectopic expression of STAT1 enhanced apoptosis in A549 cells cultured in M1 CM by the second day. Data are represented as mean ± SD; n = 3. (H) Activation of STAT1-Y701 phosphorylation by M1 CM. A549 cells treated with RPMI, M0, and M1 CM for 3 days were assayed by immunoblot. (I) H1299 cells stably expressing HA-STAT1, HA-STAT1-Y701F, and vector control, with arrows indicating HA expression. One-way and two-way ANOVA with multiple comparison tests were used. ns, not significant; *P*-value > 0.05; * *P*-value < 0.05; ** *P*-value < 0.01; *** *P*-value < 0.001. Data are representative of at least two independent experiments.


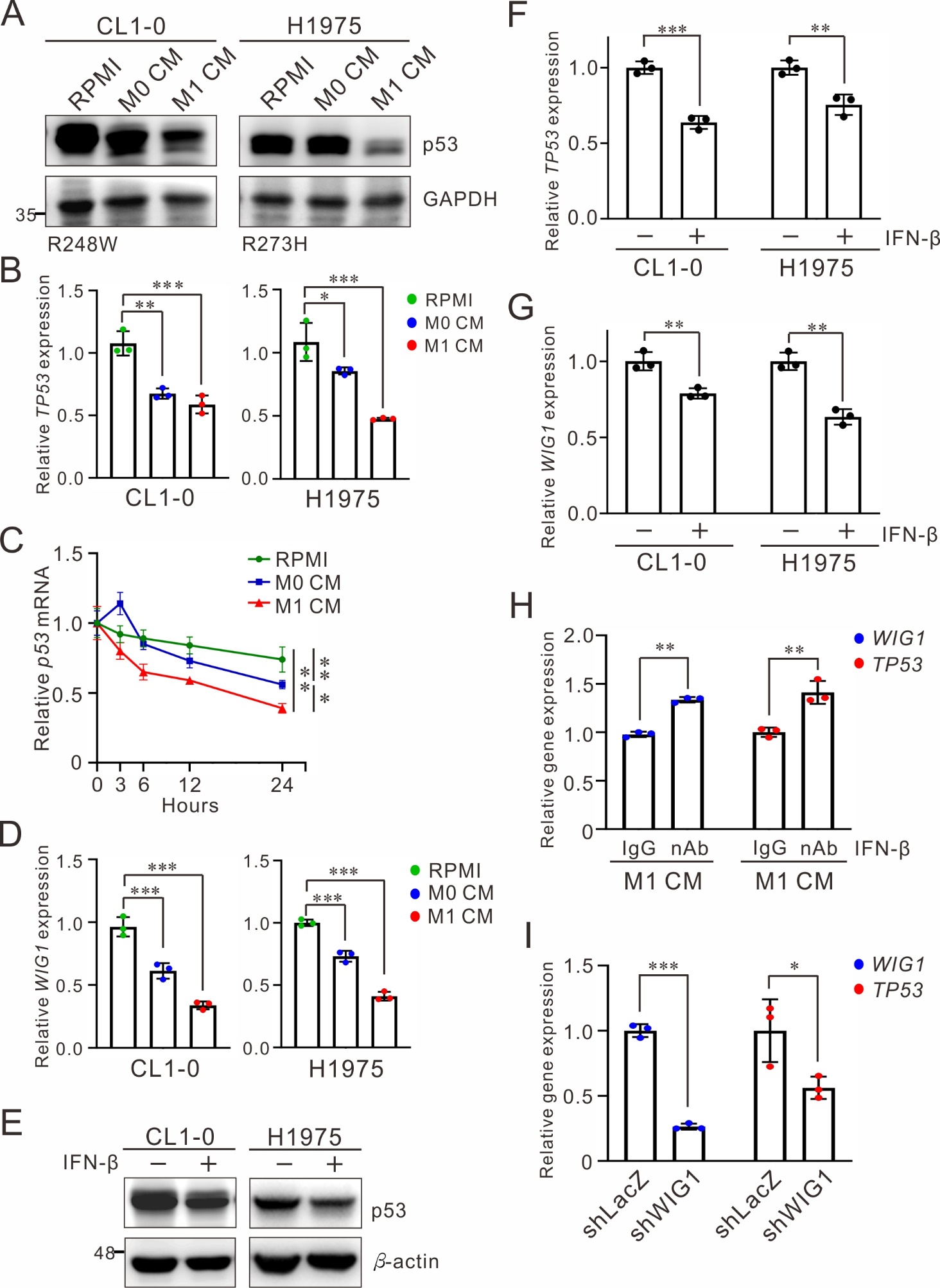


**Supplementary Figure 5. The effect of M1 CM on the lung cancer cells harboring p53 mutant.**

The p53 were reduced in M1 CM treatment CL1-0 and H1975 cells at (A) protein and (B) mRNA levels. (C) The mRNA stability of TP53 in H1975 cells treated with RPMI, M0 and M1 CM. Cells were treated with 10 ng/mL Actinomycin D and harvested at indicated time. (D) The *WIG1* expression in CL1-0 and H1975 cells treated with RPMI, M0 and M1 CM for 3 days. (E) The IFN-β decreased p53 expression in CL1-0 and H1975 cells. (F) The *TP53* expression was deceased in CL1-0 and H1975 cells after IFN-β treatment. (G) The *WIG1* expression was deceased in CL1-0 and H1975 cells after IFN-β treatment. Cells treated with 20 ng/mL IFN-β for 3 days. (H) Neutralizing IFN-β in M1 CM reversed the reduction of *TP53* and *WIG1* expression in H1975 cells. Cells were treated with M1 CM supplemented with 2 μg/mL IFN-β neutralized antibody or IgG for 3 days. (I) The *TP53* expression in H1975 cells after WIG1 silencing. One-way ANOVA followed by Tukey’s post hoc test was used in multiple group comparisons, while Student’s t-test was applied for pairwise comparison. * *P*-value < 0.05; ** *P*-value < 0.01; *** *P*-value < 0.001.


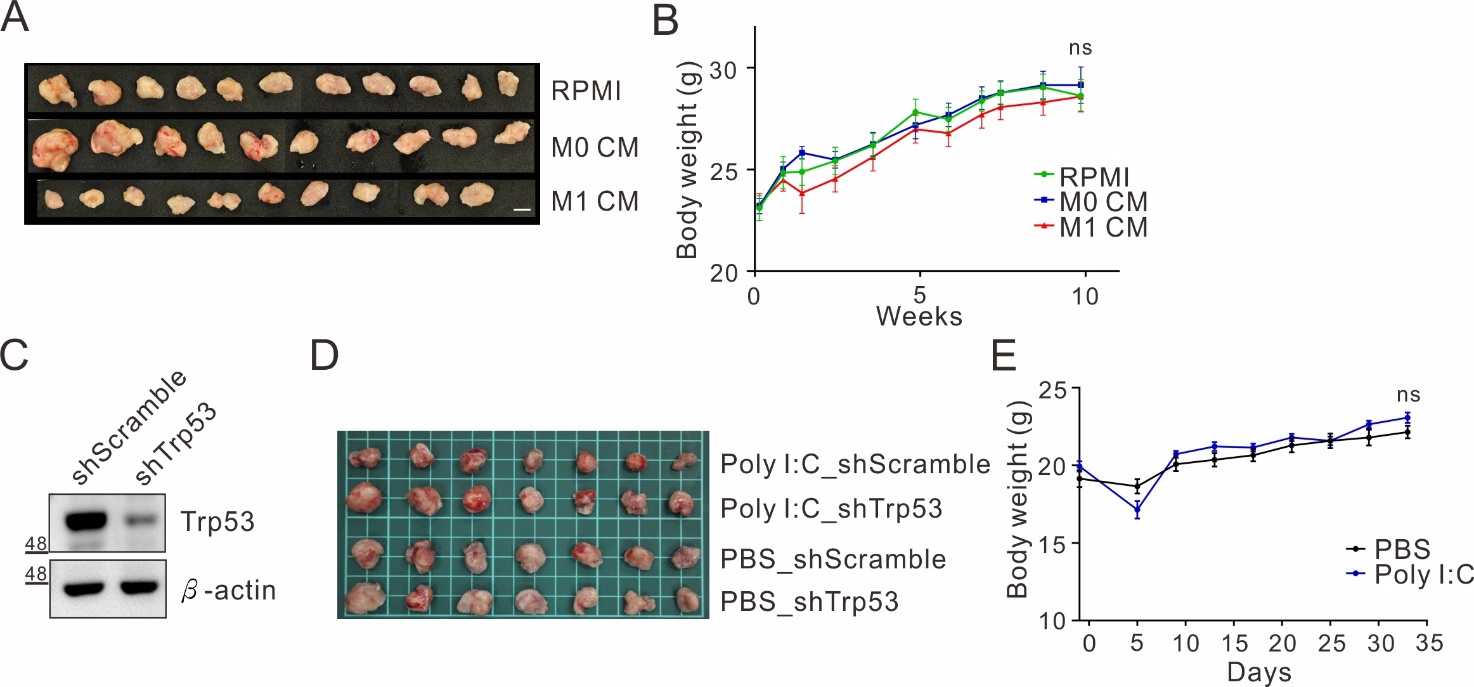


**Supplementary Figure 6. Assessing tumorigenesis following treatment with CM or Poly I:C.**

(A) Images of the tumors treated with CM post-10-week inoculation. SCID mice were subcutaneously inoculated with A549 cells and received intratumoral injections of 50μl M0 CM, M1 CM or RPMI three times a week upon reaching a tumor volume of 100 mm³. The bar represents 1 cm. (B) Body weight of SCID mice was recorded throughout the experiment until euthanasia. Data are represented as mean ± SD. (C) Immunoblot analysis of murine Trp53 in CMT64 cells with stable expression of either shScramble or shTrp53. (D) Tumor images at day 33 post-inoculation. CMT64 cells stable expressing shScramble or shTrp53 were subcutaneously inoculated into the dorsal region of C57BL/6 mice on day 0. PBS or poly I:C was administered intravenously on days 5, 9, 13, and 17. The grid represents 1 cm^2^. (E) Body weight of C57BL/6 mice was recorded on day -1 and then every four days thereafter. Data are represented as mean ± SD; n=7. One-way ANOVA with multiple comparison tests and Student’s t-test were used. ns, not significant.


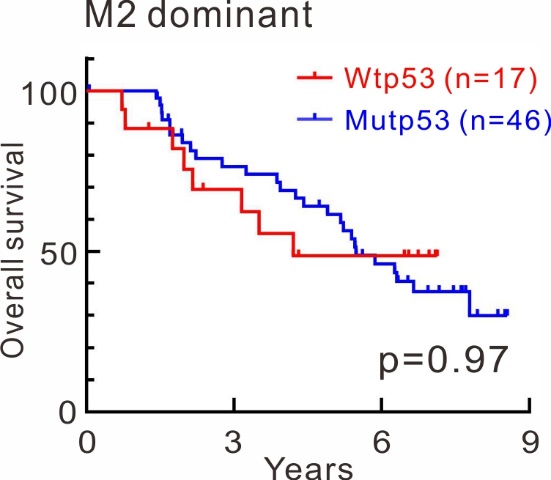


**Supplementary Figure 7. Overall survival of M2 dominant lung adenocarcinomas classified by TP53 status.**

Kaplan–Meier survival analysis comparing overall survival in M2-dominant lung adenocarcinomas between cases with wild-type p53 (wtp53) and mutant p53 (mutp53). *P*-value for overall survival was determined using log-rank test.

**References**

1. Yuan A, Hsiao YJ, Chen HY, Chen HW, Ho CC, Chen YY, et al. Opposite Effects of M1 and M2 Macrophage Subtypes on Lung Cancer Progression. Sci Rep. 2015;5:14273. doi: 10.1038/srep14273. PubMed PMID: 26399191; PubMed Central PMCID: PMCPMC4585843.

2. Newman AM, Liu CL, Green MR, Gentles AJ, Feng W, Xu Y, et al. Robust enumeration of cell subsets from tissue expression profiles. Nat Methods. 2015;12(5):453-7. doi: 10.1038/nmeth.3337. PubMed PMID: 25822800; PubMed Central PMCID: PMCPMC4739640.

3. Riaz N, Havel JJ, Makarov V, Desrichard A, Urba WJ, Sims JS, et al. Tumor and Microenvironment Evolution during Immunotherapy with Nivolumab. Cell. 2017;171(4):934-49 e16. doi: 10.1016/j.cell.2017.09.028. PubMed PMID: 29033130; PubMed Central PMCID: PMCPMC5685550.

**Raw Immunoblot Images for Supplementary Figures**


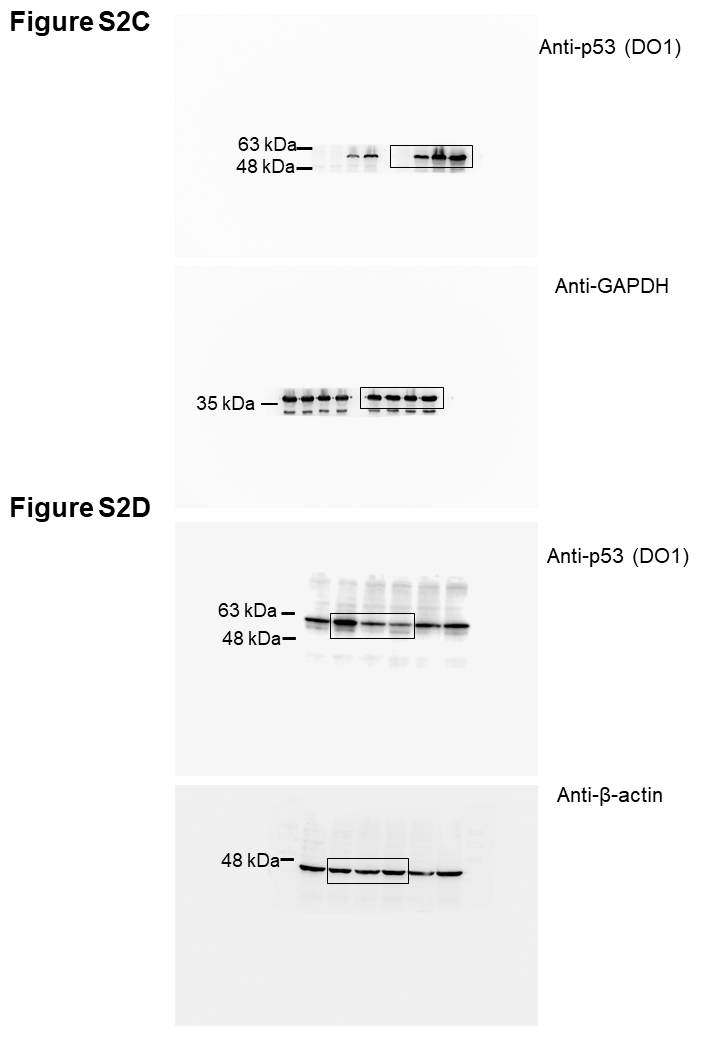


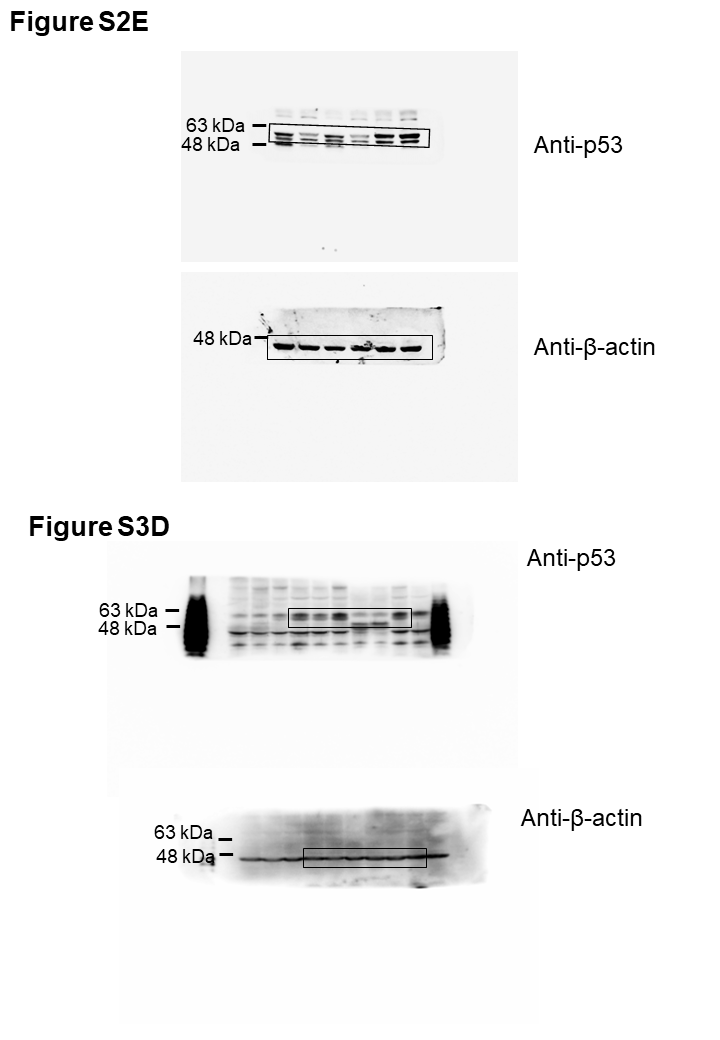


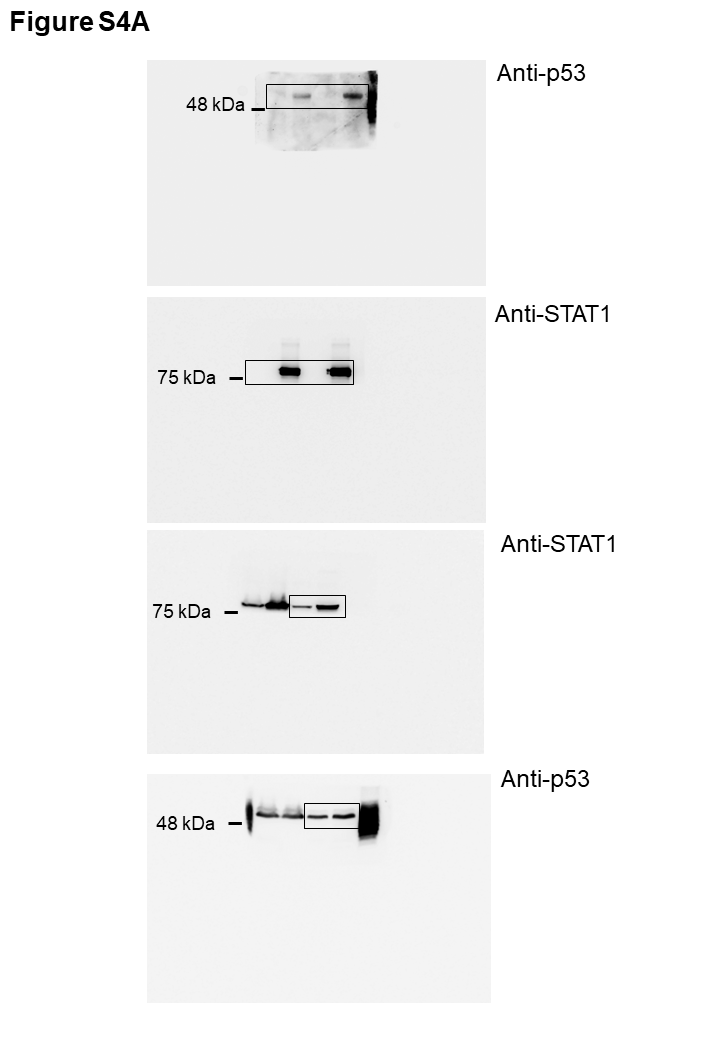


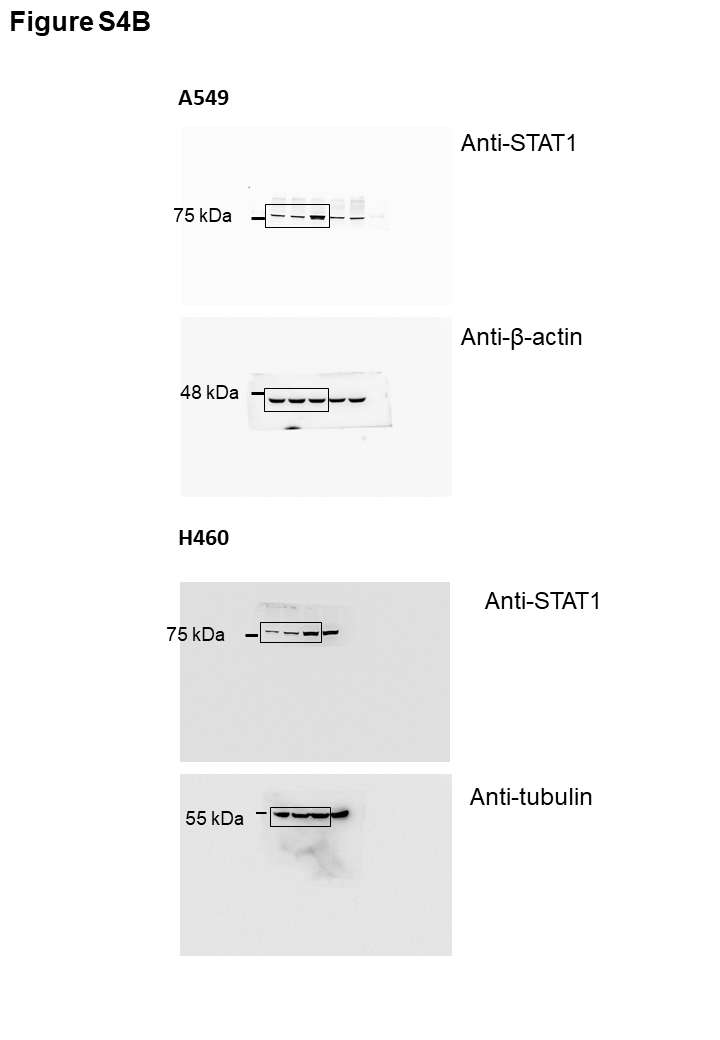


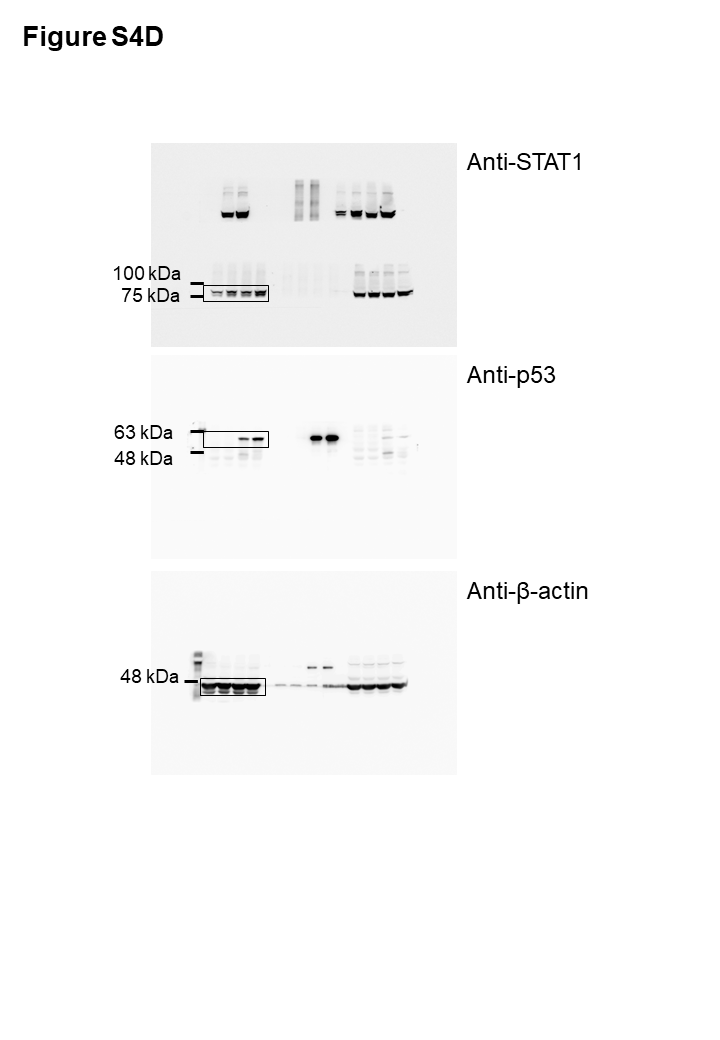


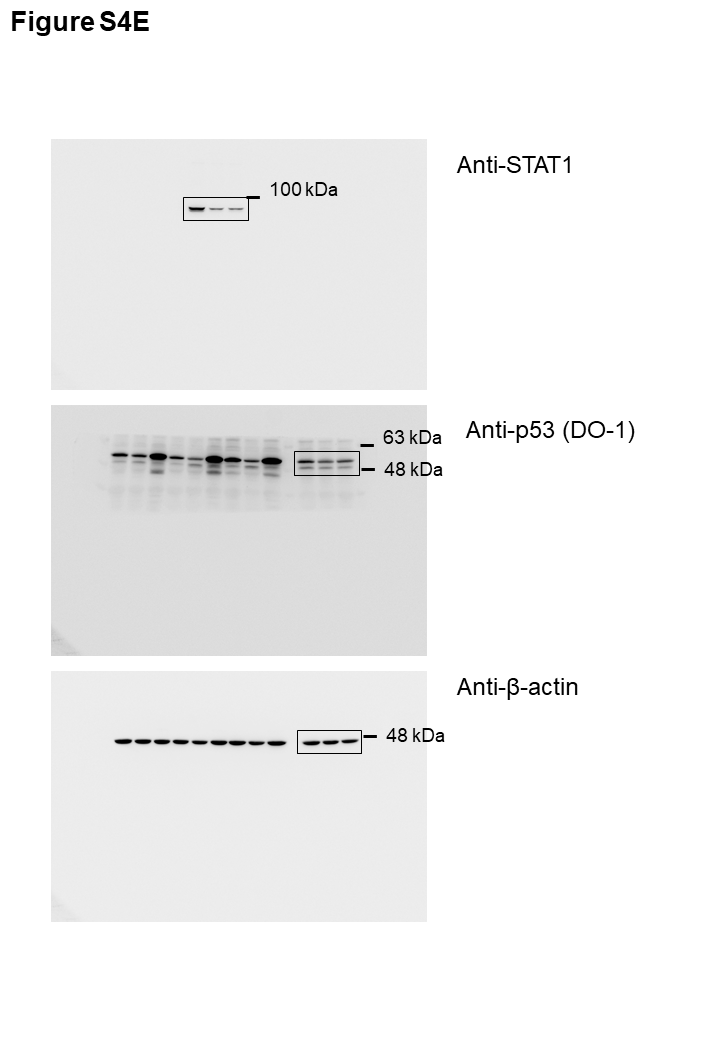


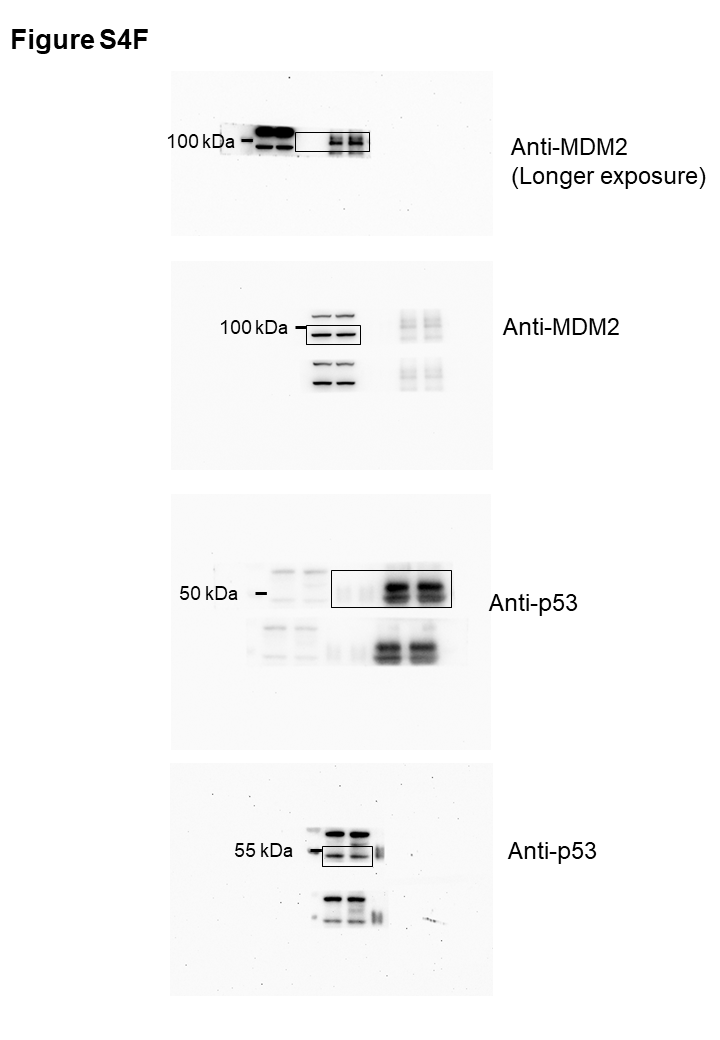


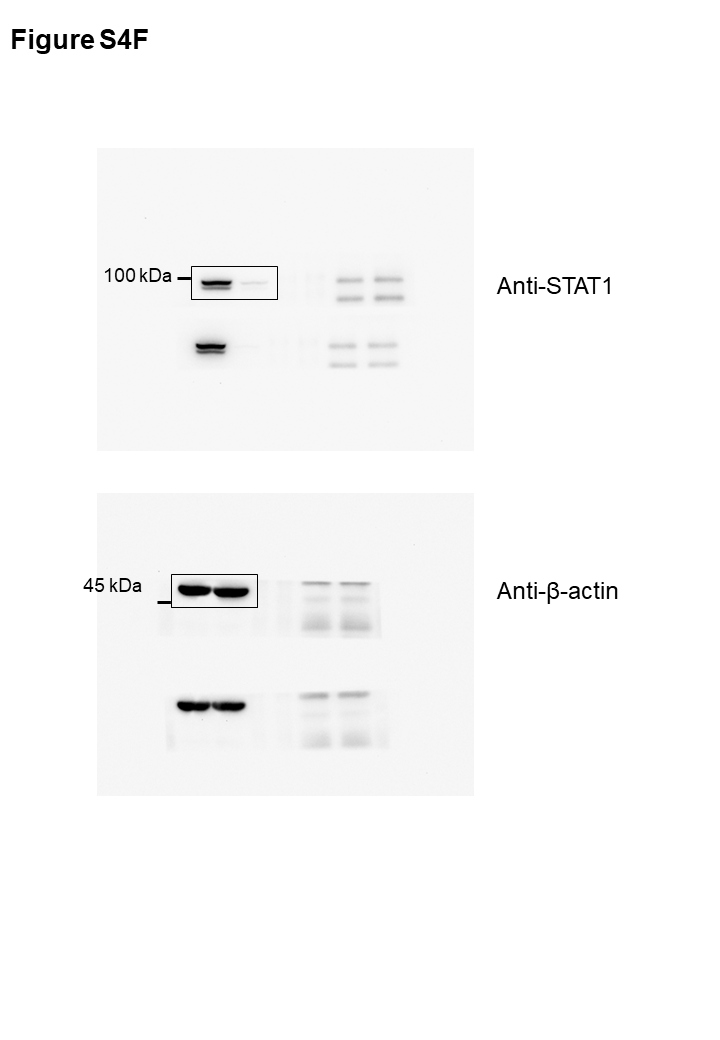

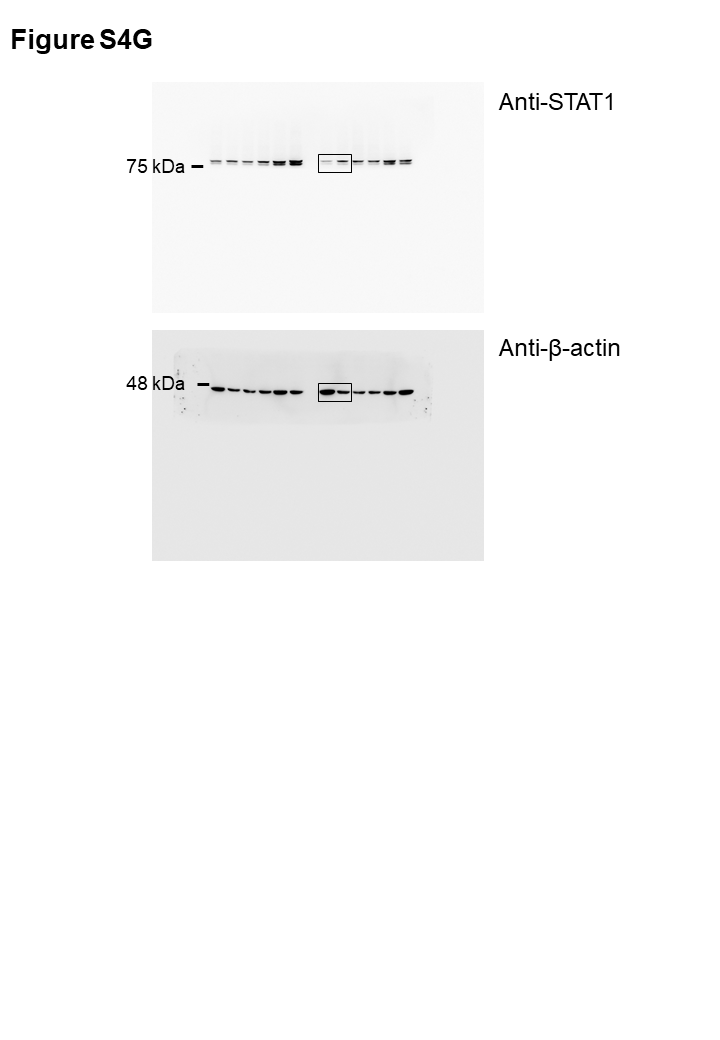

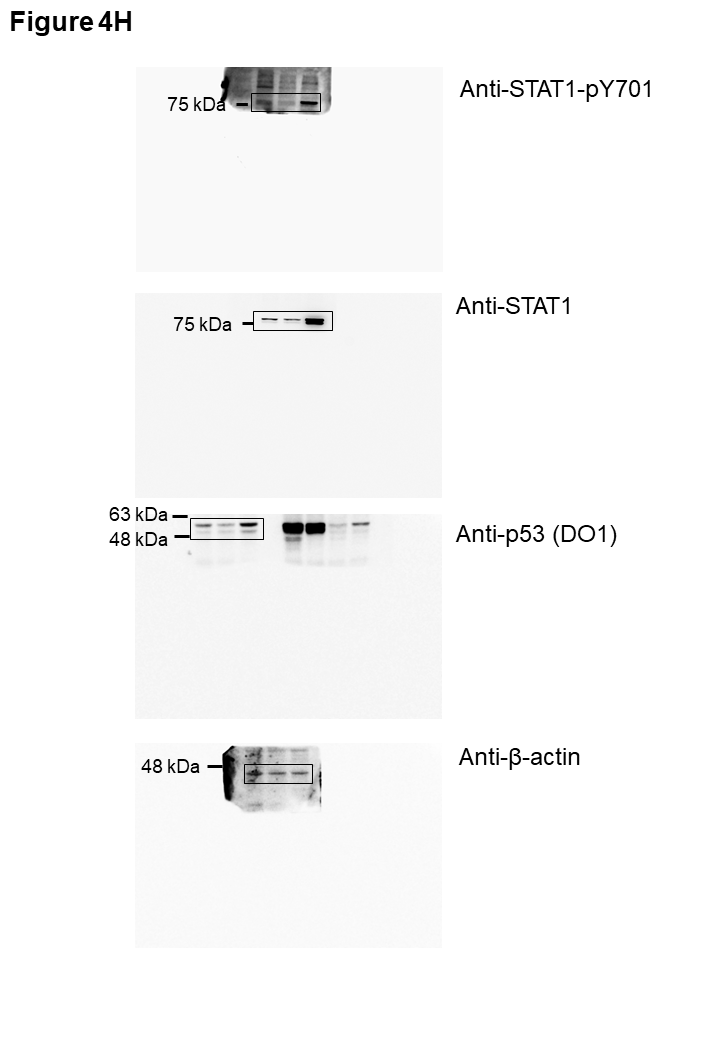

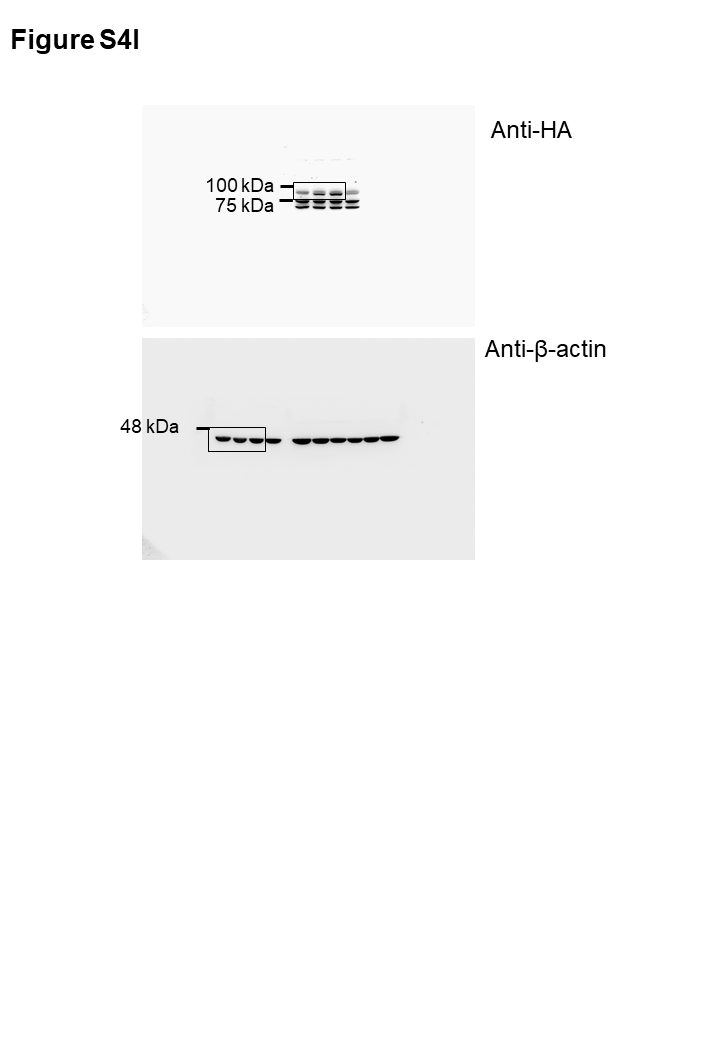

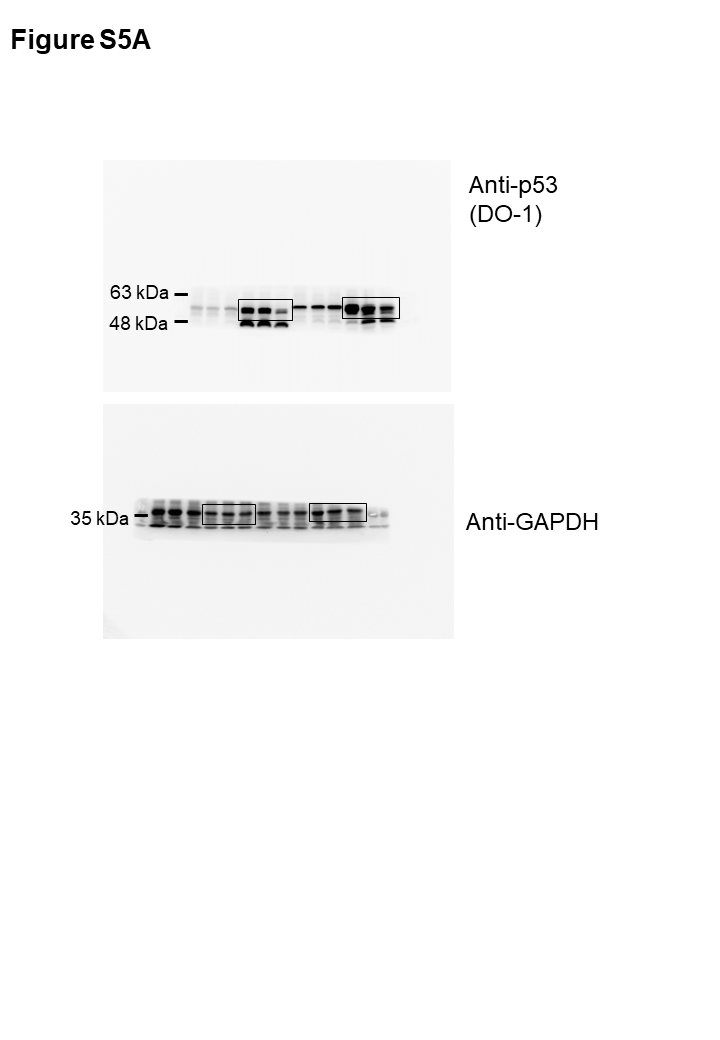

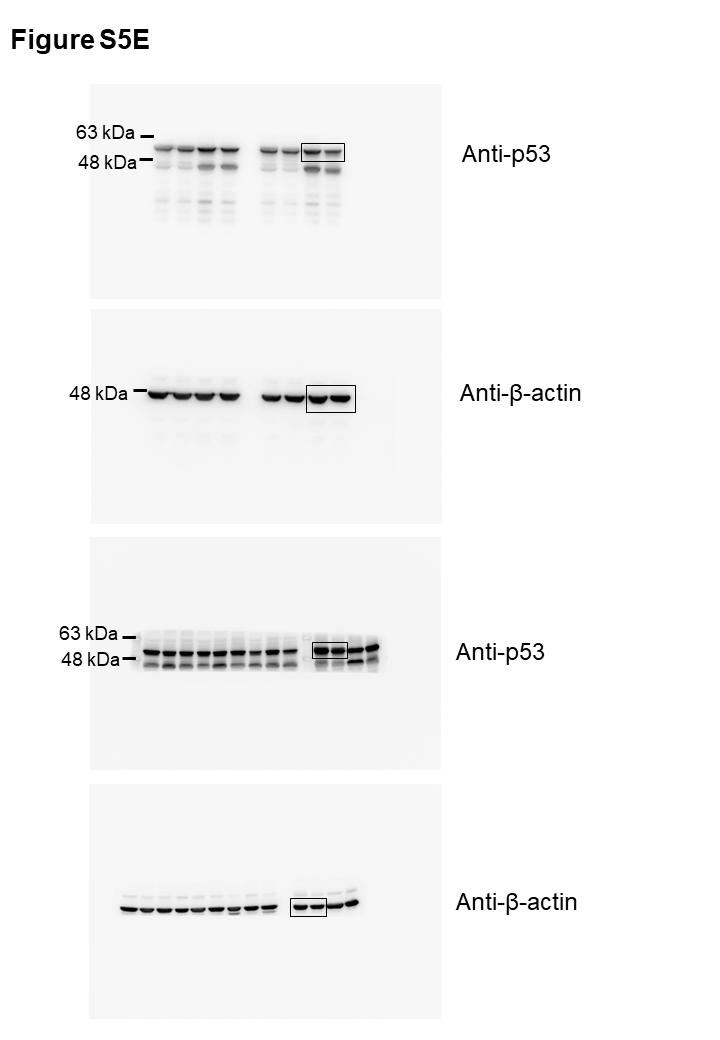

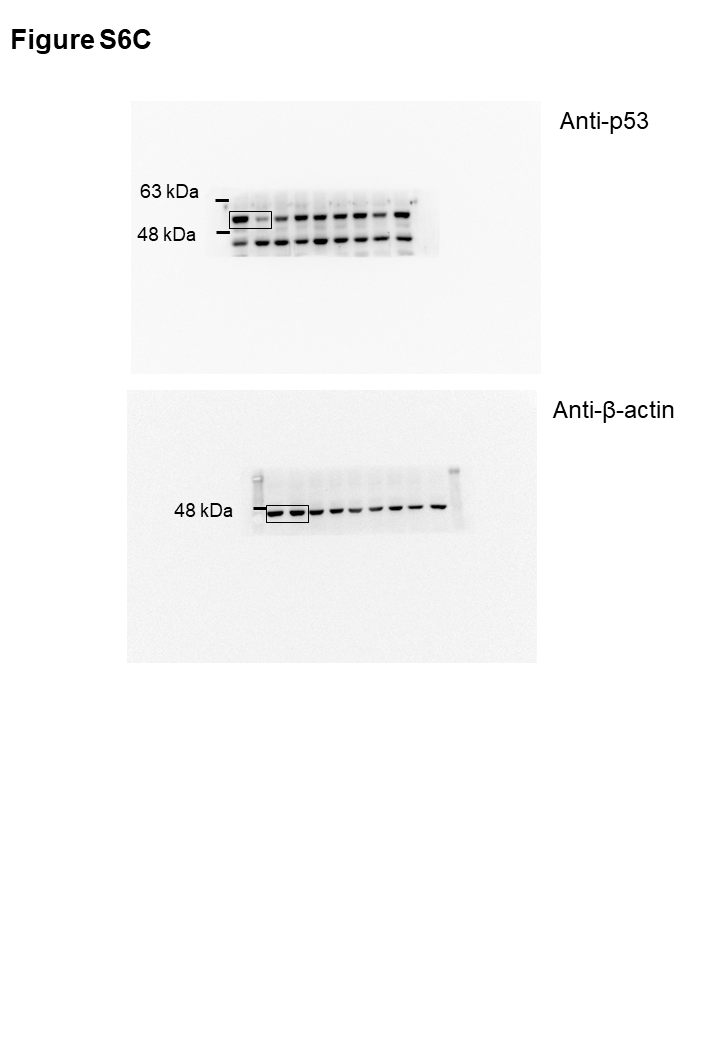

Supplement: Supplementary file 2 — Supplementary Figures [file 41419_2025_7346_MOESM2_ESM.docx]
