# Supplementary material for: Tp53 determines the spatial dynamics of M1/M2 tumor-associated macrophages and M1-driven tumoricidal effects
Source: Cell Death Dis. 2025 Jan 22;16(1):38. doi: 10.1038/s41419-025-07346-0 (PMC11754596; doi:10.1038/s41419-025-07346-0)

## Figure 2E

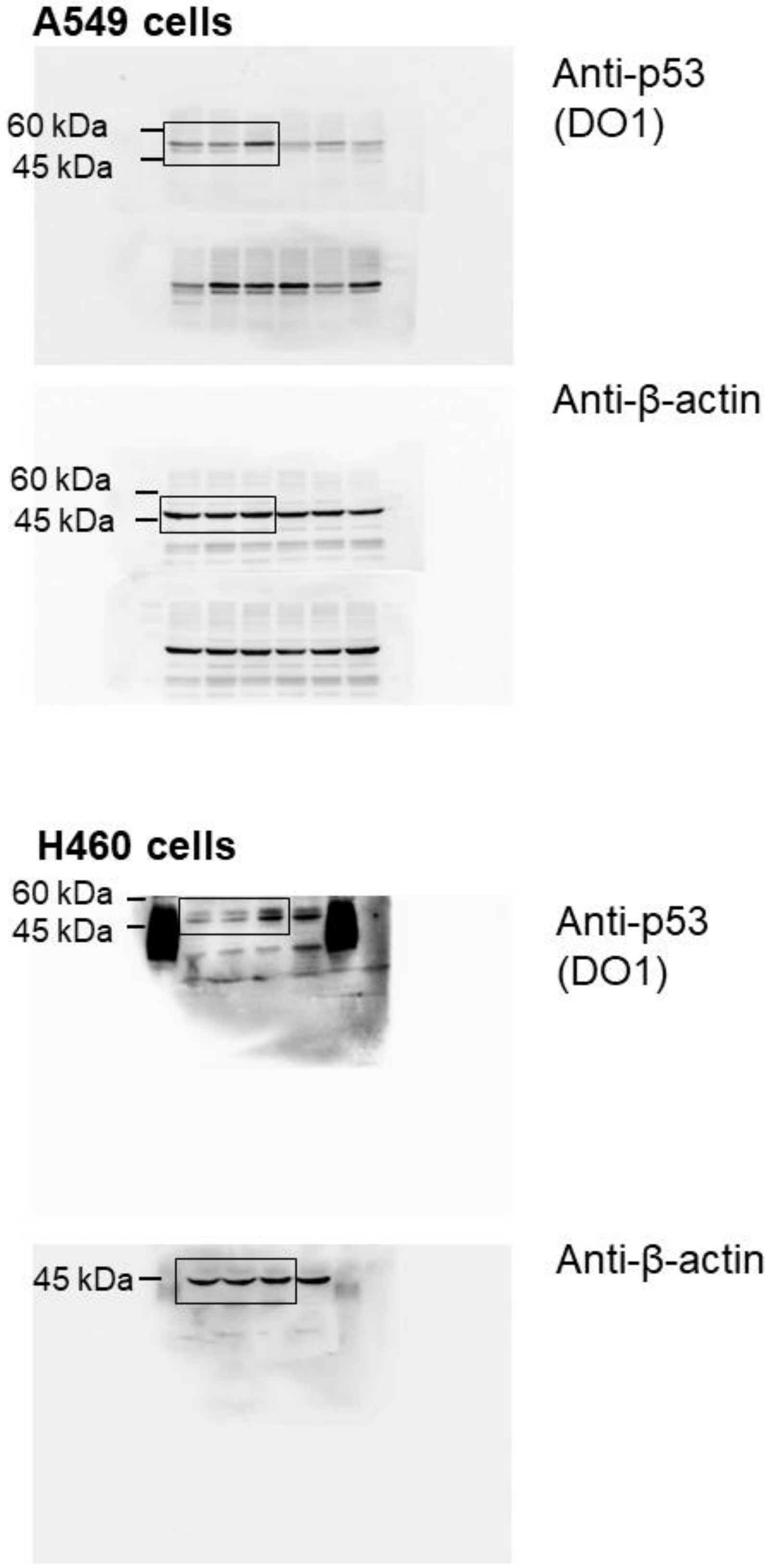
A549 cells
60 kDa
45 kDa
Anti-p53
(DO1)
60 kDa
45 kDa
Anti-β-actin
H460 cells
60 kDa
45 kDa
Anti-p53
(DO1)
45 kDa
Anti-β-actin

## Figure 2F

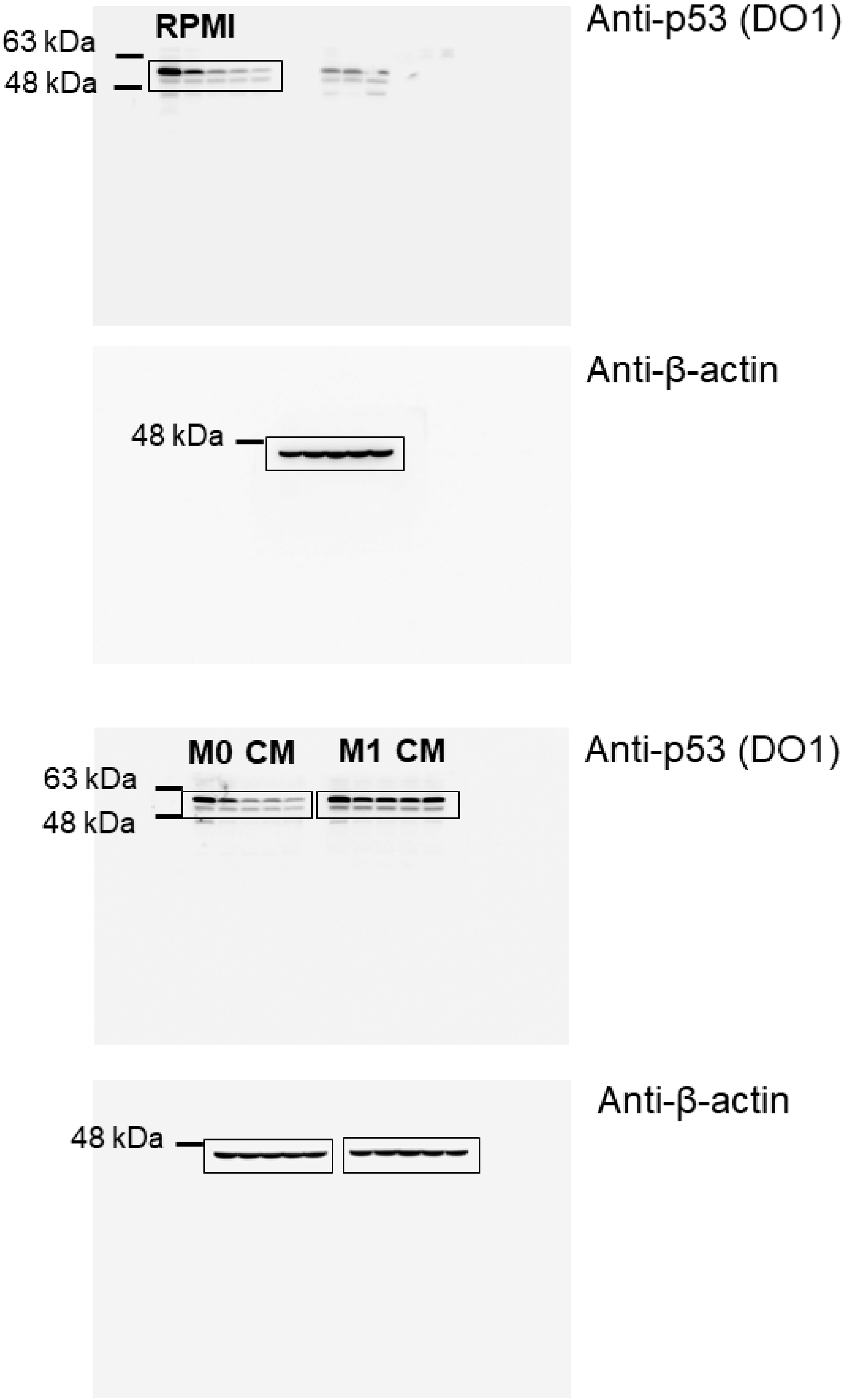
RPMI
63 kDa
48 kDa
Anti-p53 (DO1)
48 kDa
Anti-β-actin
M0 CM
M1 CM
63 kDa
48 kDa
Anti-p53 (DO1)
48 kDa
Anti-β-actin

**Figure 2G**

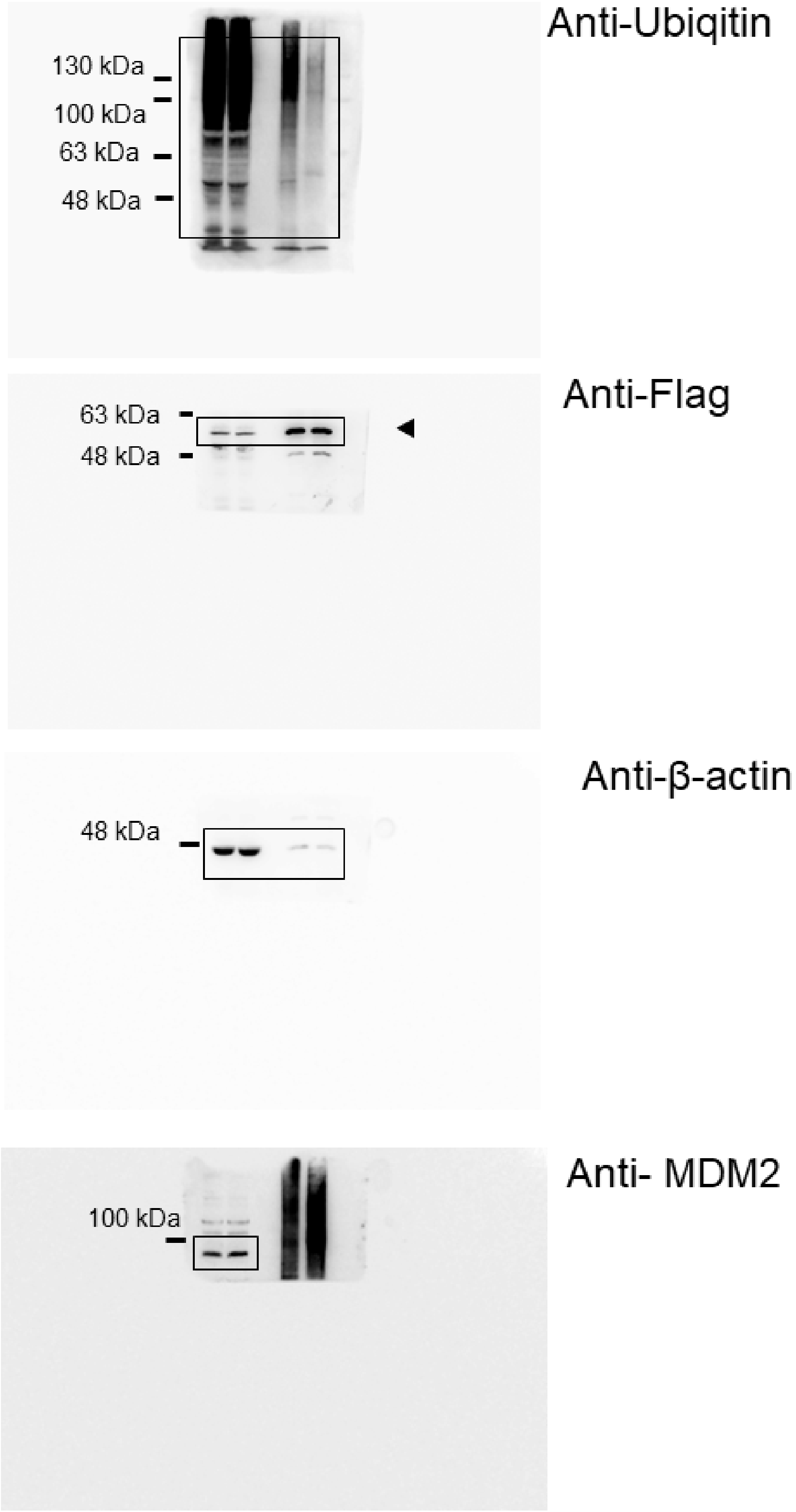
Anti-Ubiqitin
130 kDa
100 kDa
63 kDa
48 kDa
Anti-Flag
63 kDa
48 kDa
Anti-β-actin
48 kDa
Anti- MDM2
100 kDa

# Figure 2H

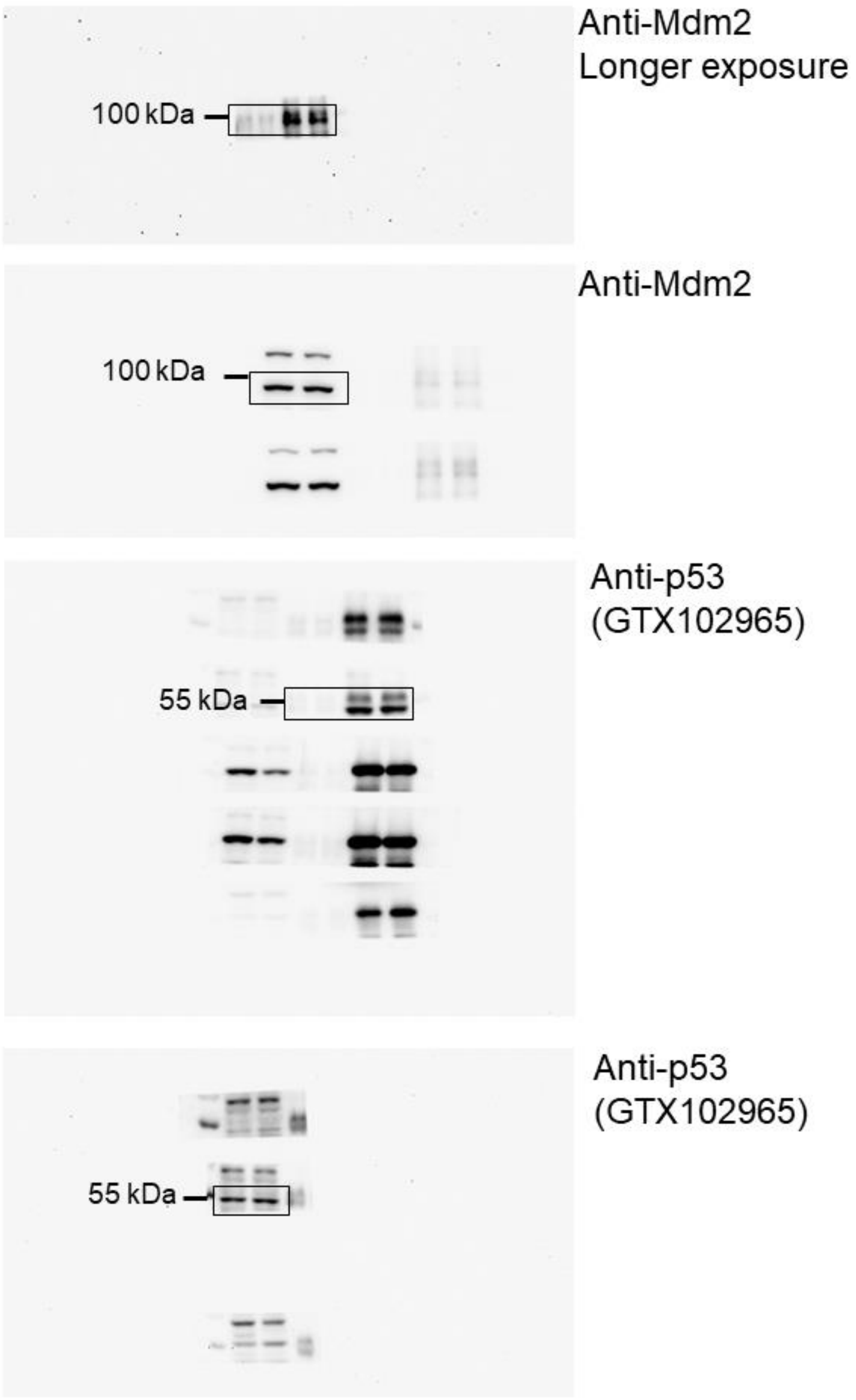
Anti-Mdm2
Longer exposure
100 kDa
Anti-Mdm2
100 kDa
Anti-p53
(GTX102965)
55 kDa
Anti-p53
(GTX102965)
55 kDa

## Figure 3B

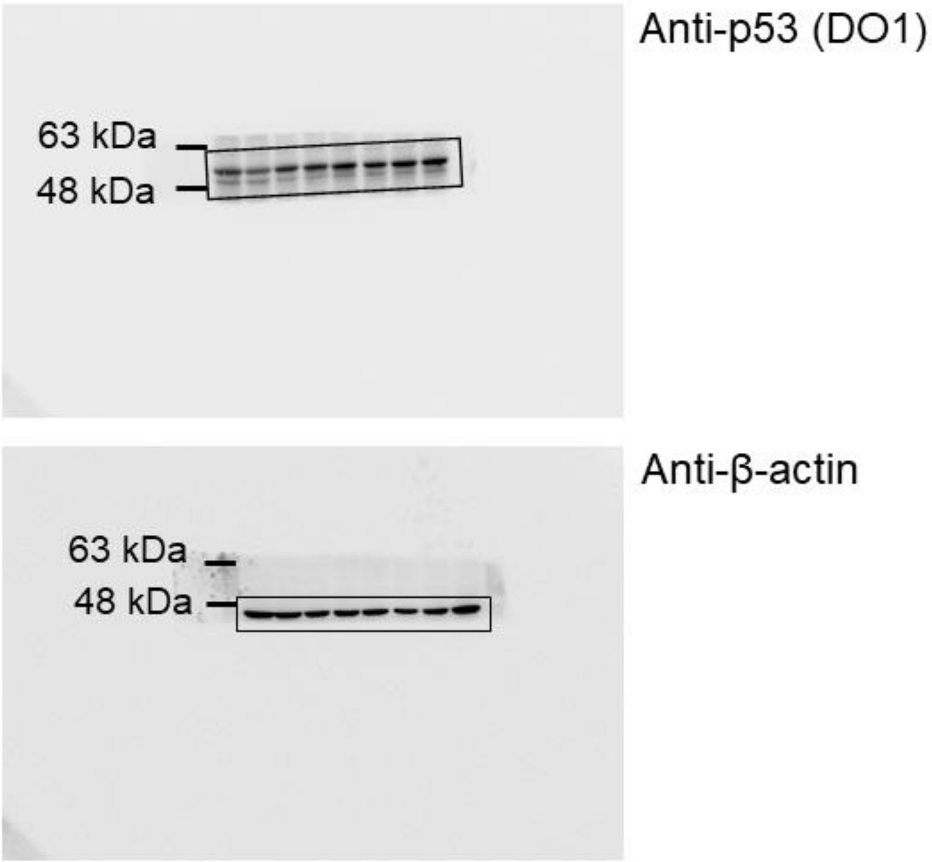

# Figure 4A

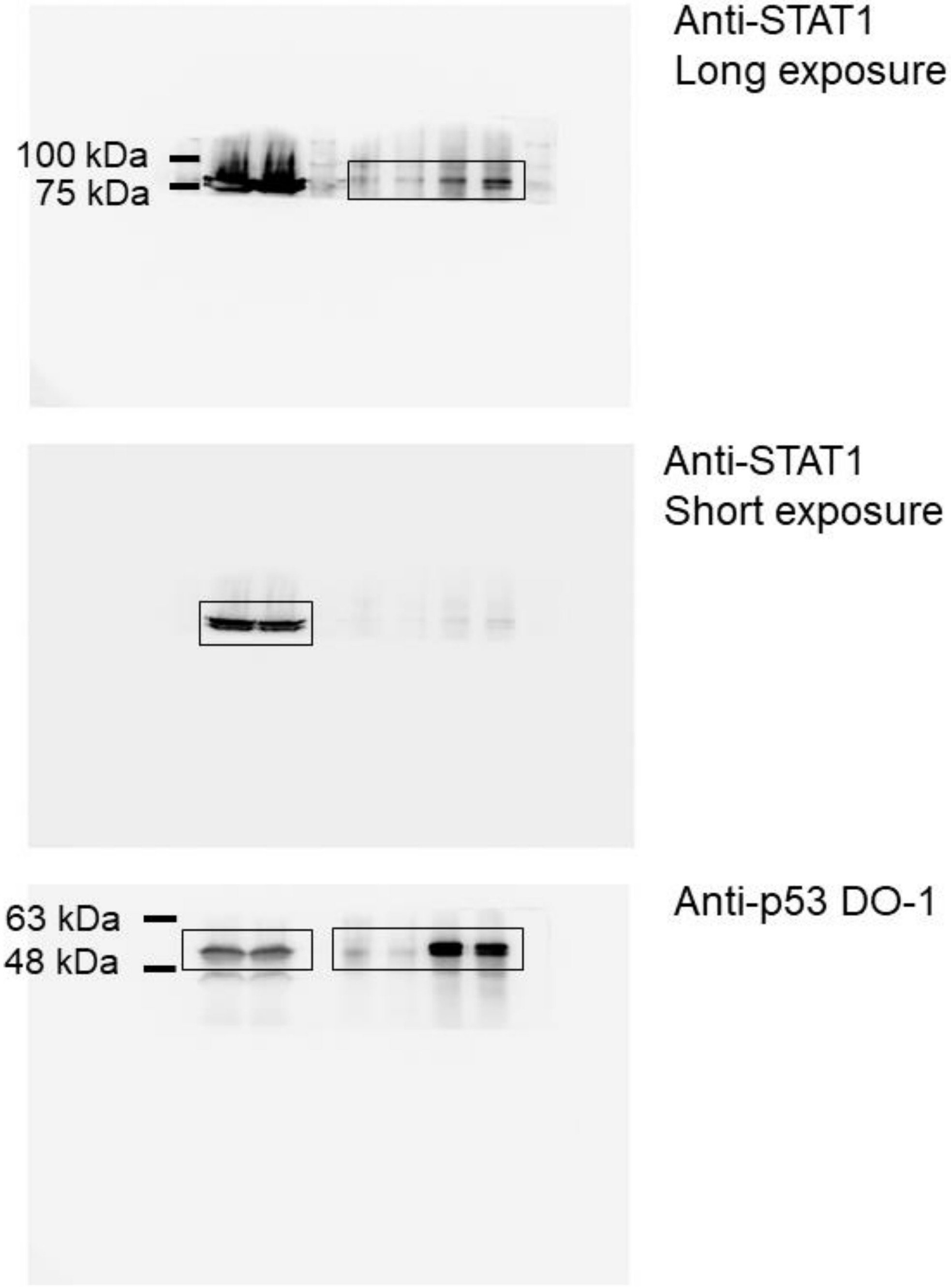
100 kDa
75 kDa
Anti-STAT1
Long exposure
Anti-STAT1
Short exposure
63 kDa
48 kDa
Anti-p53 DO-1

**Figure 4C**

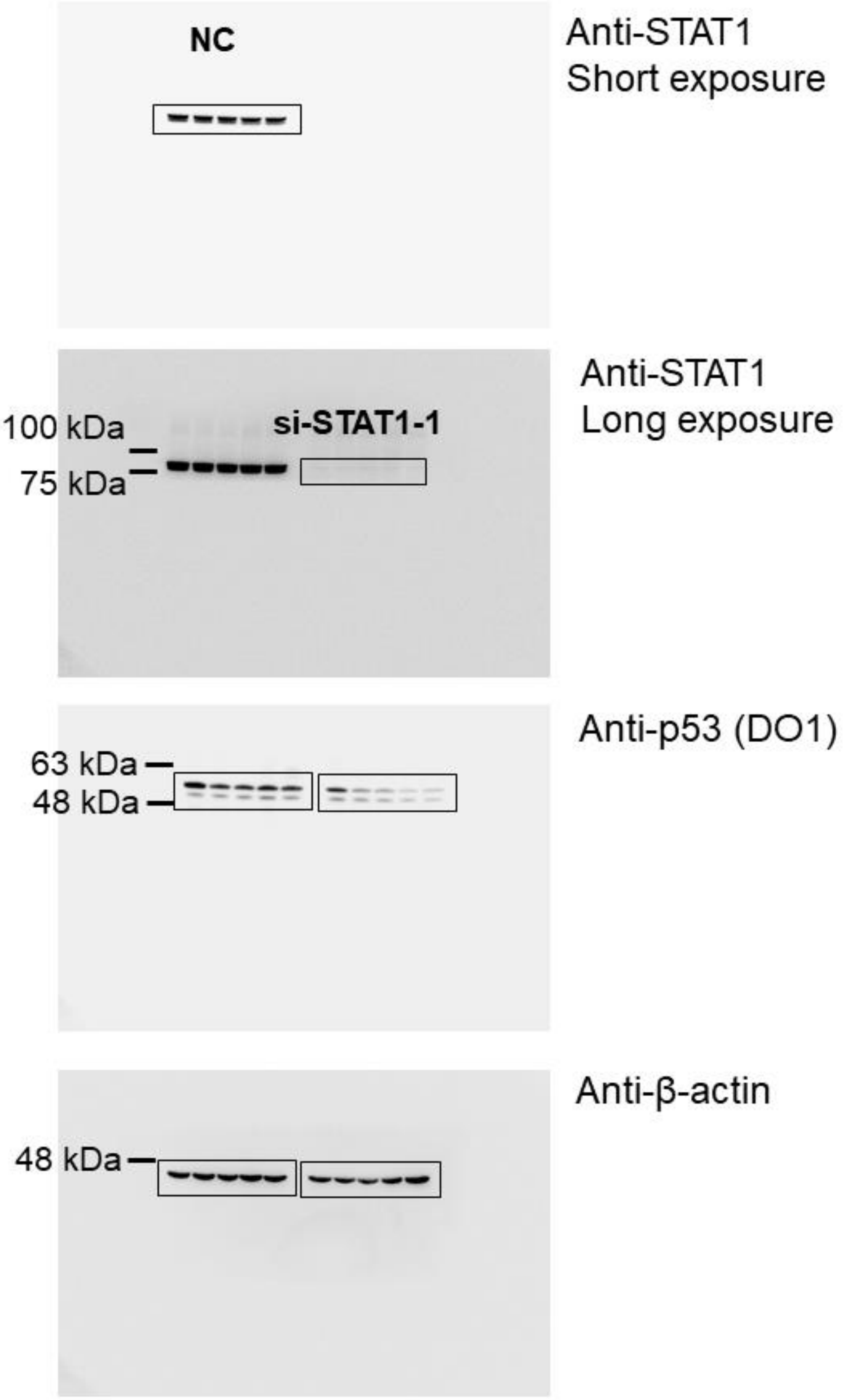
NC
Anti-STAT1
Short exposure
100 kDa
75 kDa
si-STAT1-1
Anti-STAT1
Long exposure
63 kDa
48 kDa
Anti-p53 (DO1)
48 kDa
Anti-β-actin

## Figure 4C_contnued

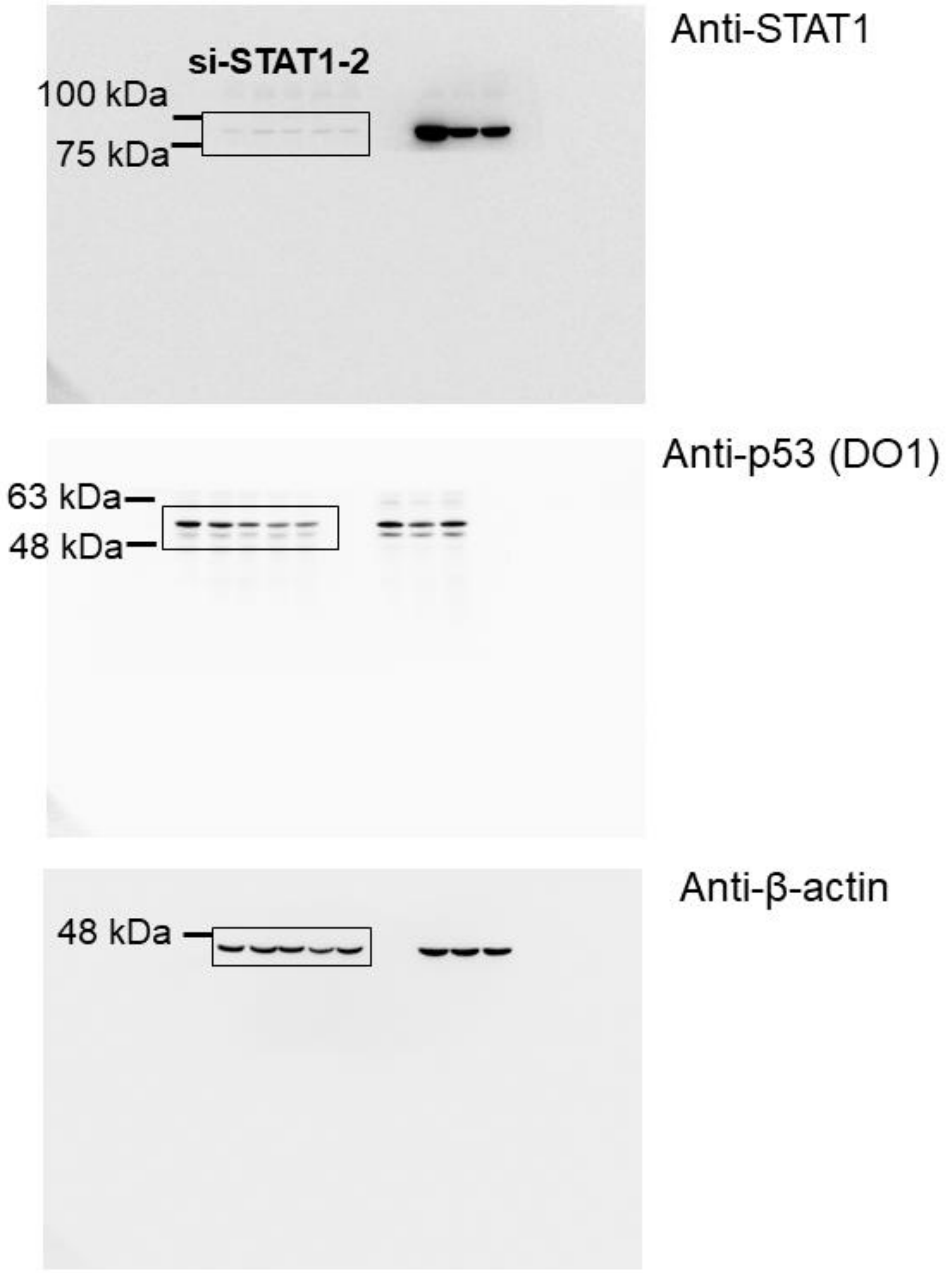

**Figure 4D**

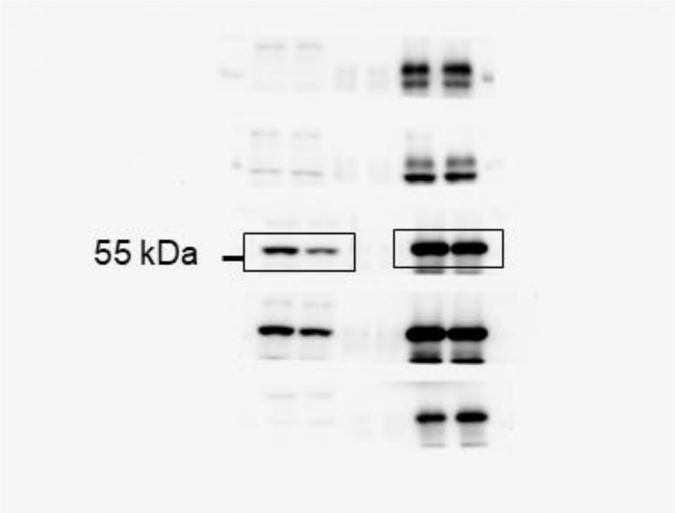


Anti-p53
(GTX102965)

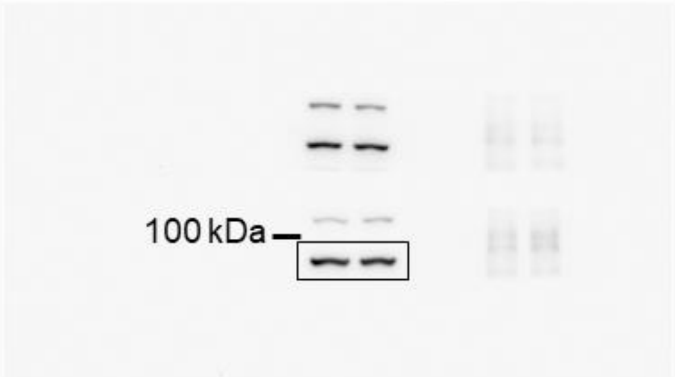


Anti-Mdm2

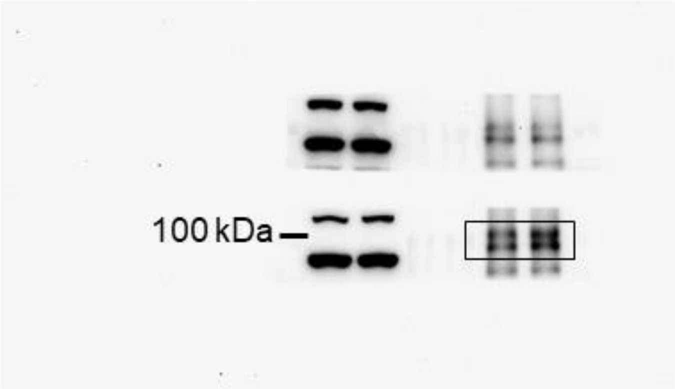


Anti-Mdm2
Longer exposure

**Figure 4D _continued**

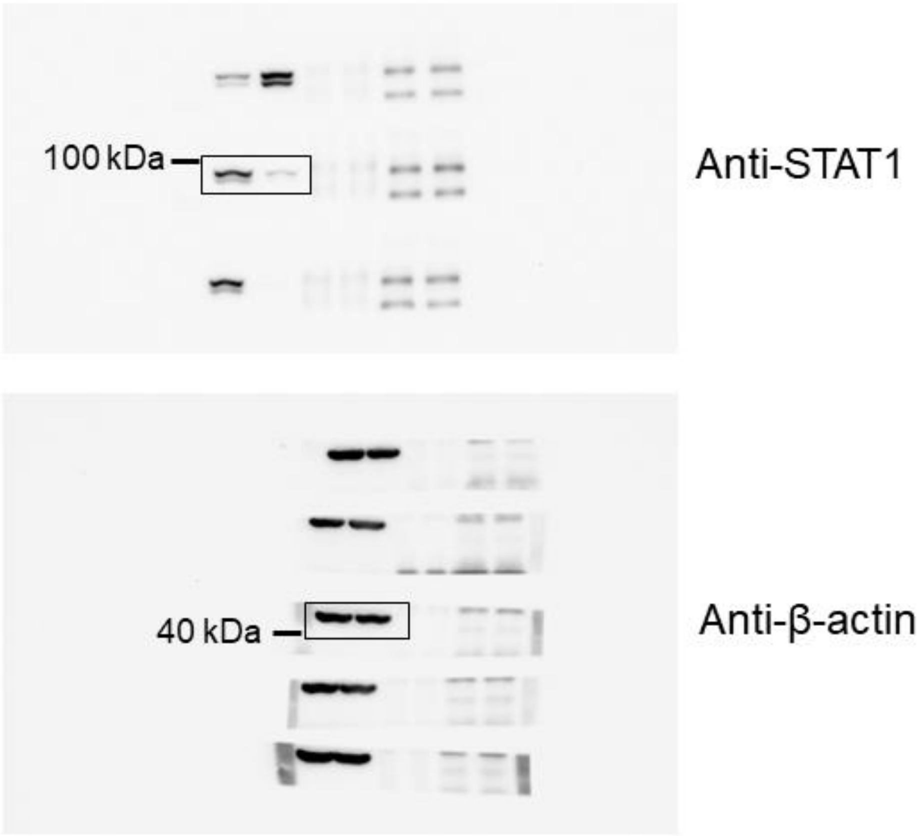

**Figure 4E**

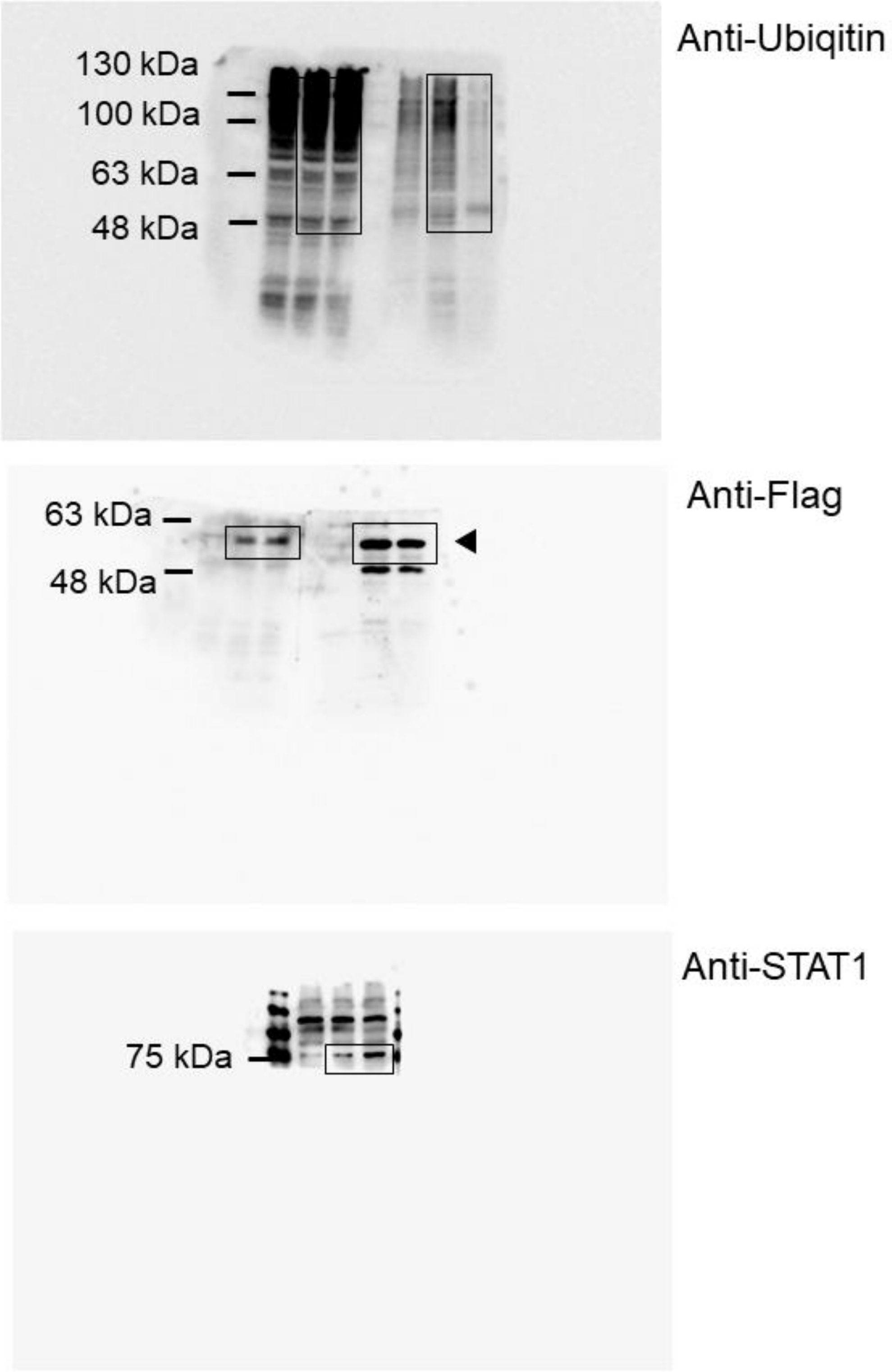

130 kDa
100 kDa
63 kDa
48 kDa
Anti-Ubiqitin
63 kDa
48 kDa
Anti-Flag
Anti-STAT1
75 kDa

**Figure 4F**

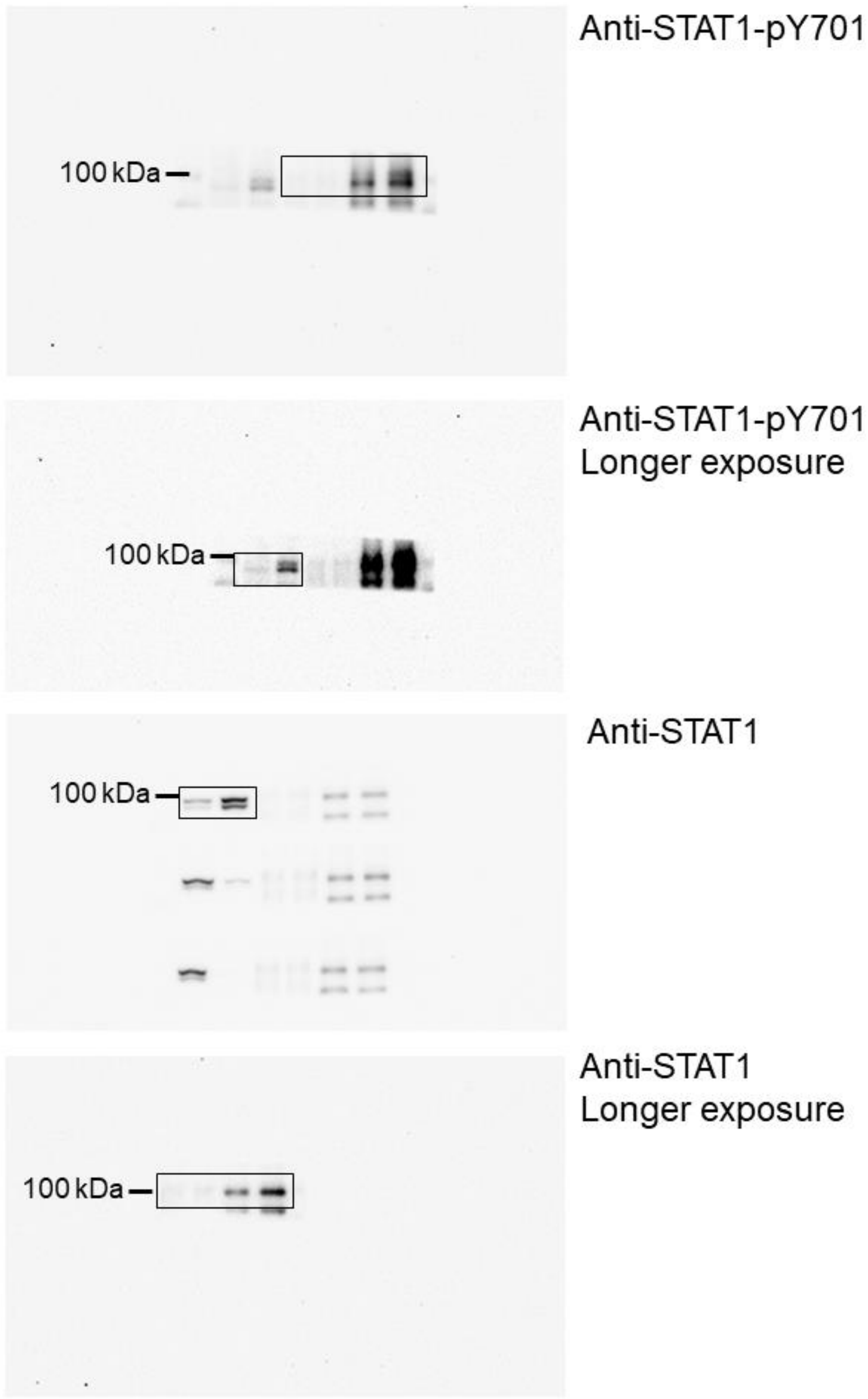
Anti-STAT1-pY701
100 kDa
Anti-STAT1-pY701
Longer exposure
100 kDa
Anti-STAT1
100 kDa
Anti-STAT1
Longer exposure
100 kDa

## Figure 4F _continued

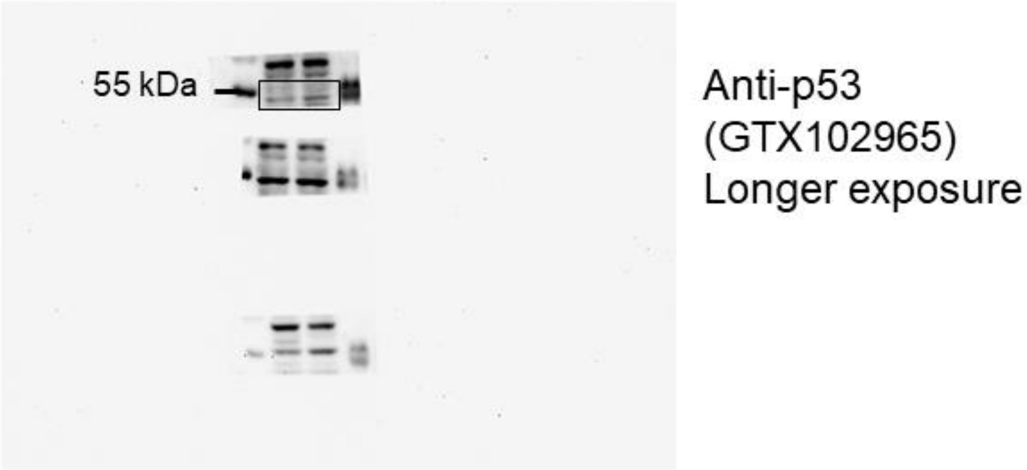


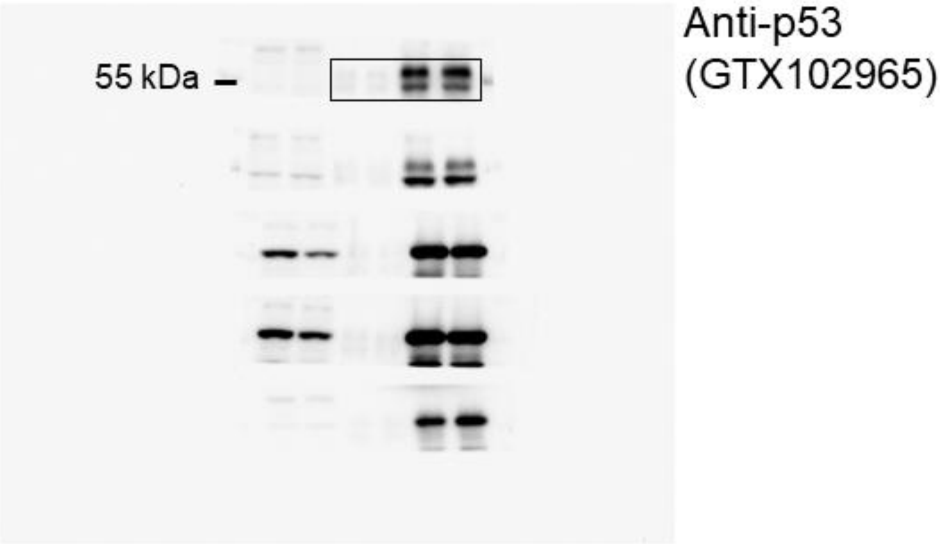


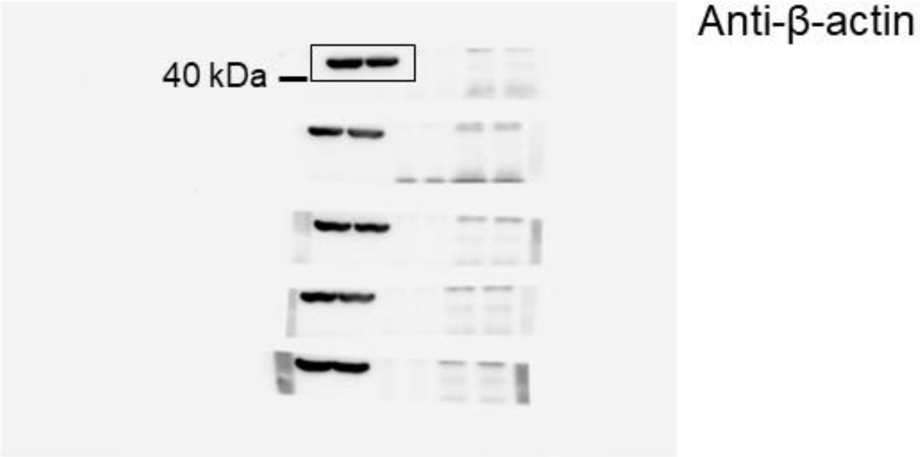

## Figure 4G

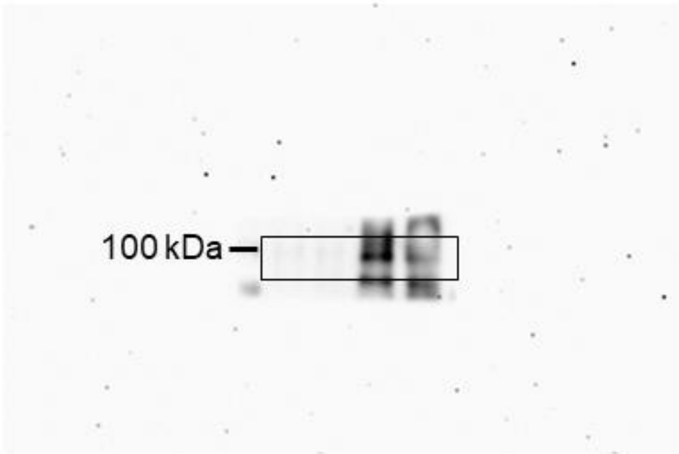


Anti-STAT1-pY701

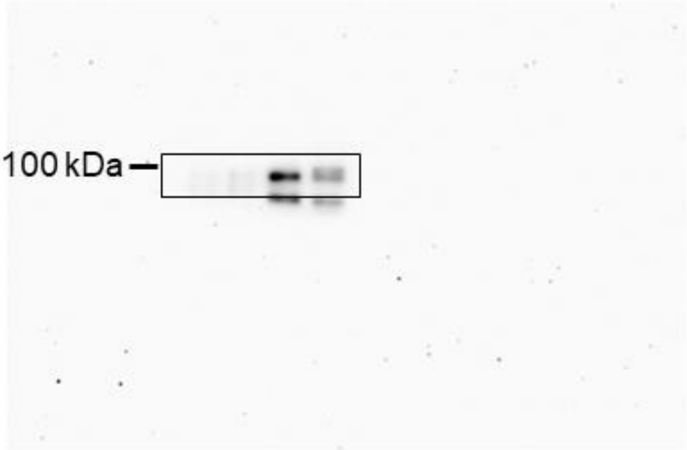


Anti-STAT1

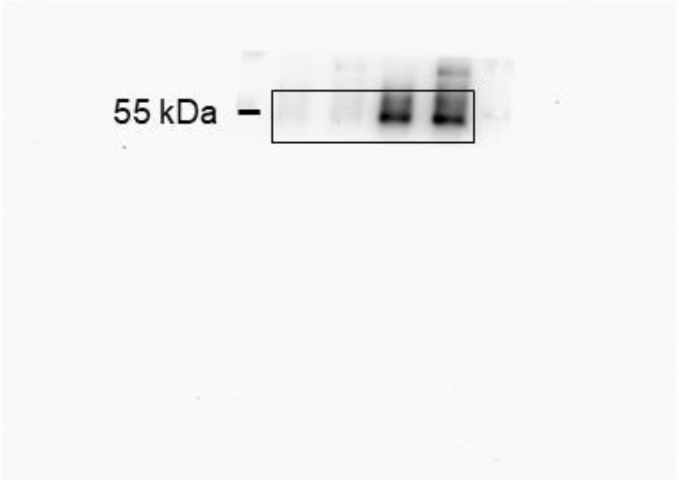


Anti-p53 (GTX102965)

## Figure 4G _continued

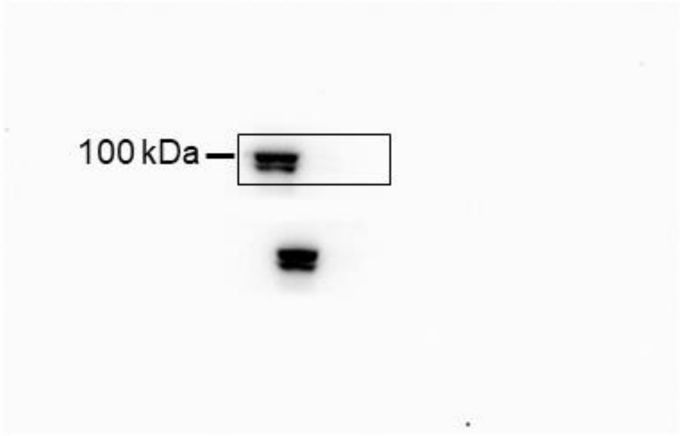


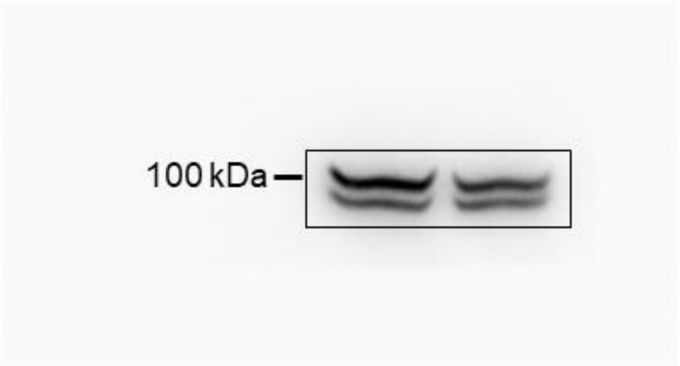


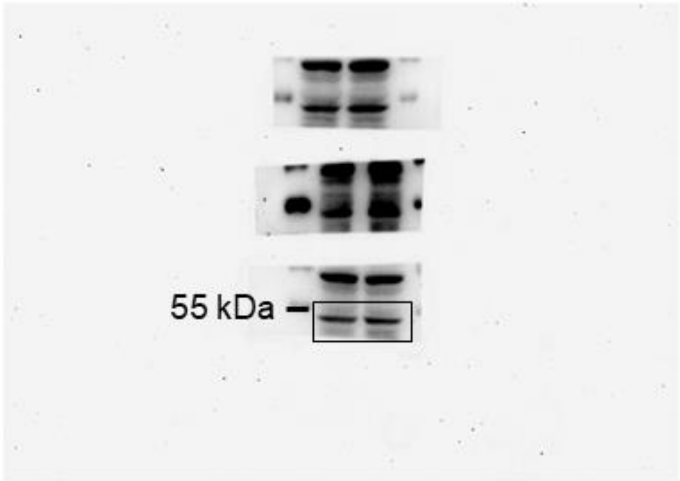


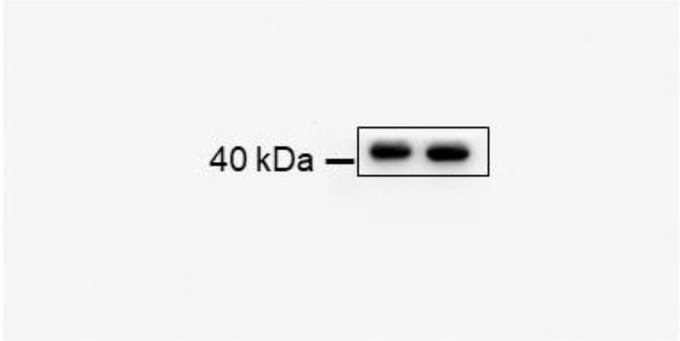

**Figure 4H**

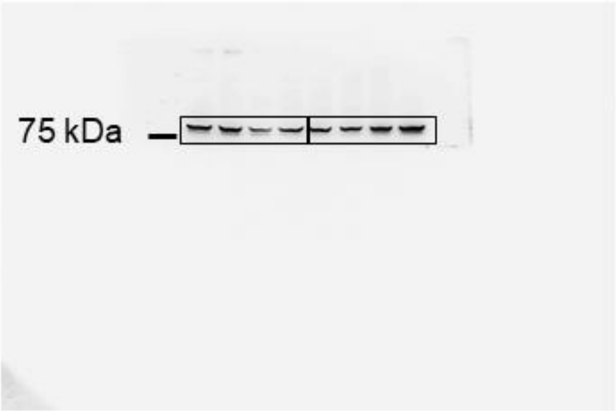


Anti-STAT1

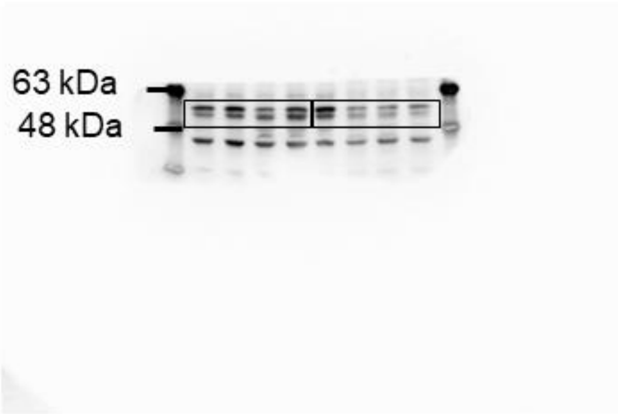


Anti-p53 (DO1)

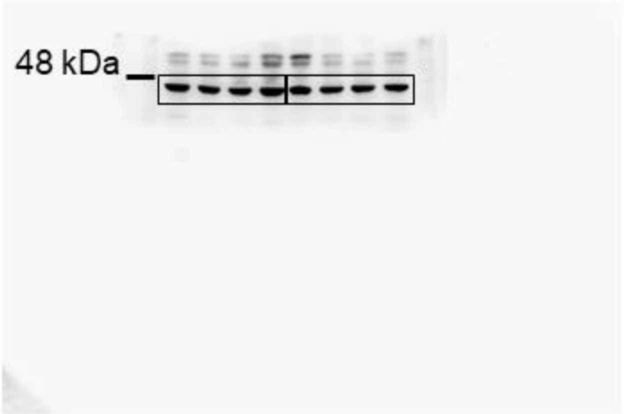


Anti-β-actin

# Figure 4I

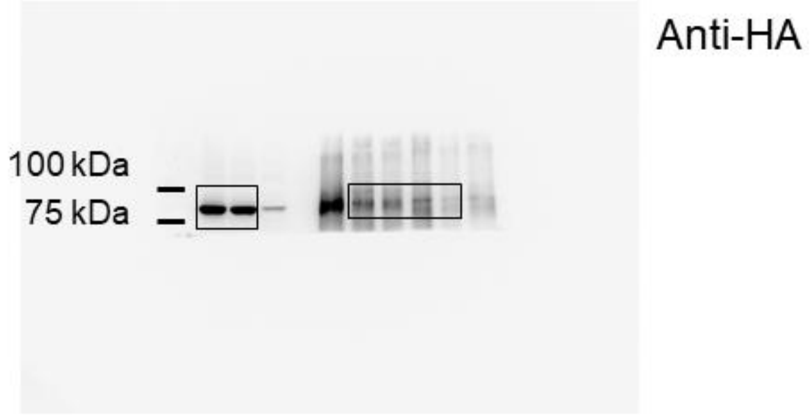


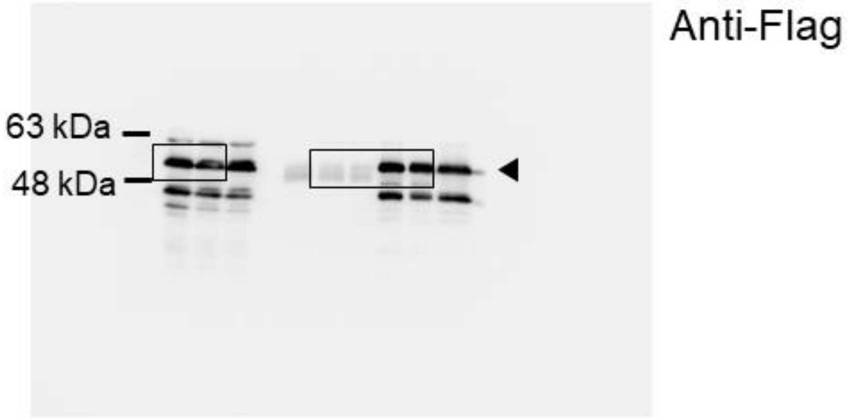

Supplement: Supplementary file 3 — WB_Raw data [file 41419_2025_7346_MOESM3_ESM.pdf]
